# Asymmetric Synthesis of β-Substituted α-Methylenebutyro- lactones *via* TRIP-Catalyzed Allylation: Mechanistic Studies and Application to the Synthesis of (*S*)-(−)-Hydroxymatairesinol

**DOI:** 10.1002/adsc.201300392

**Published:** 2013-08-29

**Authors:** Michael Fuchs, Markus Schober, Andreas Orthaber, Kurt Faber

**Affiliations:** a Department of Chemistry, University of Graz,Heinrichstraße 28, 8010 Graz, Austria, Fax: (+43)-(0)316-380-9840; phone: (+43)-(0)316-380-5332; b Department of Chemistry, Ångström Laboratories,Uppsala University, Box 523, 75120 Uppsala, Sweden, Fax: (+46)-018-471-6844; phone: (+46)-018-471-6585

**Keywords:** asymmetric allylation, butyrolactones, chiral phosphoric acids, hydroxymatairesinol, organozinc reagents

## Abstract

Asymmetric allylation of (hetero)aromatic aldehydes by a zinc(II)-allylbutyrolactone species catalyzed by a chiral BINOL-type phosphoric acid gave β-substituted α-methylenebutyrolactones in 68 to >99% *ee* and 52–91% isolated yield. DFT studies on the intermediate Zn^2+^-complex – crucial for chiral induction – suggest a six-membered ring intermediate, which allows the phosphoric acid moiety to activate the aldehyde. The methodology was applied to the synthesis of the antitumour natural product (*S*)-(−)-hydroxymatairesinol.

Asymmetric allylation is a key transformation in organic synthesis, which has been enabled through addition of various organometallic species onto aldehydes and ketones forming up to two chiral centers in a single step. A variety of methodologies has been developed, mainly using allylboronates or silanes in combination with stoichiometric or catalytic amounts of the chiral reagent or mediator.^1^ However, the asymmetric synthesis of butyrolactone-based natural products (e.g., lignans) *via* allylation of aldehydes still remains troublesome,^2^ although racemic methods have been recently developed.^3^ Only a few catalytic approaches exist.^4^ Allylboronates have proven to be a unique tool for asymmetric allylation, but the preparation of lactono-based allylboronates – required for the preparation of the title compound class – is unknown. Allyl-Sn and allyl-Si esters, another widely used class of allylating reagents, require additional Lewis acid activation during the allylation step,^5^ which can harm the lactone scaffold. Therefore, new methodologies are desirable, whereby stereoselectivity is induced through reactants which show a broad functional group tolerance including esters and lactones. For racemic allylation variants, organozinc compounds are regularly used, because they are easily prepared from the corresponding allylic halides and zinc dust^6^ and show a high tolerance towards a variety of functional groups.^7^ To date, reports on the asymmetric Zn^2+^-mediated Barbier-type allylation reaction are scarce,^8^ although high diastereoselectivity can be induced regarding the *syn*:*anti* ratio of the two chiral centers formed during the allylation with butyrolactone **4** (Scheme 1).^3^ A possible explanation for the lack of asymmetric protocols may be that most reactions are run in highly coordinating and/or polar solvents (such as THF, DMF, DME or even water) which is essential for metal insertion into the carbon-halide bond. On the other hand, polar media enhance the reaction rate of the uncatalyzed (achiral) reaction by impeding the coordination of chiral additives and thus interfere with chirality transfer. However, catalytic methods involving chiral zinc catalysts have been reported recently,^9^ demonstrating that chiral induction by organozinc compounds is possible.

**Scheme 1:**
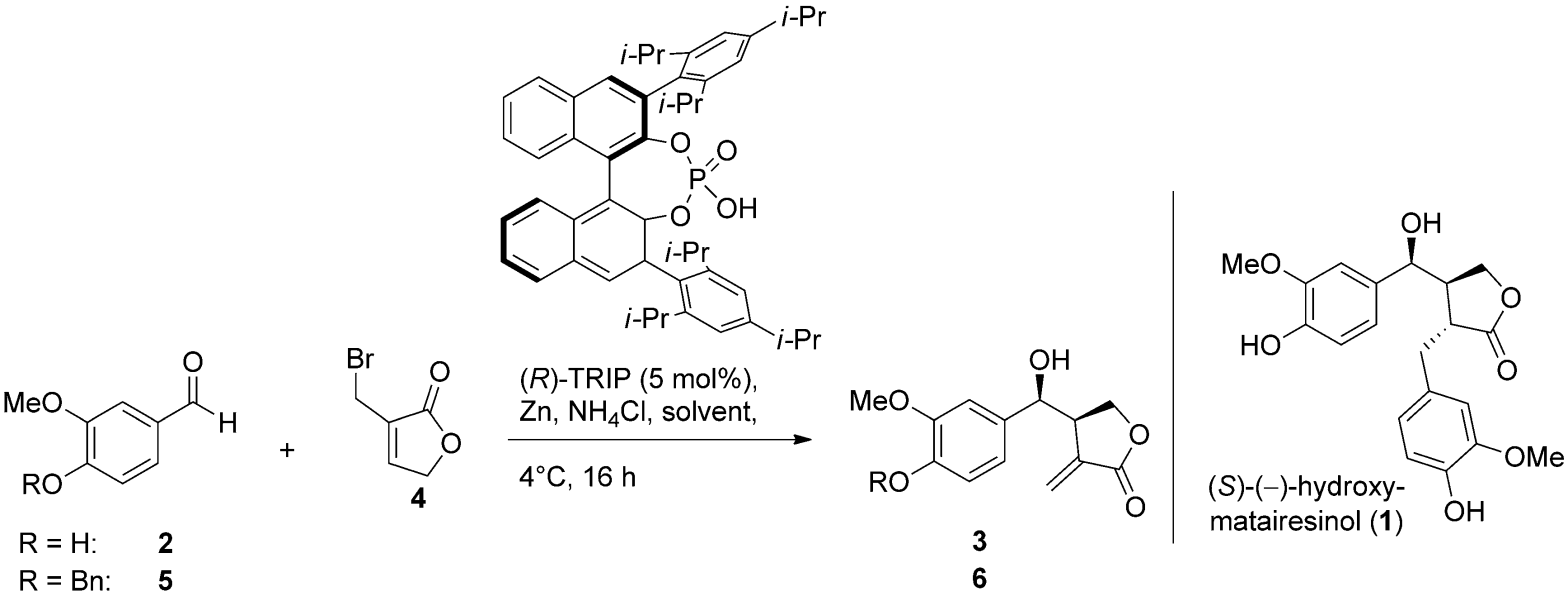
Model reaction for optimization of the asymmetric allylation.

Our study was initiated by the identification of an appropriate catalyst for our model reaction, using aldehyde 2, which serves as precursor for (*S*)-(−)-hydroxymatairesinol (1) (Scheme 1).^3^ Chiral phosphoric acids have emerged as a new class of organocatalysts since the pioneering work of Akiyama^10^ and Terada^11^ and their tremendous potential as chiral reagents has sparked many investigations.^12^ In this context, Antilla and Jain have recently applied chiral phosphoric acids, such as 3,3′-bis(2,4,6-triisopropylphenyl)-1,1′-binaphthyl-2,2′-diyl hydrogenphosphate (TRIP),^13^ to the asymmetric allylboration of aldehydes with high stereoselectivities for C—C bond formation.^14^ Although mechanistic proposals for phosphoric acid catalysis involving aldehydes are rare,^15^ we identified (*S*)-TRIP as being a potential catalyst candidate since initial experiments indicated that reasonable amounts of product with promising levels of enantiomeric excess (*ee* 20 %, *syn*:*anti* 9:91) are formed in toluene/THF at a catalyst loading of 5 mol% (Table 1, entry 1). The addition of NH_4_Cl for acidic activation of the Zn surface in allylation reactions^3^ was essential; no product formation was observed in its absence. Noteworthy, activation according to Knochel et al. (TMSCl+1,2-dibromoethane)^16^ showed positive effects on the conversion, but gave racemic product. Upon addition of the potassium salt of the chiral phosphoric acid we observed declining conversion and enantioselectivity (66% conversion and 10% *ee*, compare with Table 1, entry 1). Changing the inserting metal to indium slowed down the uncatalyzed reaction. However, no chiral induction was found upon addition of the chiral phosphoric acid. Experiments using the preformed phosphoric acid salt (TRIP^−^ NH_4_^+^) showed reduced conversions and stereoselectivities. Alternatively, the Zn^2+^-TRIP species (acting as a catalytically active Lewis acid) would lead to a transition state intermediate *via* single coordination with a long distance beween the chirality-inducing (*i-*Pr)_3_C_6_H_2_ ligands and the lactone moiety, which could not explain the high (dia)stereoinduction experimentally observed20a (see Figures S01 and S02 in the Supporting Information).

**Table 1:** Optimization of asymmetric allylation.[a]

Entry	Solvent	R=	Conversion [%][b]	*syn*:*anti*[c]	*ee* [%][d]
1	toluene/THF 1/1	H	76	9:91	20
2	toluene/EtOH 1/1	H	90	8:92	<1
3	toluene/1-decanol 1/1	H	41	12:88	12
4	toluene/NMP 1/1	H	67	4:96	<1
5	toluene/DME 1/1	H	>99	5:95	<1
6	toluene/DME 99.5/0.5	H	8	10:90	75
7	toluene/MTBE 1/1	H	22	<1:>99	80
8	CH_2_Cl_2_/Et_2_O 1/1	H	40	9:91	70
9	CH_2_Cl_2_/Et_2_O 2/1	H	57	12:88	55
10	CH_2_Cl_2_/(*i-*Pr)_2_O 1/1	H	15	11:89	73
11	CH_2_Cl_2_/(*n-*Bu)_2_O 1/1	H	20	14:86	64
12	CH_2_Cl_2_/Ph_2_O 1/1	H	17	9:91	71
13	CH_2_Cl_2_/toluene/Et_2_O 1/2/1	H	72	8:92	82
14[e]	toluene/(*i-*Pr)_2_O 1/1	H	48	3:97	80
15	toluene/Et_2_O 1/1	H	17	7:93	84
16[f]	toluene/(*i-*Pr)_2_O 1/1	Bn	45	<1:>99	91
17[g]	toluene/(*i-*Pr)_2_O 4/1	Bn	76	<1:>99	93
18^[g,h]^	toluene/*i*Pr_2_O 4/1	Bn	69	<1:>99	95
19[i]	toluene/(*i-*Pr)_2_O 4/1	Bn	79	<1:>99	98

[a] *Reaction conditions:* aldehyde **2** (entries 1–15) or **5** (entries 16–19, 40 mM), bromolactone **4** (1.5 equiv.), Zn dust (1.75 equiv.), NH_4_Cl (3.5 equiv.), 4 °C, 720 rpm, 16 h.

[b] Conversions were determined *via* HPLC-UV at 215 nm.

[c] The *syn*:*anti* ratios were determined *via* HPLC-UV analysis.

[d] The *ee*s were determined *via* HPLC-UV analysis on a chiral stationary phase.

[e] 50 mol% of (*S*)-TRIP.

[f] 10 mol% of (*S*)-TRIP and 4 equiv. of NH_4_Cl.

[g] 10 mol% of (*S*)-TRIP, 8 equiv. of NH_4_Cl and 2 equiv. of **4**.

[h] Experiment was conducted at −30 °C.

[i] 20 mol% of (*S*)-TRIP, 8 equiv. of NH_4_Cl and 2 equiv. of **4**.

In summary, these observations lead us to the conclusion that the catalytically active species would be the protonated phosphoric acid, coordinating the zinc core *via* the P—O moiety (Figure 1). Since acceleration of the same reaction by Brønsted acids has been previously observed, we assume that the aldehyde is activated *via* the acidic P—OH.3a

**Figure 1:**
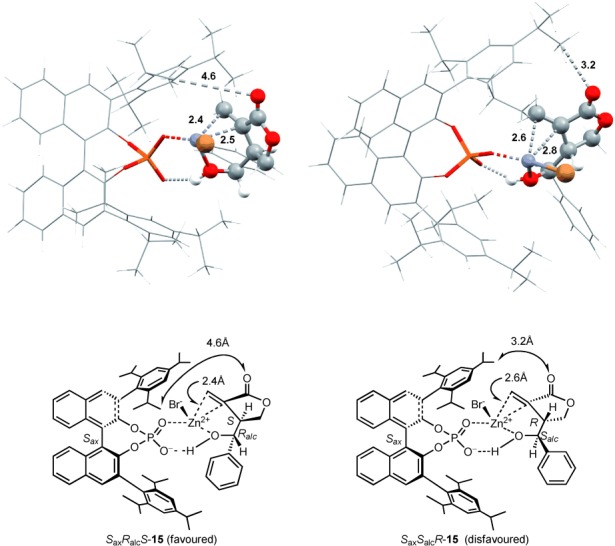
Favoured transition state *S*_ax_*R*_alc_*S*-15 (*left*) and disfavoured *S*_ax_*S*_alc_*R*-15 (*right*).

Since the reaction medium was expected to be an important parameter, careful optimization studies, especially of the polar component, were conducted. The most important results are summarized in Table 1 (for detailed information see the Supporting Information): Compared to the initial results with toluene/THF, ethanol gave high yields but only racemic product (entry 2). An extension of the alkane chain (1-decanol) showed improved selectivity in combination with a decreased conversion, implying that either steric issues in the coordination of the transition state intermediate or the dipole moment of the solvent were important for the stereoselectivity (entry 3). Solvents with higher coordination strength/dipole, such as NMP (*N*-methylpyrrolidone) and DME (1,2-dimethoxyethane) completely destroyed the chiral induction due to impeded ligand exchange on the zinc metal core (entries 4 and 5). However, at reduced levels of DME (0.5%), elevated *ee*s were observed again going in hand with diminished activity (entry 6). Non-coordinating ethers, such as MTBE (methyl *tert*-butyl ether) gave high selectivities, but low conversions (entry 7). In pure MTBE the *ee* was decreased without an increase in conversion. Dichloromethane proved to be an attractive alternative to toluene, as conversion was almost doubled with a slight decline in stereocontrol (entries 8 and 13). Since diethyl ether had emerged as a potential polar candidate, similar ethers were tested. Based on their steric properties it became clear that the inner coordination sphere of the zinc center seemed to have the highest influence, as ethers with higher steric hindrance had a positive influence on the stereoinduction rather than the alkyl chain length (entries 8–12). Further increase in selectivity was achieved when toluene was added again and these ternary solvent mixtures gave the first *ee* above 80% at reasonable levels of conversion (entry 13). Since alcohols (including phenol) led to a dramatic decline of stereocontrol, we envisaged to protect the phenol moiety on substrate **2** in order to exclude such negative effects by the substrate’s OH moiety. Benzyl-protected vanilline **5**, was converted with an *ee* of 91% (entry 16) and a reasonable yield.

In order to further increase the conversion, we raised the amounts of reagents and used less of the polar solvent, which pushed stereoselectivities even further (entries 17 and 18). The last boost to the optimization was accomplished by employing a higher catalyst loading (20 mol%) giving the precursor of **1** in almost perfect stereoselectivity (*ee* 98%, *syn*:*anti* <1:>99) and an acceptable isolated yield (Table 1, entry 19, Table 2, entry 1). Comparison of entries 14 (R=H, 50 mol% catalyst, *de* 94%, *ee* 80%) and 16 (R=Bn, 10 mol% catalyst, *de* >99%, *ee* 91%) clearly shows that the presence of the benzyl protective group was beneficial with respect to stereoselectivities and catalyst loading.

**Table 2:** Substrate scope of asymmetric allylation.[a] 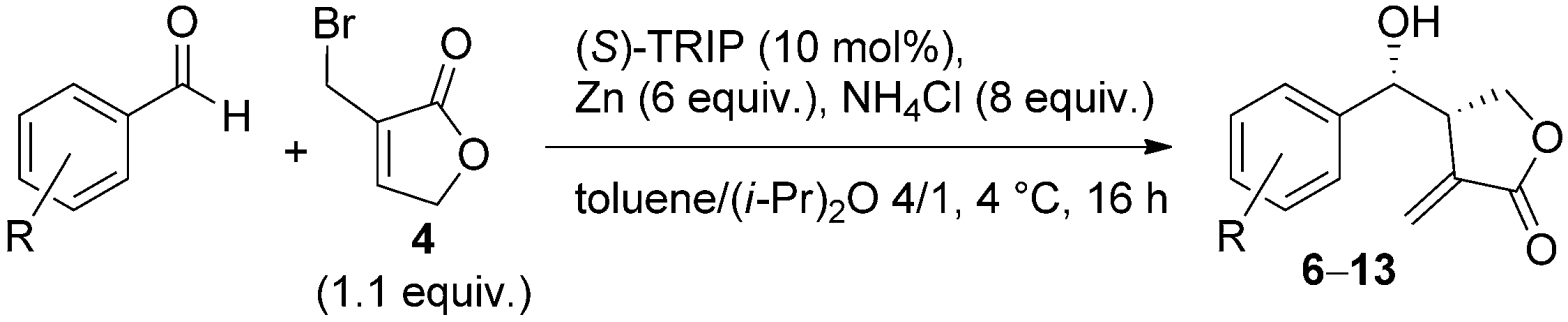

Entry	Product	Conversion [%][b]	*syn*:*anti*[c]	*ee* [%][d]
1[e]	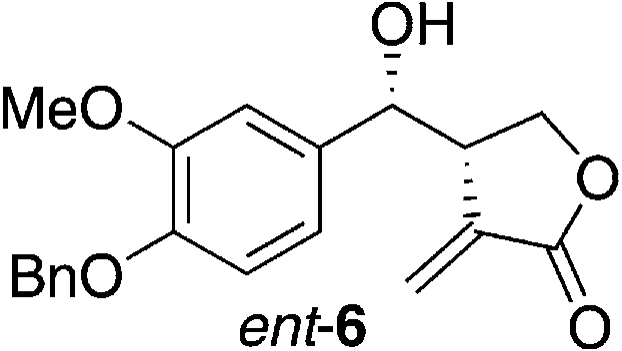	73 (71)	<1:>99	90
		73 (71)[f]	<1:>99[f]	98[f]
2	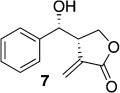	91 (88)	<1:>99	94
3	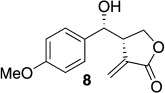	84 (79)	<1:>99	96
4	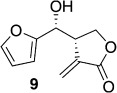	>99 (91)	14:86	n.d.[g]
5	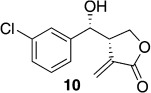	77[h]	<1:>99	96
6	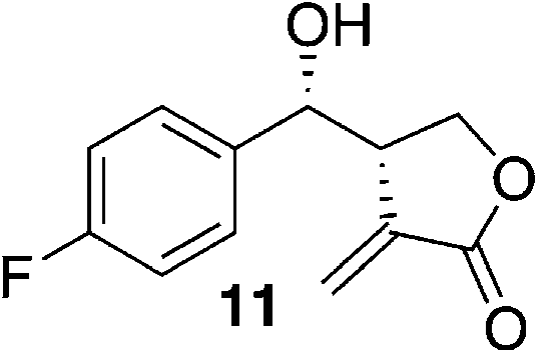	88[h]	<1:>99	97
7	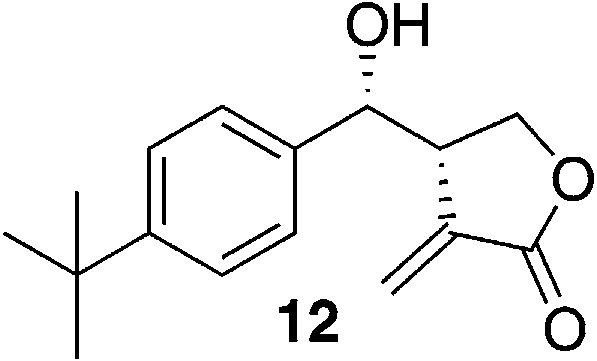	95 (91)	<1:>99	>99
8	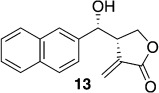	86 (80)	<1:>99	97

[a] *Reaction conditions:* aldehyde (20 mM), bromolactone **4** (1.1 equiv.) Zn dust (6 equiv.), NH_4_Cl (8 equiv.), toluene/(*i-*Pr)_2_O 4/1, 4 °C, 720 rpm, 16 h.

[b] Conversions were determined via HPLC-UV at 215 nm, isolated yields are given in brackets.

[c] The *syn*:*anti* ratio was determined *via* ^1^H NMR.

[d] The *ee* was determined *via* HPLC-UV analysis on a chiral stationary phase.

[e] 2 equiv. of **4** were used.

[f] (*R*)-TRIP (20 mol%) gave **6**.

[g] Not determined due to inseparable *syn*:*anti*-mixture.

[h] No isolated yields are given due to inseparable minor impurities (for details see Supporting Information).

Having developed a practicable protocol for the asymmetric synthesis of lactone **6**, we investigated the substrate scope and the upscaling of the reaction (Table 2). All further substrates tested were nicely converted with 1.1 equivalents of bromolactone **4** at reduced catalyst loadings (10 mol%). Electron-rich aldehydes showed similar conversion levels as electron-deficient analogues (entries 3, 5, 6). The only limitation regarding aromatic residues was found in the allylation of furfural, which gave decreased *syn*:*anti*-stereocontrol (entry 4). Since similar effects were observed using THF as solvent, we assume that zinc-coordination by oxygen atoms accelerates the reaction in a non-selective fashion.

Aliphatic aldehydes reacted with good conversion (∼90%) but showed low enantioselectivities, and double bond isomerization was observed for alkenyl derivatives (e.g., cinnamic aldehyde), where conversions were in the range of ∼60% accompanied by isomerization products (∼20–30%). Bulky residues (e.g., 4-*tert*-butylphenyl, naphthyl, entries 7 and 8) were converted in good yields with very high stereopreference. Noteworthy, reactions performed under anhydrous conditions gave the same results as batches in moist solvents under air. The absolute configuration of **6** was determined *via* asymmetric synthesis^14^ (see below), products **7**–**13** were elucidated by CD spectroscopy using **6** as reference (same Cotton effect, see Supporting Information).

In order to demonstrate the applicability of the method, we transformed precursor **6**, obtained from (*R*)-TRIP-catalyzed allylation, to the natural product (*S*)-(−)-hydroxymatairesinol (**1**)^17^ *via* a short sequence depicted in Scheme 2.3a The latter is used in clinical studies^18^ as a precursor to the mammalian lignan enterolactone, which exhibits antitumour activity.18a,^19^

**Scheme 2:**
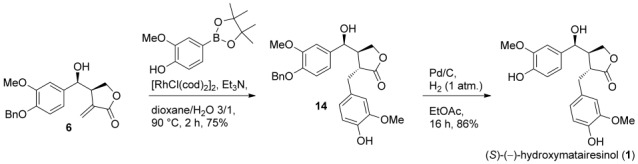
Synthesis of (*S*)-(−)-hydroxymatairesinol (1).

The stereochemical outcome of the asymmetric synthesis of **1** was opposite to that of the allylboration with the same catalyst^14^ owing to the different mechanism of the latter reaction, which requires acidic activation of the boronic ester.^20^ Based on the experimental results (*anti*-configured products) and the considerations on the catalyst’s nature (protonated phosphoric acid coordinated to zinc) DFT calculations yielded only model structure **15** as energetically and mechanistically reasonable intermediate (Figure 1), when stationary points were investigated using the M06 functional with a triple-ζ basis set (6-311G**).^21^ The structure **15** consists of a double coordination of the P(O)OH group to the zinc-allyl-aldehyde ring system in a chair-like transition state (Zimmerman–Traxler model). Both six-membered rings are distorted and zinc coordinates the allylic carbon atom. However, the distance to the adjacent C atom is in the order of 2.4 to 2.8 Å, forecasting the C—C bond to be formed and causing a significant ring contraction. The phosphoric acid coordinates the Zn center through the P—O moiety, while the P—OH unit activates the aldehyde giving a flat six-membered ring (coordination of the phosphoric acid yields >200 kcal mol^−1^).^22^ With this concept about the intermediate’s geometry, we then focused on the mode of chiral induction during alcohol formation by investigating two stereochemical situations: *S*_ax_*S*_alc_*R* and *S*_ax_*R*_alc_*S*.^23^ The combination *S*_ax_ and *R*_alc_*S* was favoured by 1.7 kcal mol^−1^, which translates to an *ee* of 91% (experimentally observed 94%). Careful analysis of the calculated structures (Figure 1) did not reveal any significant steric hindrance between the phenyl substituent of the substrate and the catalyst in both isomeric structures. However, steric repulsion can be found between the isopropyl moieties of the ligand and the lactone (4.6 *vs.* 3.2 Å), which results in shorter Zn-allyl-carbon distances in the favoured *S*_ax_*R*_alc_*S* structure (2.4 *vs.* 2.6 Å; Figure 1).

In order to comprise contributions from non-catalyzed turnovers, stabilization of the proposed intermediate by coordination of different ethers [Me_2_O, Et_2_O, (*i-*Pr)_2_O and THF] was assessed by means of continuum models (IEF-PCM). Overall, stabilization by a single solvent molecule lies within the range of *ca.* −13 to −15 kcal mol^−1^, with a minimum energy for THF (for details see the Supporting Information). Therefore, an acceleration of the non-catalyzed reaction by higher coordinating ethers can be deduced, explaining the reduced *ee* value in presence of these solvents.

In conclusion, the catalytic asymmetric allylation of aldehydes by allylzinc species using a chiral, BINOL-derived phosphoric acid (TRIP) was demonstrated. Careful choice of the reaction conditions gave high isolated yields and almost perfect enantio- and diastereoselectivities. The preparative scale applicability of the method was demonstrated by the total synthesis of (*S*)-(−)-hydroxymatairesinol (**1**) in 98% *ee* and 46% overall yield, starting from aldehyde **5** (including its benzyl protection). Finally, a proposal for the transient species was developed by DFT calculations, which explains the stereochemical outcome due to steric interaction of the lactone residue with the ligand of the TRIP-catalyst.

## Experimental Section

### General Procedure for the Asymmetric Allylation

A 50-mL round-bottom flask, equipped with a magnetic stir bar, was charged with zinc dust (109 mg, 1.67 mmol), NH_4_Cl (130 mg, 2.4 mmol) and (*S*)-TRIP (22 mg, 0.029 mmol). Toluene (12 mL, precooled to 4 °C), (*i-*Pr)_2_O (3 mL, precooled to 4 °C), the corresponding aldehyde (0.29 mmol) and bromolactone **4** (35 μL, 58 mg, 0.33 mmol) were added, the flask was closed with a stopper equipped with a Teflon ring and parafilm to avoid water condensation and stirred at 4 °C in a fridge for 16 h. The slurry was concentrated under reduced pressure to about 4 mL, which were directly applied to column chromatography on silica gel with toluene/THF 8/1 as eluent except where stated otherwise.

## Notes

As a service to our authors and readers, this journal provides supporting information supplied by the authors. Such materials are peer reviewed and may be re-organized for online delivery, but are not copy-edited or typeset. Technical support issues arising from supporting information (other than missing files) should be addressed to the authors.
