# Supplementary material for: Asymmetric Synthesis of β-Substituted α-Methylenebutyro- lactones via TRIP-Catalyzed Allylation: Mechanistic Studies and Application to the Synthesis of (S)-(−)-Hydroxymatairesinol
Source: Adv Synth Catal. 2013 Aug 29;355(13):2499–505. doi: 10.1002/adsc.201300392 (PMC3883096; doi:10.1002/adsc.201300392)
Supplement: Supplementary file 1 [file adsc0355-2499-sd1.pdf]

Advanced Synthesis & Catalysis

Supporting Information

© Copyright Wiley-VCH Verlag GmbH & Co. KGaA, 69451 Weinheim, 2013

Supporting Information

Asymmetric Synthesis of β-Substituted α-Methylene-Butyrolactones *via* TRIP-catalyzed Allylation: Mechanistic Studies and Application to the Synthesis of (*S*)-(-)-Hydroxymatairesinol

Michael Fuchs,[a] Markus Schober,[a] Andreas Orthaber,[*,b] Kurt Faber[*,a]

[a] *Department of Chemistry, Organic and Bioorganic Chemistry, University of Graz, Heinrichstraße 28, 8010 Graz, Austria, Fax: (+43)-(0)316-380-9840; phone: (+43)-(0)316-380-5332, e-mail: Kurt.Faber@Uni-Graz.at*

[b] *Department of Chemistry, Ångström Laboratories, Uppsala University, Box 523, 75120 Uppsala, Sweden, Fax*: (+46) 018 - 471 6844; phone: (+46) 018 - 471 6585, *e-mail: andreas.orthaber@kemi.uu.se*

Table of Contents

Additional optimization data	S2
Experimental procedures	S4
Determination of absolute configuration	S12
^{1}H- and ^{13}C-NMR spectra	S13
HPLC-UV Analysis on a chiral stationary phase	S27
CD-spectra	S34
Additional computational details	S39
References	S51

Additional Optimization Data

Table S01. Screening Results.

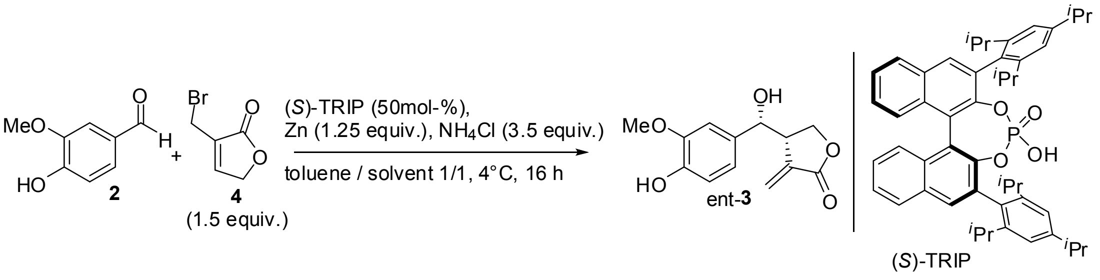

entry	solvent	conv. [%][a]	*syn*:*anti*[b]	ee [%][c]
1	THF	92	3:97	60
2	DMF	82	6:94	60
3	1,4-Dioxane	70	8:92	50
4	DME	>99	5:95	<1
5	$^{i}Pr_2O$	48	3:97	80

Reaction conditions: aldehyde (40 mM), bromolactone **4** (1.5 eq.) Zn (1.75 eq.), NH_4Cl (3.5 eq.), toluene/solvent 1/1, 4 °C, 720 rpm, 16 h; a) conversions were determined via HPLC-UV at 215 nm; b) *syn*:*anti* ratio was determined via HPLC-UV analysis; c) enantiomeric excess was determined via HPLC-UV analysis on a chiral stationary phase.

Table S02. Effects of Additives.

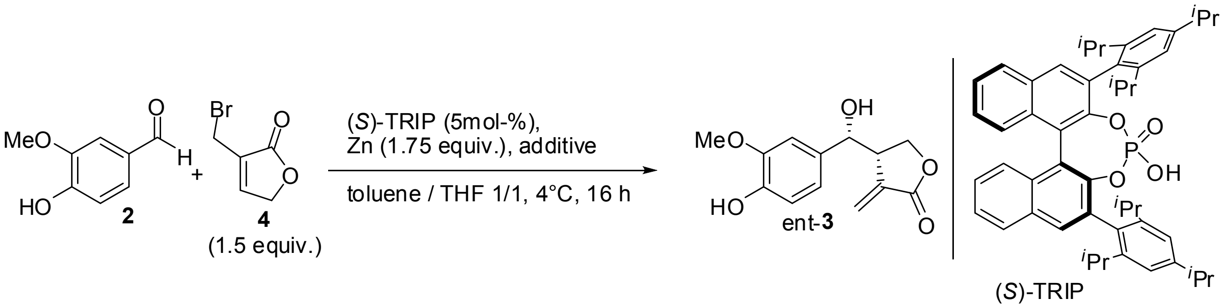

entry	additive	conv. [%][a]	*syn*:*anti*[b]	ee [%][c]
1	$CeCl_3$ (0.5 eq.)	19	21:79	<1
2	AcOH (0.5 eq.)	39	15:85	<1
3	BzOH (0.5 eq.)	3	n.d.	n.d.
4	LiBr (1 eq.)	64	6:94	<1
5[d]	pinacol (1 eq.)	15	5:95	69
6[e]	pinacol (25 eq.)	14	9:91	<1
7[e]	phenol (0.7 eq.)	3	18:82	<1

Reaction conditions: aldehyde (40 mM), bromolactone **4** (1.5 eq.) Zn (1.75 eq.), toluene/THF 1/1, 4 °C, 720 rpm, 16 h; a) conversions were determined via HPLC-UV at 215 nm; b) *syn*:*anti* ratio was determined via HPLC-UV analysis; c) enantiomeric excess was determined via HPLC-UV analysis on a chiral stationary phase; e) reaction was conducted in

toluene/Et_2O 4/1 in the presence of NH_4Cl (3.5 eq.); e) reaction was conducted in toluene only in the presence of NH_4Cl (3.5 eq.).

Table S03. Solvent Screening.

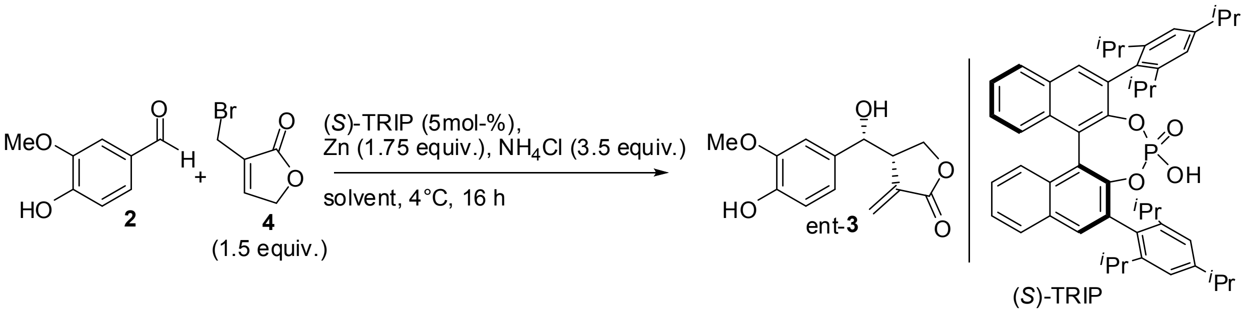

entry	solvent	conv. [%][a]	*syn*:*anti*[b]	ee [%][c]
1	toluene/THF 1/1	76	9:91	20
2	toluene/MeCN 1/1	48	10:90	10
3	toluene/MTBE 1/1	22	<1:>99	80
4	toluene/EtOAc 1/1	11	<1:>99	71
5	toluene/^{n}BuOH 1/1	88	11:89	6
6	toluene/*cyclo*-hexanol 1/1	40	11:89	8
7	toluene/1-decanol 1/1	41	12:88	12
8	toluene/DMA 1/1	77	4:96	<1
9	toluene/NMP 1/1	67	4:96	<1
10	toluene/EtOH 1/1	90	8:92	<1
11	MTBE	20	6:94	60
12	toluene/DME 1/1	>99	5:95	<1
	toluene/DME 99.5/0.5	8	10:90	75
13	CH_2Cl_2/MTBE 1/1	15	7:93	75
14	CH_2Cl_2/Et_2O 1/1	40	9:91	70
15	CH_2Cl_2/Et_2O 2/1	57	12:88	55
16	CH_2Cl_2/Et_2O 4/1	55	12:88	67
17	CH_2Cl_2/toluene/Et_2O 1/1/2	63	8:92	77
18	CH_2Cl_2/toluene/Et_2O 1/2/1	72	8:92	82
19	toluene/Et_2O 1/1	17	7:93	84
20	CH_2Cl_2/$^iPr_2O$ 1/1	15	11:89	73
21	CH_2Cl_2/$^nBu_2O$	20	14:86	64
22	CH_2Cl_2/Ph_2O	17	9:91	71

Reaction conditions: aldehyde (40 mM), bromolactone **4** (1.5 eq.) Zn (1.75 eq.), NH_4Cl (3.5 eq.), 4 °C, 720 rpm, 16 h; a) conversions were determined via HPLC-UV at 215 nm; b) *syn*:*anti* ratio was determined via HPLC-UV analysis; c) enantiomeric excess was determined via HPLC-UV analysis on a chiral stationary phase.

Table S04. Solvent and Additive Screening for Substrate 5.

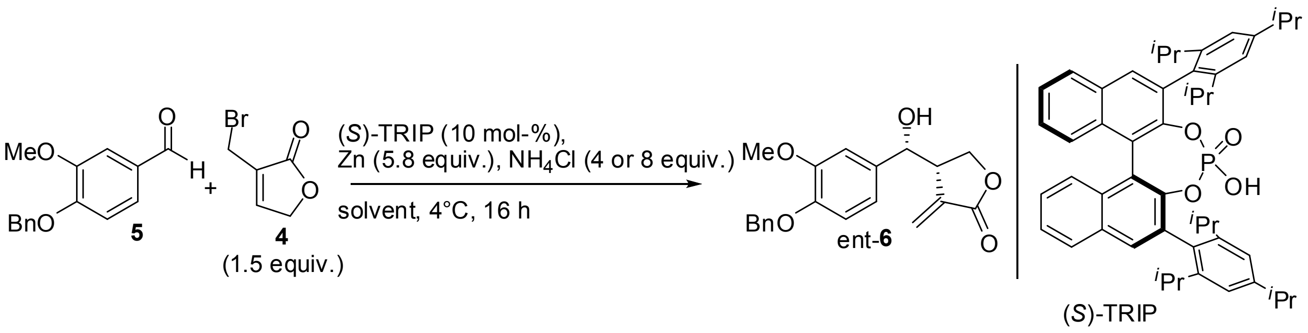

entry	solvent	conv. [%][a]	*syn*:*anti*[b]	ee [%][c]
1[d]	toluene/Et_2O 1/1	54	<1:>99	89
2[d]	toluene/$^{i}Pr_2O$ 1/1	45	<1:>99	91
3[e]	toluene/$^{i}Pr_2O$ 1/1	55	<1:>99	88
4[e,f]	toluene/$^{i}Pr_2O$ 3/1	73	<1:>99	91
5[e,f]	toluene/$^{i}Pr_2O$ 4/1	76	<1:>99	93
6[e,f,g]	toluene/$^{i}Pr_2O$ 4/1	79	<1:>99	98

Reaction conditions: aldehyde (20 mM), bromolactone **4** (1.5 eq.), Zn (5.8 eq.), 4 °C, 720 rpm, 16 h; a) conversions were determined via HPLC-UV at 215 nm; b) *syn*:*anti* ratio was determined via HPLC-UV analysis; c) enantiomeric excess was determined via HPLC-UV analysis on a chiral stationary phase; d) 4 eq. of NH_4Cl were employed; e) 8 eq. of NH_4Cl were employed; f) 2 eq. of bromolactone **4** were employed; g) 20 mol-% (*R*)-TRIP were employed and the opposite enantiomer was obtained.

Experimental Procedures

General. All chemicals were purchased from Sigma Aldrich, Acros Organics or Alfa Aesar and used as received, solvents were obtained from Roth. Zinc dust was from Sigma Aldrich (< 10 µm, ≥98% purity, catalog number 209988). All moisture- or air-sensitive operations were conducted under dry argon in heat-dried glassware. NMR-spectra were recorded on a Bruker NMR unit at 300 (^{1}H) and 75 (^{13}C) MHz, shifts are given in ppm and coupling constants (*J*) are given in Hz. GC-MS measurements were performed on an Agilent 7890A GC system, equipped with an Agilent 5975C mass-selective detector (EI 70 eV) and a HP-5-MS column (30 m x 0.25 mm x 0.25 µm film) using He at a flow rate of 0.55 mL/min. Temperature program: 100 °C, hold 0.5 min, 10 °C/min 300 °C, inlet temperature 250 °C. Low resolution mass spectra were recorded on an Agilent Technologies 6120 Quadrupole LC/MS detector in combination with an Agilent Technologies 1260 Infinity HPLC system, equipped with a Zorbax SB-18 column (2.1 x 50mm, 1.8 micron). High resolution mass spectra were recorded on a Waters Synapt HDMS Q-TOF mass spectrometer (ESI ion source,

positive mode, capillary voltage 2.6 kV) using a syringe pump to directly inject the sample dissolved in MeCN. Chiral HPLC analysis was performed on a Shimadzu HPLC system using columns and methods as specified below. Optical rotation values were measured on a Perkin Elmer Polarimeter 341. CD-spectra were recorded in MeOH using a 0.1 cm quartz cuvette on a Jasco J-715 spectropolarimeter (concentrations are given in mg/mL). IR-spectra were recorded neat on a Burker Alpha-P (ATR) instrument. Flash chromatography was performed using Merck silica gel 60 (mesh size 40-63 µm). Petroleum ether had a boiling range of 60-95 °C. Compounds **4**[1] and **5**[2] were prepared according to slightly modified literature protocols as outlined below.

Preparation of 3-bromo-3-(bromomethyl)dihydrofuran-2(3H)-one. α-Methylene-γ-butyrolactone (1.94 g, 19.8 mmol) was dissolved in 1,4-dioxane (100 mL), trimethylphenylammonium tribromide (8.7 g, 23.1 mmol) was added and the suspension was stirred for 16 h at room temperature. Et_2O (200 mL) was added and a white solid formed. Stirring was continued for 20 min, the slurry was filtered through a pad of celite and the yellow filtrate was concentrated under reduced pressure to give crude 3-bromo-3-(bromomethyl)dihydrofuran-2(3H)-one (5.07 g, 19.8 mmol, >99%) as yellow solid with following physical properties: ^{1}H-NMR ($CDCl_3$, 300 MHz): 4.48-4.43 (m, 2H), 4.08 (d, J = 10.5, 1H), 3.91 (d, J = 10.8, 1H), 3.10-2.91 (m, 1H), 2.54 (ddd, J_1 = 1.5, J_2 = 5.1, J_3 = 14.7); ^{13}C-NMR ($CDCl_3$, 75 MHz): 171.7, 65.6, 54.2, 36.4, 33.8.

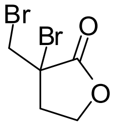

Preparation of 3-(bromomethyl)furan-2(5H)-one (4). 3-bromo-3-(bromomethyl)dihydrofuran-2(3H)-one (5.06 g, 19.8 mmol) was dissolved in acetone (100 mL), Li_2CO_3 (4.92 g, 66.6 mmol) and LiBr (5.88 g, 67.7 mmol) were added and the reaction mixture was refluxed for 17 h. The reaction was cooled to room temperature, poured onto saturated aqueous NH_4Cl solution (200 mL) and extracted with Et_2O (3 x 100 mL). The combined organic phase was dried over Na_2SO_4, filtered and concentrated at 350 mbar. The crude product was chromatographed on silica gel with pentane/Et_2O 3/2 to give bromolactone **4** as a pale yellow oil (1.91g, 10.8 mmol, 55%) with following physical properties: ^{1}H-NMR ($CDCl_3$, 300 MHz): 7.55 (s, 1H), 4.87 (d, J = 1.5, 2H), 4.11 (d, J = 1.2, 2H); ^{13}C-NMR ($CDCl_3$, 75 MHz): 171.5, 149.1, 131.0, 70.2, 20.9; GC-EI-MS (t_{ret} = 6.71 min) m/z (relative intensity [%]) = 178 (11), 176 (11), 131 (0.4), 121 (4), 119 (4), 97 (100); physical data are in accordance with literature.[1]

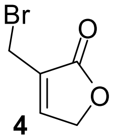

4

Preparation of *O*-benzyl-vanilline (5). Vanilline (2.0 g, 13.1 mmol) and K_2CO_3 (0.88 g, 6.36 mmol) were combined in a 100 mL round bottom flask. Acetone (30 mL) followed by benzyl bromide (1.53 mL, 2.2 g, 12.8 mmol) was added and the mixture was refluxed for 4 h. H_2O (30 mL) was added, the mixture was extracted with EtOAc (2 x 30 mL) and the combined organic phase was dried over Na_2SO_4, filtered and concentrated. The crude product was flash chromatographed on silica gel using petroleum ether/EtOAc 3/1 as eluent to give *O*-benzyl-vanilline (**6a**, 2.52 g, 10.4 mmol, 79%) as white solid with the following physical properties: m.p. 127-130 °C (petroleum ether/EtOAc 3/1), lit.:[2] 128-130 °C (EtOH); ^{1}H-NMR ($CDCl_3$, 300 MHz): 9.84 (s, 1H), 7.46-7.33 (m, 7H), 6.99 (d, J = 8.1, 1H), 5.24 (s, 2H), 3.94 (s, 3H); ^{13}C-NMR ($CDCl_3$, 75 MHz): 190.9, 153.6, 150.1, 136.0, 130.3, 128.7, 128.2, 127.2, 126.6, 112.4, 109.4, 70.9, 56.1; GC-EI-MS (t_{ret} = 15.7 min) m/z (relative intensity [%]) = 242 (7), 91 (100); physical data are in accordance with literature.[2]

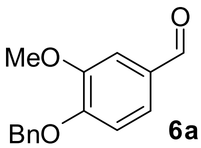

6a

General procedure for the asymmetric allylation of aldehydes. A 50 mL round bottom flask, equipped with a magnetic stir bar, was charged with zinc dust (109 mg, 1.67 mmol), NH_4Cl (130 mg, 2.4 mmol) and (*S*)-TRIP (22 mg, 0.029 mmol). Toluene (12 mL, precooled to 4 °C), $^i Pr_2O$ (3 mL, precooled to 4 °C), the corresponding aldehyde (0.29 mmol) and bromolactone **4** (35 µL, 58 mg, 0.33 mmol) were added, the flask was closed with a stopper and stirred at 4 °C in a fridge for 16 h. The slurry was concentrated under reduced pressure to about 4 mL, which were directly applied to column chromatography on silica gel with toluene/THF 8/1 as eluent except stated otherwise.

Preparation of compound (*rac*)-6 (*syn*:*anti* 21:79). A MG5 vial, equipped with a stir bar, was charged with zinc dust (26 mg, 0.4 mmol), NH_4Cl (34 mg, 0.64 mmol) and *O*-benzyl-vanilline (**5**, 48 mg, 0.2 mmol). DMF (1 mL) and phosphate buffer (1 mL, 100 mM, pH 7.0) were added, followed by bromolactone **4** (25 µL, 42 mg, 0.23 mmol). The mixture was stirred at 720 rpm and 4 °C in a fridge for 24 h. The white slurry was poured on aqueous HCl (10 mL, 0.1 M), the cleared solution was extracted with EtOAc (3 x 20 mL), the combined organic phase was dried over Na_2SO_4, filtered and concentrated. The crude product was chromatographed on silica gel using petroleum ether / EtOAc 1/1 to give a mixture of (*syn/anti-rac*)-**6** as colorless paste. Physical data for the *anti*-isomer was the same as described below. Additional data for the *syn*-isomer: ^{1}H-NMR (acetone-d_6, 300 MHz): 7.51-7.32 (m, 5H), 7.31-6.83 (m, 3H), 6.08 (d, J = 2.4, 1H), 5.26 (d, J = 1.5, 1H), 5.11 (s, 2H),

4.82-4.76 (m, 1H), 4.56 (d, J = 6.0, 1H), 4.47 (dd, J_1 = 4.2, J_2 = 9.3, 1H), 4.31 (dd, J_1 = 8.1, J_2 = 9.0, 1H), 3.85 (s, 3H), 3.55-3.48 (m, 1H).

General procedure for the *anti*-selective preparation racemic reference material for compounds 6-13. A 5 mL Biotage vial, equipped with a magnetic stir bar, was charged with zinc dust (81 mg, 1.24 mmol), NH_4Cl (108 mg, 2.01 mmol) and *O*-benzyl-vanilline (**5**, 150 mg, 0.62 mmol). Dimethoxyethane (3.6 mL) and toluene (3.6 mL) were added, followed by bromolactone **4** (74 µL, 124 mg, 0.7 mmol). The reaction mixture was stirred at 720 rpm and 4 °C for 24 h. The white slurry was poured on aqueous HCl (10 mL, 0.1 M), the cleared solution was extracted with EtOAc (3 x 20 mL), the combined organic phase was dried over Na_2SO_4, filtered and concentrated. The crude product was chromatographed on silica gel using petroleum ether / EtOAc 1/1 to give *anti*-(*rac*)-**6** as a colorless solid (*anti*:*syn* = >95:5). The physical data were identical for enantiopure (*R*,*S*)-**6** as described below.

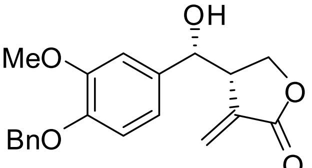

ent-**6**

(*S*)-4-{(*R*)-[4-(Benzyloxy)-3-methoxyphenyl](hydroxy)methyl}-3-methylenedihydrofuran-2(3H)-one (ent-6). The general procedure for the asymmetric allylation of aldehydes was upscaled 1.41 x (100 mg aldehyde, 0.41 mmol) and all reagents were kept in the same ratio to yield (*S*,*R*)-**6** as white solid (98 mg, 0.29 mmol, 71%); $[\alpha]_D^{20}$ -3.87 (c 1.0, $CHCl_3$); chiral HPLC analysis of acetylated sample {Daicel Chiralpak AD, *n*-heptane/2-propanol 80/20, 0.4 mL/min, 18 °C, UV 215 nm, t_{ret}(*syn*-isomer 1) = 44.9 min, t_{ret}(*syn*-isomer 2), t_{ret}(*syn*-isomer 1) = 49.1 min, t_{ret}(ent-**6**) = 51.9, t_{ret}(**6**) = 70.4 min}: t_{ret}(major isomer) = 53.3 min, ee 90%; all other physical data were identical to that of **6**.

(*S*)-4-[(*R*)-Hydroxy(phenyl)methyl]-3-methylenedihydrofuran-2(3H)-one (7). White solid

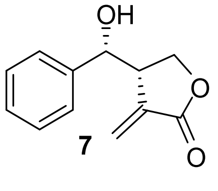

7

(52 mg, 0.25 mmol, 88%); m.p. 121-124 °C (toluene/THF 8/1), lit.:[1] 120-125 °C; $[\alpha]_D^{20}$ -14.5 (c 1.0, $CHCl_3$); ^{1}H-NMR ($CDCl_3$, 300 MHz): 7.43-7.33 (m, 5H), 6.36 (d, J = 2.1, 1H), 5.79 (d, J = 1.5, 1H), 4.72 (d, J = 7.5, 1H), 4.17 (dd, J_1 = 8.4, J_2 = 9.6, 1H), 4.07 (dd, J_1 = 4.2, J_2 = 9.6, 1H), 3.46-3.38 (m, 1H); ^{13}C-NMR ($CDCl_3$, 75 MHz): 170.8, 140.7, 135.0, 128.9, 128.7, 126.6, 125.5, 75.6, 67.6, 45.5; Chiral HPLC analysis [Daicel Chiralpak AD, *n*-heptane/2-propanol 85/15, 0.7 mL/min, 18 °C, UV 215 nm, t_{ret}(**7**) = 13.6 min, t_{ret}(ent-**7**) = 15.2 min]: t_{ret}(major isomer) = 13.7 min, ee 94%; LRMS (ESI): 227.1 (100, M+Na^+); HRMS (ESI): $[MH - H_2O]^+$ found

187.0754, $C_{12}H_{11}O_2^+$ requires 187.0759. Analytical data are in agreement with reported properties.[1]

(*S*)-4-[(*R*)-Hydroxy(4-methoxyphenyl)methyl]-3-methylenedihydrofuran-2(3H)-one (8).

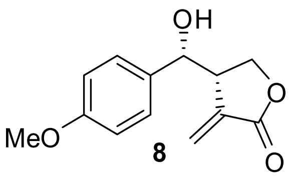

8

Colorless oil (54 mg, 0.23 mmol, 79%); $[\alpha]_D^{20}$ -9.2 (c 1.0, $CHCl_3$); ^{1}H-NMR ($CDCl_3$, 300 MHz): 7.27 (d, J = 8.7, 2H), 6.92 (d, J = 8.7, 2H), 6.37 (d, J = 2.1, 1H), 5.87 (d, J = 1.2, 1H), 4.65 (d, J = 8.1, 1H), 4.15 (dd, J_1 = 8.4, J_2 = 9.6, 1H), 4.01 (dd, J_1 = 4.5, J_2 = 9.6, 1H), 3.83 (s, 3H), 3.45-3.36 (m, 1H); ^{13}C-NMR ($CDCl_3$, 75 MHz): 170.8, 159.8, 135.2, 132.8, 127.8, 125.4, 114.2, 75.3, 67.5, 55.4, 45.5; Chiral HPLC analysis [Daicel Chiralpak AD, *n*-heptane/2-propanol 90/10, 0.7 mL/min, 18 °C, UV 215 nm, t_{ret}(**8**) = 31.2 min, t_{ret}(ent-**8**) = 35.4 min]: t_{ret}(major isomer) = 31.4 min, ee 96%; LRMS (ESI): 257.1 (100, M+Na$^+$); HRMS (ESI): $[MH - H_2O]^+$ found 217.0860, $C_{13}H_{13}O_3^+$ requires 217.0865. Analytical data are in agreement with reported properties.[3]

(*S*)-4-[(*R*)-Furan-2-yl(hydroxy)methyl]-3-methylenedihydrofuran-2(3H)-one (9). Yellow

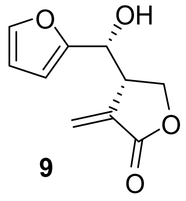

9

oil (51 mg, 0.26 mmol, 91%, *syn*:*anti* = 14:86); $[\alpha]_D^{20}$ -32.9 (c 1.0, $CHCl_3$); ^{1}H-NMR ($CDCl_3$, 300 MHz): 7.42-7.41 (m, 1H), 6.37 (m, 2H), 6.34 (d, J = 3, 1H), 5.76 (d, J = 2.1, 1H), 4.77 (d, J = 7.5, 1H), 4.35 (dd, J_1 = 8.4, J_2 = 9.3, 1H), 4.18 (dd, J_1 = 4.5, J_2 = 9.6, 1H), 3.64-3.57 (m, 1H); minor isomer: 7.43 (m, 1H), 6.37 (m, 2H), 6.27 (d, J = 2.7, 1H), 5.22 (d, J = 2.1, 1H), 4.73 (m, 1H), 4.55 (dd, J_1 = 4.2, J_2 = 9.6, 1H), 4.46 (dd, J_1 = 8.1, J_2 = 9.6, 1H), 3.64-3.57 (m, 1H); ^{13}C-NMR ($CDCl_3$, 75 MHz): 170.6, 153.2, 142.8, 134.2, 125.7, 110.5, 108.2, 69.2, 67.5, 43.3; Chiral HPLC analysis [Daicel Chiracel OJ, *n*-heptane/2-propanol 93/7, 1.0 mL/min, 18 °C, UV 215 nm, inseparable *syn*:*anti* mixture; LRMS (ESI): 217.1 (100, M+Na$^+$); HRMS (ESI): $[MH - H_2O]^+$ found 177.0547, $C_{10}H_9O_3^+$ requires 177.0552. Analytical data are in agreement with reported properties.[3]

(*S*)-4-[(*R*)-(3-Chlorophenyl)(hydroxy)methyl]-3-methylenedihydrofuran-2(3H)-one (10).

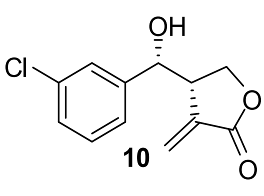

10

Yellow oil [36 mg, 0.15 mmol, 52%, inseparable minor impurity of (*S*)-TRIP catalyst (5 mol-%, NMR shifts between 1-2 ppm)]; $[\alpha]_D^{20}$ -13.8 (c 1.0, $CHCl_3$); ^{1}H-NMR ($CDCl_3$, 300 MHz): 7.36-7.20 (m, 4H), 6.33 (d, J = 2.1, 1H), 5.69 (d, J = 1.5, 1H), 4.72 (d, J = 7.2, 1H), 4.22 (dd, J_1 = 8.1, J_2 = 9.6, 1H), 4.11 (dd, J_1 = 4.2, J_2 = 9.6, 1H), 3.41-3.34 (m, 1H), 2.95 (bs, 1H);

^{13}C-NMR ($CDCl_3$, 75 MHz): 170.9, 142.8, 134.8, 134.4, 130.1, 128.7, 126.7, 125.8, 124.8, 74.8, 67.7, 45.4; Chiral HPLC analysis [Daicel Chiralpak AD, *n*-heptane/2-propanol 90/10, 0.7 mL/min, 18 °C, UV 215 nm, t_{ret}(**10**) = 21.7 min, t_{ret}(ent-**10**) = 23.7 min]: t_{ret}(major isomer) = 19.9 min, ee 96%; LRMS (ESI): 261.1 (100, M+Na$^+$); HRMS (ESI): [MH - H_2O]$^+$ found 221.0373, $C_{12}H_{10}O_2Cl^+$ requires 221.0369. Analytical data are in agreement with reported properties.[3]

(*S*)-4-[(*R*)-(4-Fluorophenyl)(hydroxy)methyl]-3-methylenedihydrofuran-2(3H)-one (11).

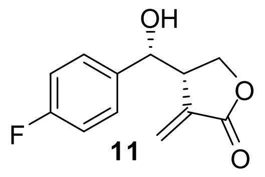

Colorless oil [52 mg, 23 mmol, 81%, inseparable minor impurity of (*S*)-TRIP catalyst (5 mol-%, NMR shifts between 1-2 ppm)], $[\alpha]_D^{20}$ -10.6 (c 1.0, $CHCl_3$); IR (neat, cm^{-1}): 3466s, 1741s, 1502s, 1218s; ^{1}H-NMR ($CDCl_3$, 300 MHz): 7.39-7.32 (m, 2H), 7.08 (t, *J* = 8.4, 2H), 6.36 (d, *J* = 2.1, 1H), 5.76 (d, *J* = 1.8, 1H), 4.73 (d, *J* = 7.5, 1H), 4.19 (dd, J_1 = 8.1, J_2 = 9.6, 1H), 4.07 (dd, J_1 = 4.2, J_2 = 9.6, 1H), 3.42-3.34 (m, 1H), 2.66 (bs, 1H); ^{13}C-NMR ($CDCl_3$, 75 MHz): 170.7, 162.7 (d, *J* = 246), 136.5 (d, *J* = 3.15), 134.8, 128.3 (d, *J* = 33.7), 125.9, 115.8 (d, *J* = 85.5), 74.9, 67.6, 45.6; Chiral HPLC analysis of acetylated sample [Daicel Chiralpak AD, *n*-heptane/2-propanol 85/15, 0.7 mL/min, 18 °C, UV 215 nm, t_{ret}(**11**) = 19.5 min, t_{ret}(ent-**11**) = 22.1 min]: t_{ret}(major isomer) = 19.4 min, ee 97%; LRMS (ESI): 245.1 (100, M+Na$^+$); HRMS (ESI): [MH - H_2O]$^+$ found 205.0655, $C_{12}H_{10}O_2F^+$ requires 205.0665.

(*S*)-4-[(*R*)-(4-*tert*-Butylphenyl)(hydroxy)methyl]-3-methylenedihydrofuran-2(3H)-one

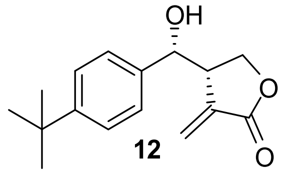

(12). Pale yellow solid (69 mg, 0.26 mmol, 91%, column chromatography was performed using toluene/acetone 20/1 as eluent); m.p. 78-80 °C (toluene/acetone 20/1); $[\alpha]_D^{20}$ -32.8 (c 1.0, $CHCl_3$); ^{1}H-NMR ($CDCl_3$, 300 MHz): 7.41 (d, *J* = 8.4, 2H), 7.28 (d, *J* = 8.4, 2H), 6.36 (d, *J* = 2.4, 1H), 5.86 (d, *J* = 1.5, 1H), 4.66 (d, *J* = 8.1, 1H), 4.16 (dd, J_1 = 8.4, J_2 = 9.6, 1H), 4.03 (dd, J_1 = 4.5, J_2 = 9.6, 1H), 3.46-3.38 (m, 1H), 2.52 (bs, 1H), 1.34 (s, 9H); ^{13}C-NMR ($CDCl_3$, 75 MHz): 170.9, 151.7, 137.7, 135.2, 126.4, 125.8, 125.5, 75.4, 67.7, 45.4, 34.7, 31.3; Chiral HPLC analysis [Daicel Chiralpak AD, *n*-heptane/2-propanol 93/7, 1.0 mL/min, 18 °C, UV 215 nm, t_{ret}(**12**) = 15.7 min, t_{ret}(ent-**12**) = 17.5 min]: t_{ret}(major isomer) = 15.5 min, ee >99%; LRMS (ESI): 283.1 (100, M+Na$^+$); HRMS (ESI): [M + Na]$^+$ found 283.1313, $C_{16}H_{20}O_3Na^+$ requires 283.1310. Analytical data are in agreement with reported properties.[3]

(*S*)-4-[(*R*)-Hydroxy(naphthalen-2-yl)methyl]-3-methylenedihydrofuran-2(3H)-one (13).

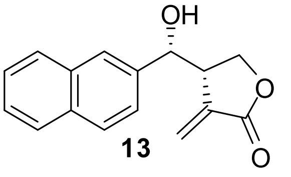
13

Pale yellow solid (58 mg, 0.23 mmol, 80%, column chromatography was performed using toluene/acetone 20/1 as eluent); m.p. 150-152 °C (toluene/acetone 20/1), lit.:[1] 154-158 °C (petroleum ether/EtOAc 1/1); $[\alpha]_D^{20}$ -20.9 (c 1.0, $CHCl_3$); ^{1}H-NMR ($CDCl_3$, 300 MHz): 7.88-7.82 (m, 3H), 7.75 (s, 1H), 7.53 (dt, J_1 = 3.0, J_2 = 9.6, 3H), 6.33 (d, J = 2.1, 1H), 5.74 (d, J = 1.5, 1H), 4.81 (d, J = 7.5, 1H), 4.16-4.05 (m, 2H), 3.50-3.42 (m, 1H), 2.96 (bs, 1H); ^{13}C-NMR ($CDCl_3$, 75 MHz): 171.0, 138.1, 134.8, 133.3, 133.0, 128.9, 128.1, 127.8, 126.6, 126.5, 125.9, 125.6, 124.0, 75.8, 67.8, 45.3; Chiral HPLC analysis [Daicel Chiralpak AD, *n*-heptane/2-propanol 90/10, 0.7 mL/min, 18 °C, UV 215 nm, t_{ret}(**13**) = 30.8 min, t_{ret}(ent-**13**) = 33.9 min]: t_{ret}(major isomer) = 31.2 min, ee 97%; LRMS (ESI): 277.1 (100, M+Na$^+$); HRMS (ESI): [MH - $H_2O]^+$ found 237.0927, $C_{16}H_{13}O_2^+$ requires 237.0916. Analytical data are in agreement with reported properties.[3]

Preparation of (*R*)-4-{(*S*)-[4-(benzyloxy)-3-methoxyphenyl](hydroxy)methyl}-3-methylenedihydrofuran-2(3H)-one (6).

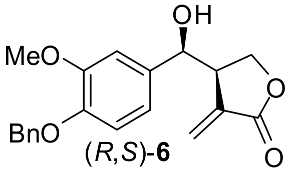
(*R*,*S*)-**6**

A 50 mL round bottom flask, equipped with a magnetic stir bar, was charged with zinc dust (150 mg, 2.3 mmol), NH_4Cl (170 mg, 3.2 mmol) and (*R*)-TRIP (60 mg, 0.08 mmol). Toluene (16 mL, precooled to 4 °C), ^{i}Pr$_2$O (4 mL, precooled to 4 °C), 3-methoxy-4-benzyloxybenzaldehyde (100 mg, 0.41 mmol) and bromolactone **4** (86 µL, 144 mg, 0.81 mmol) were added, the flask was closed with a stopper and stirred at 4 °C in a fridge for 16 h. The slurry was concentrated under reduced pressure to about 4 mL, which were directly applied to column chromatography on silica gel with toluene/THF 8/1 as eluent to give compound **6** as white solid (98 mg, 0.29 mmol, 71%) with following physical properties: m.p. 129-132 °C (toluene/THF 8/1); $[\alpha]_D^{20}$ +5.64 (c 1.0, $CHCl_3$); IR (neat, cm^{-1}): 3520m, 1750s, 1592s, 1252s, 1225s; ^{1}H-NMR (acetone-d$_6$, 300 MHz): 7.52-7.51 (m, 2H), 7.43-7.31 (m, 3H), 7.09 (d, J = 1.8, 1H), 7.00 (s, 1H), 6.93 (dd, J_1 = 1.8, J_2 = 8.1, 1H), 6.15 (dd, J_1 = 1.5, J_2 = 2.4, 1H), 5.61 (dd, J_1 = 1.2, J_2 = 1.8, 1H), 5.12 (s, 2H), 4.81 (dd, J_1 = 4.5, J_2 = 6.6, 1H), 4.72 (d, J = 4.2, 1H), 4.23 (dd, J_1 = 8.4, J_2 = 9.0, 1H), 4.14 (dd, J_1 = 4.5, J_2 = 9.3, 1H), 3.84 (s, 3H), 3.55-3.47 (m, 1H); ^{13}C-NMR (acetone-d$_6$, 75 MHz): 170.2, 149.9, 17.9, 137.7, 136.3, 135.5, 128.3, 127.7, 127.6, 122.8, 118.8, 113.8, 110.7, 74.8, 70.6, 67.6, 55.3, 45.5; chiral HPLC analysis of acetylated sample {Daicel Chiralpak AD, *n*-heptane/2-propanol 80/20, 0.4 mL/min, 18 °C, UV 215 nm, t_{ret}(syn-isomer 1) = 44.9 min, t_{ret}(syn-isomer 2) = 49.1 min, t_{ret}(ent-**6**) = 51.9, t_{ret}(**6**) = 70.4 min}: t_{ret}(major

isomer) = 72.4 min, ee 98%; LRMS (ESI): 363.1 (M+Na^+); HRMS (ESI): $[M + Na]^+$ found 363.1219, $C_{20}H_{20}O_5Na^+$ requires 363.1208.

Preparation of (3*R*,4*R*)-4-{(*S*)-[4-(benzyloxy)-3-methoxyphenyl](hydroxy)methyl}-3-(4-hydroxy-3-methoxybenzyl)dihydrofuran-2(3H)-one (14).

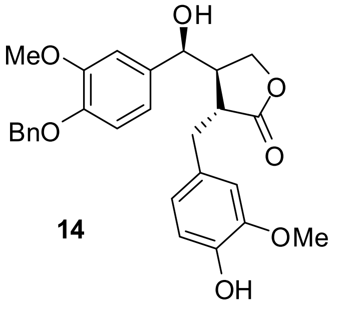

Compound **6** (82 mg, 0.24 mmol), 4-hydroxy-3-methoxyphenylboronic acid pinacol ester (101 mg, 0.40 mmol) and $[RhCl(cod)_2]_2$ (4 mg, 0.008 mmol) were combined in a 5 mL Biotage vial and dissolved in 1,4-dioxane (600 µL) and $H_2O_{dist.}$ (198 µL). Et_3N (33 µL, 24 mg, 0.24 mmol) was added and the vial was capped, crimped with an alumina ring and stirred (720 rpm) in a preheated oil bath (90 °C bath temperature). After 2h the reaction mixture was cooled to room temperature and directly applied to flash chromatography on silica gel using petroleum ether/EtOAc 3/2 as eluent to give compound **14** (84 mg, 0.18 mmol, 75%) as white gum with following physical properties: $[\alpha]_D^{20}$ -8.43 (c 1.0, $CHCl_3$); IR (neat, cm^{-1}): 3270br, 2963m, 1752s, 1511s, 1259s, 1122s; ^{1}H-NMR (acetone-d_6, 300 MHz): 7.52-7.50 (m, 2H), 7.43-7.30 (m, 4H), 6.98-6.95 (m, 2H), 6.81 (dd, J_1 = 1.8, J_2 = 8.4, 1H), 6.69 (d, J = 8.1, 1H), 6.67 (d, J = 1.8, 1H), 6.55 (dd, J_1 = 1.8, J_2 = 8.1, 1H), 5.12 (s, 2H), 4.79 (t, J = 3.9, 1H), 4.75 (d, J = 3.9, 1H), 4.10-3.98 (m, 2H), 3.81 (s, 3H), 3.77 (s, 3H), 2.96-2.89 (m, 1H) 2.78-2.65 (m, 2H); ^{13}C-NMR (acetone-d_6, 75 MHz): 178.6, 149.9, 147.7, 147.2, 145.2, 137.8, 136.2, 129.3, 128.3, 127.7, 127.6, 122.3, 118.1, 114.6, 113.8, 110.2, 73.5, 70.6, 68.3, 55.3, 45.1, 42.8, 34.7; chiral HPLC analysis of acetylated sample {Daicel Chiralpak AD, *n*-heptane/2-propanol 65/35, 0.4 mL/min, 18 °C, UV 215 nm, t_{ret}(ent-**14**) = 39.9, t_{ret}(**14**) = 48.3 min}: t_{ret}(major isomer) = 48.6 min, ee 98%; LRMS (ESI): 487.3 (M+Na^+); HRMS (ESI): $[M + Na]^+$ found 487.1748, $C_{27}H_{28}O_7Na^+$ requires 487.1733.

Preparation of (7'*S*)-hydroxymatairesinol [(7'*S*,8*R*,8'*R*)-4,4',7'-trihydroxy-3,3'-dimethoxylignano-9,9'-lactone, (1)].

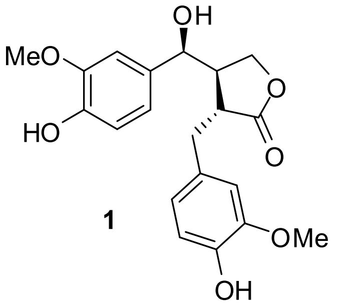

Compound (*R*,*S*,*R*)-**14** (74 mg, 0.16 mmol) was dissolved in EtOAc (5 mL) and Pd/C (10 wt-%, 21 mg) was added. The flask was evacuated and vented with hydrogen five times and then stirred for 16 h under 1 atm. of hydrogen. The reaction mixture was filtered through celite, the residue was washed with additional EtOAc (30 mL) and the combined filtrate was concentrated under reduced pressure to give (7'*S*)-hydroxymatairesinol [(7'*S*,8*R*,8'*R*)-**1**, 51 mg, 86%] as a white gum with following physical properties: $[\alpha]_D^{20}$ -8.27

(c 1.0, THF), lit.:[4] -8.2 (c 1.64, THF); ^{1}H-NMR ($CDCl_3$, 300 MHz): 6.86 (d, J = 8.1, 1H), 6.78 (d, J = 8.4, 1H), 6.72 (dd, J_1 = 1.8, J_2 = 8.1, 1H), 6.67 (d, J = 1.8, 1H), 6.61-6.58 (m, 2H), 5.74 (bs, 1H), 5.63 (bs, 1H), 4.64 (d, J = 6.6, 1H), 4.02-3.91 (m, 2H), 3.84 (s, 3H), 3.81 (s, 3H), 3.01-2.88 (m, 3H), 2.65-2.56 (m, 1H); ^{13}C-NMR ($CDCl_3$, 75 MHz): 179.5, 146.8, 146.6, 145.5, 144.4, 133.5, 129.5, 122.5, 118.7, 114.4, 114.0, 111.9, 108.2, 75.2, 68.6, 55.9, 45.1, 43.6, 35.1; chiral HPLC analysis of acetylated sample {Daicel Chiralpak AD, *n*-heptane/2-propanol 60/40, 0.4 mL/min, 18 °C, UV 215 nm, t_{ret}[(7'*S*,8*R*,8'*S*)-**14**] = 17.7, t_{ret}[(7'*R*,8*S*,8'*R*)-**14**] = 31.8 min}: t_{ret}(major isomer) = 31.8 min, ee 98%; LRMS (ESI): 397.2 ($M+Na^+$); HRMS (ESI): $[M + Na]^+$ found 397.1263, $C_{20}H_{22}O_7Na^+$ requires 397.1263. Analytical data are in agreement with reported properties.[1]

Derivatisation of compounds 6, 11, 14 and 1 for determination of enantiomeric purity. A small sample of analyte (ca. 4 mg) was dissolved in EtOAc (1 mL) in a 2 mL Eppendorf vial. 4-(Dimethylamino)pyridine (2 mg), Et_3N (150 µL) and acetic anhydride (100 µL) were added and the vial was shaken at 60 °C for 3 h. The reaction was quenched by adding phosphate buffer (500 µL, 100 mM, pH 7.0), the organic phase was separated, dried over Na_2SO_4, filtered and the solvent was removed under a positive stream of air. The remaining residue was dissolved in 2-propanol and subjected to HPLC analysis.

Determination of absolute configuration.

- The absolute configuration of compound **6** was determined via asymmetric total synthesis of (*S*)-(-)-hydroxymatairesinol (**1**) and comparison of the optical rotation with literature data.[4]
- The absolute configurations of compounds **7-13** were determined via CD-spectroscopy (*vide infra*) and correlated to that of compound **6**; in addition, the elution order of enantiomers of compounds **6-13** on a chiral stationary phase (Daicel Chiralpak AD) was consistent, i.e. the (*S*)-alcohol eluted first.

^{1}H- and ^{13}C-NMR spectra

3-Bromo-3-(bromomethyl)dihydrofuran-2(3H)-one (crude).

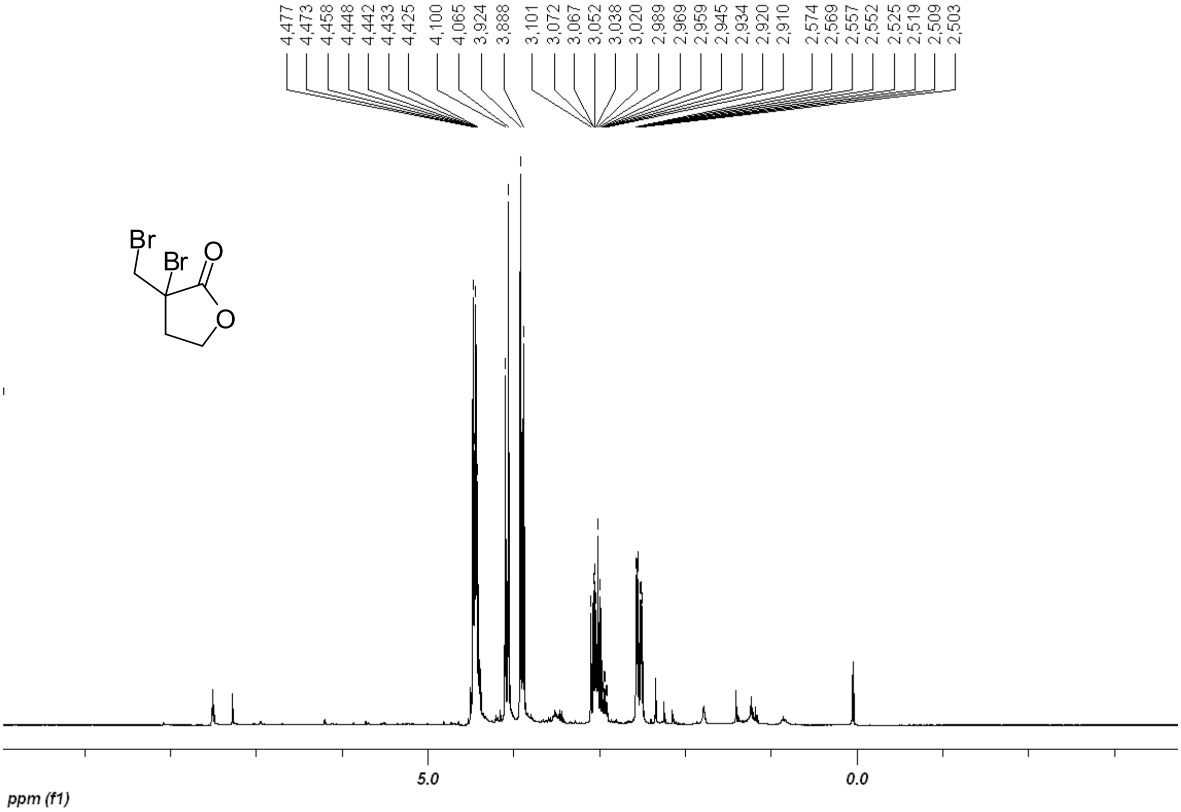

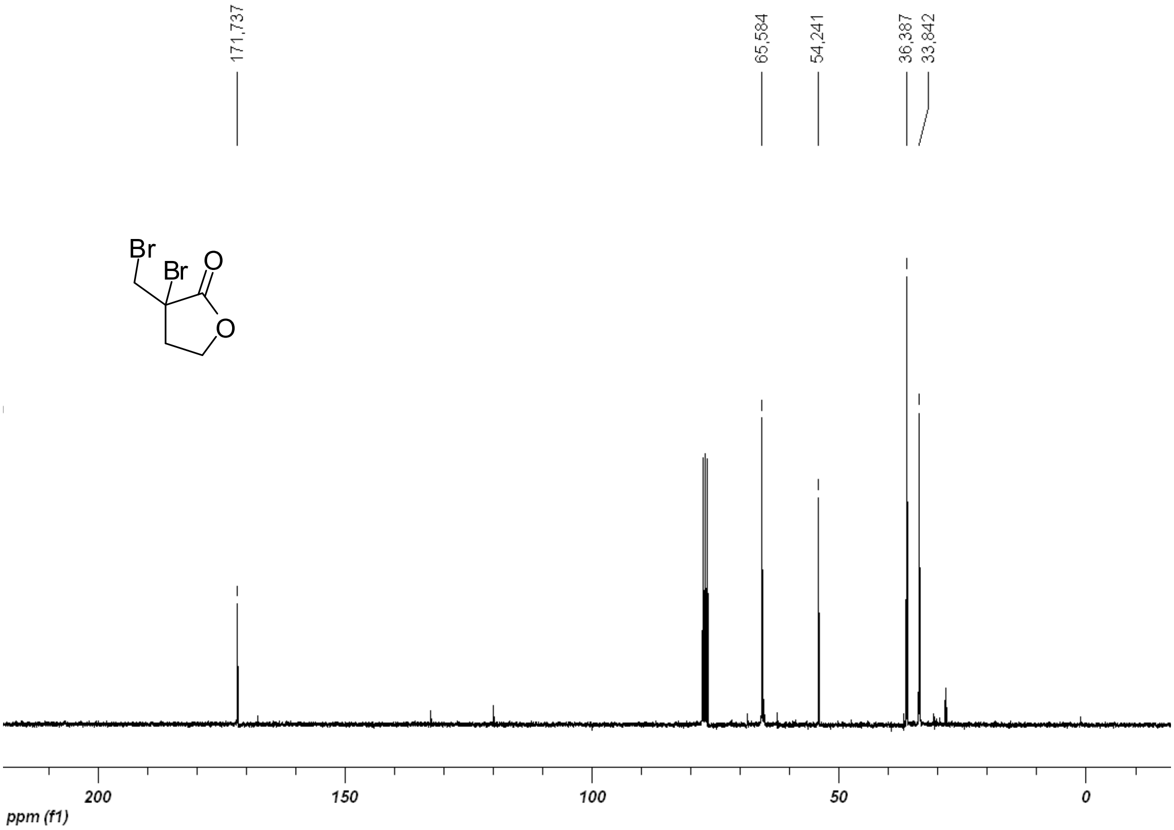

3-(Bromomethyl)furan-2(5H)-one (4).

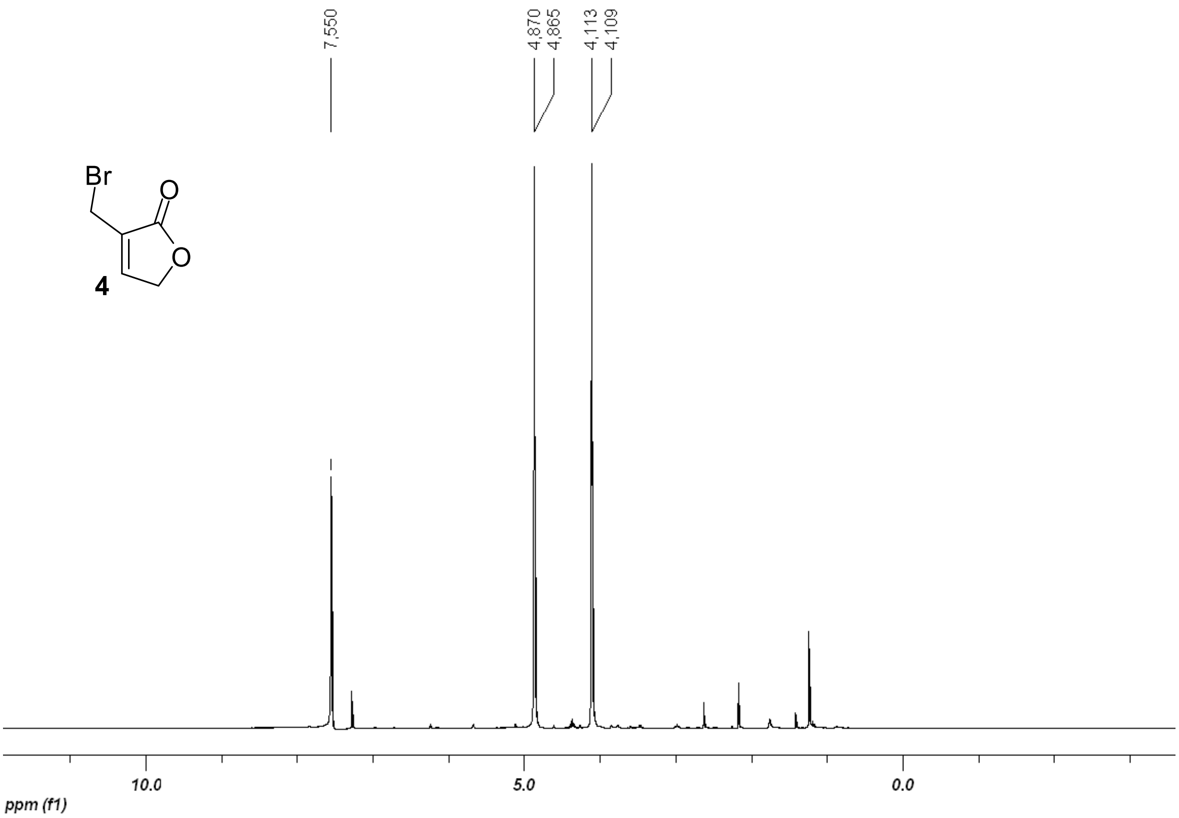

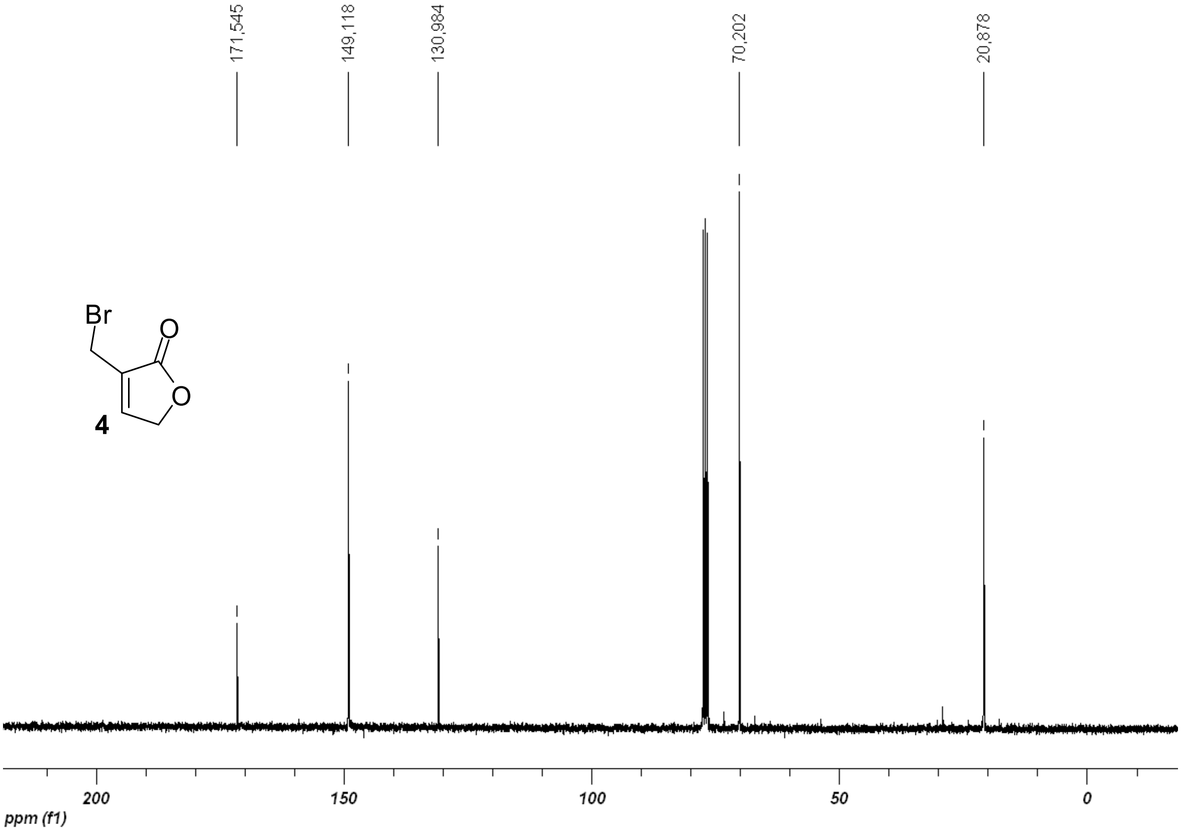

***O*-Benzyl-vanilline (5).**

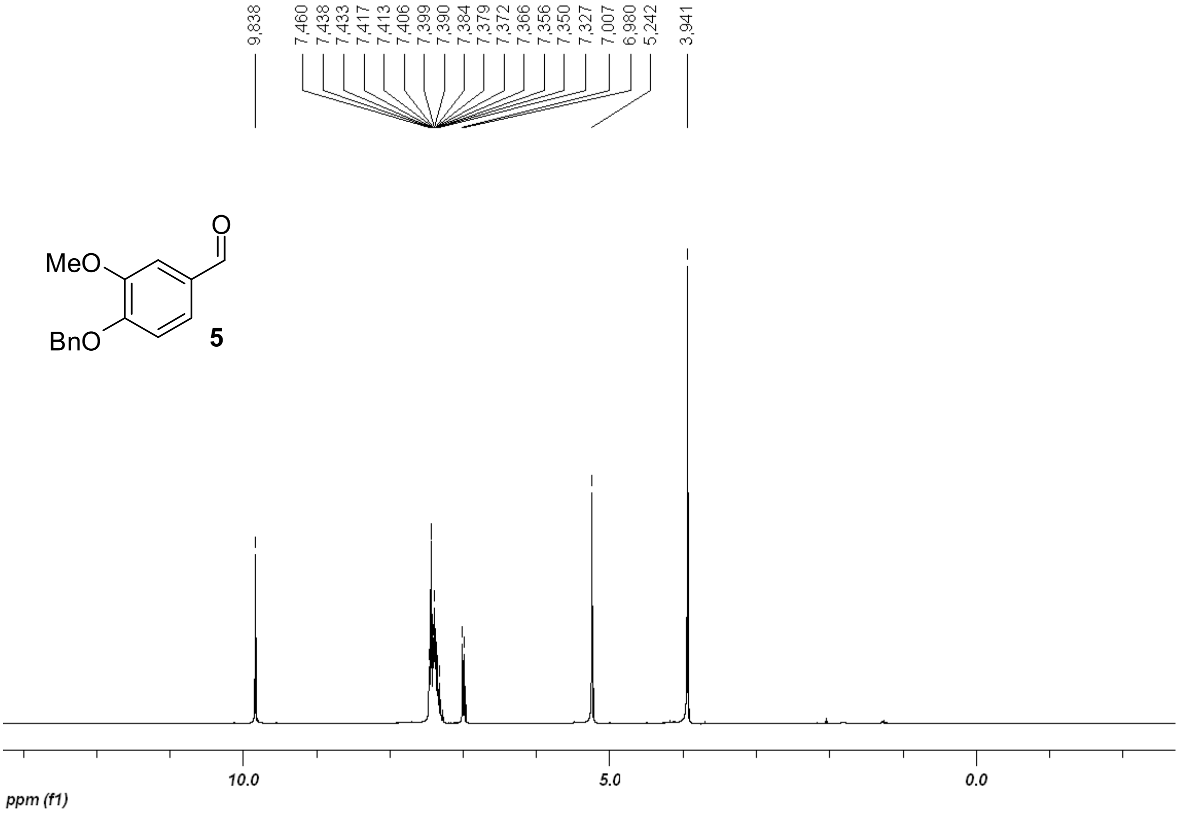

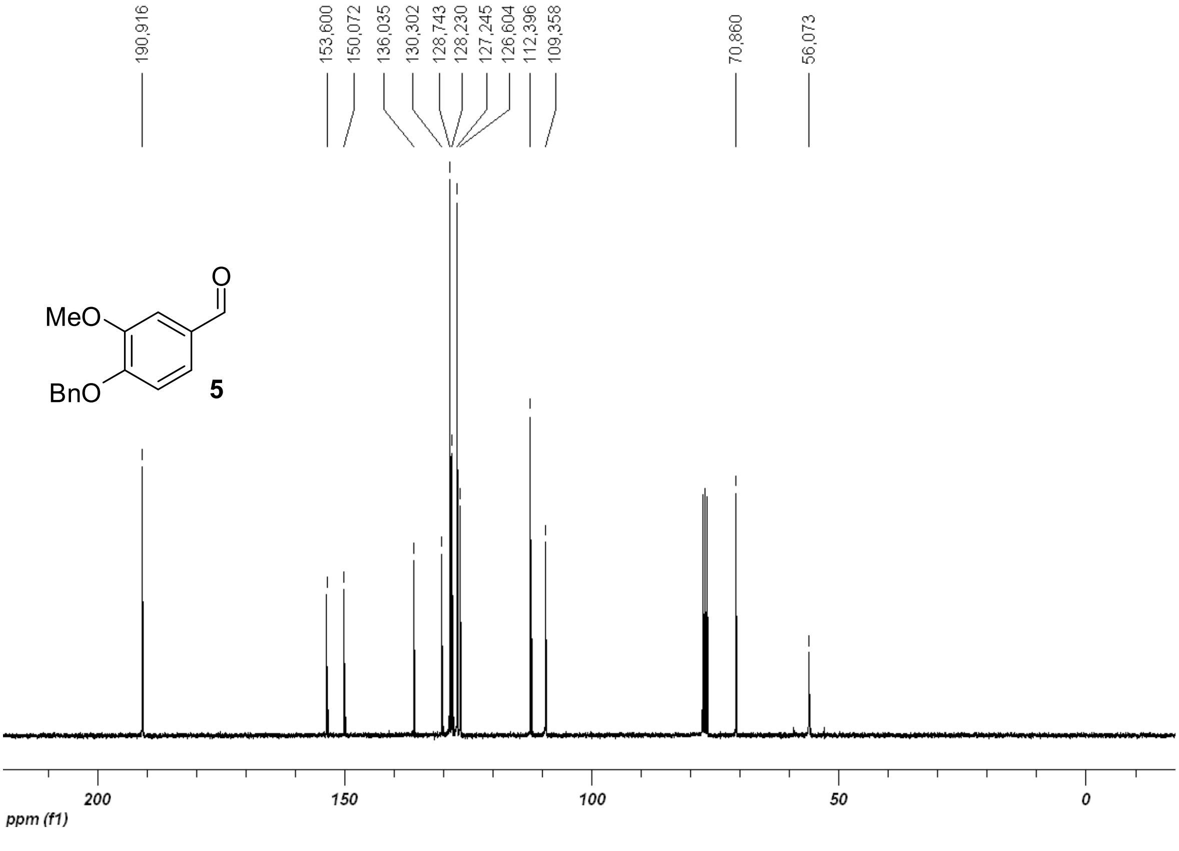

(*rac*)-4-{[4-(Benzyloxy)-3-methoxyphenyl](hydroxy)methyl}-3-methylenedihydrofuran-2(3H)-one [(*rac*)-6, *syn:anti* 21:79].

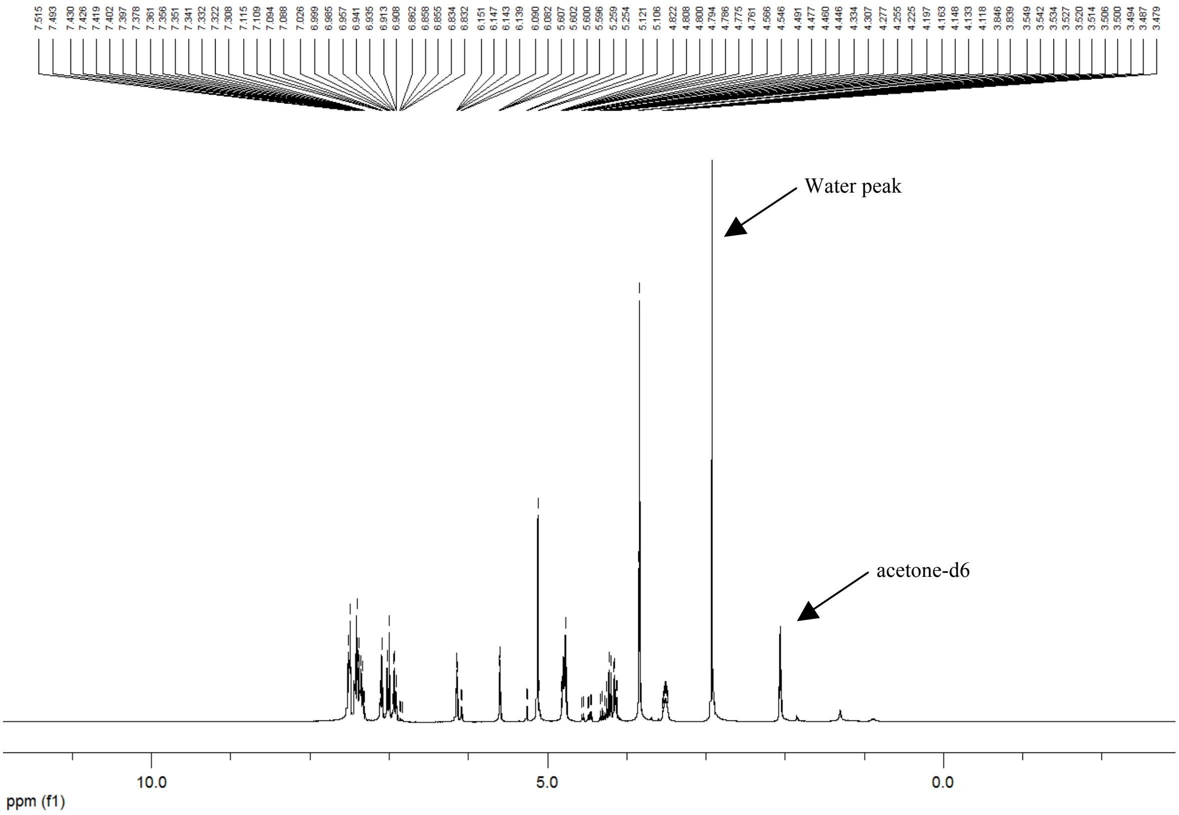

(*S*)-4-[(*R*)-Hydroxy(phenyl)methyl]-3-methylenedihydrofuran-2(3H)-one (7).

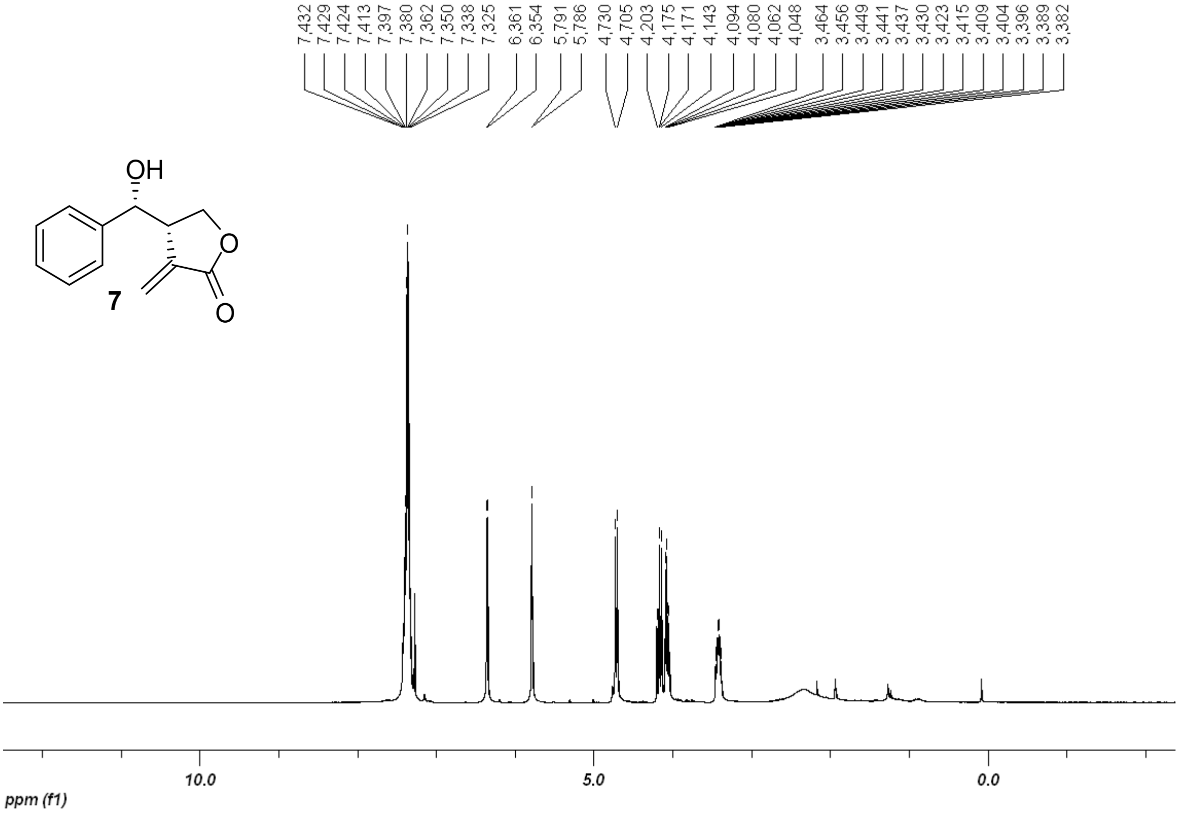

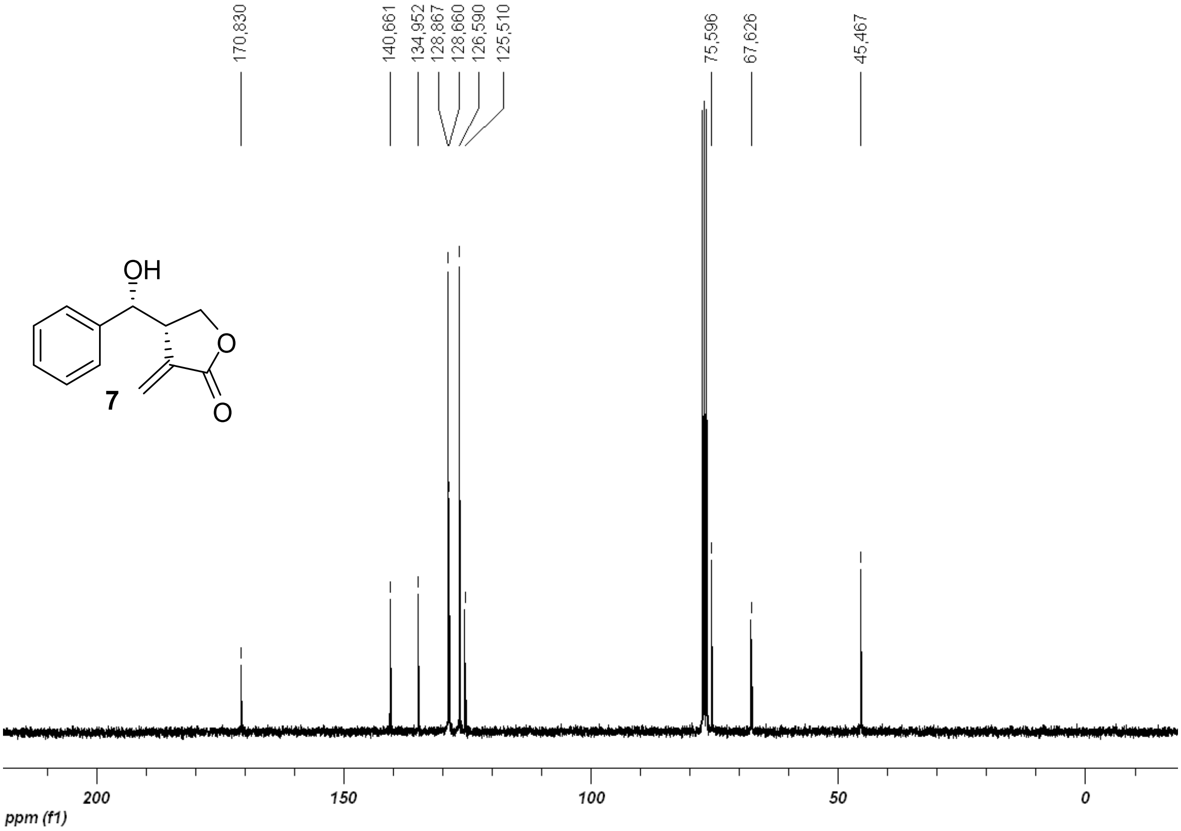

(*S*)-4-[(*R*)-Hydroxy(4-methoxyphenyl)methyl]-3-methylenedihydrofuran-2(3H)-one (8).

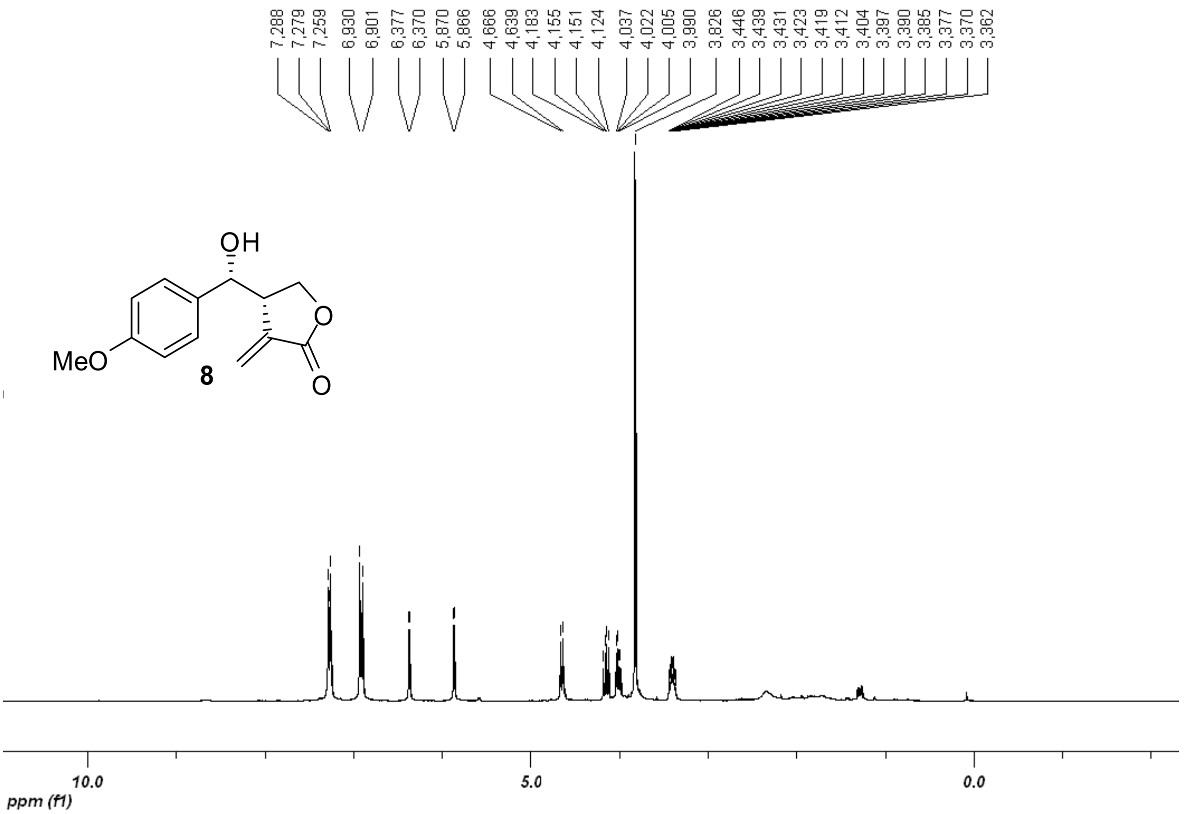

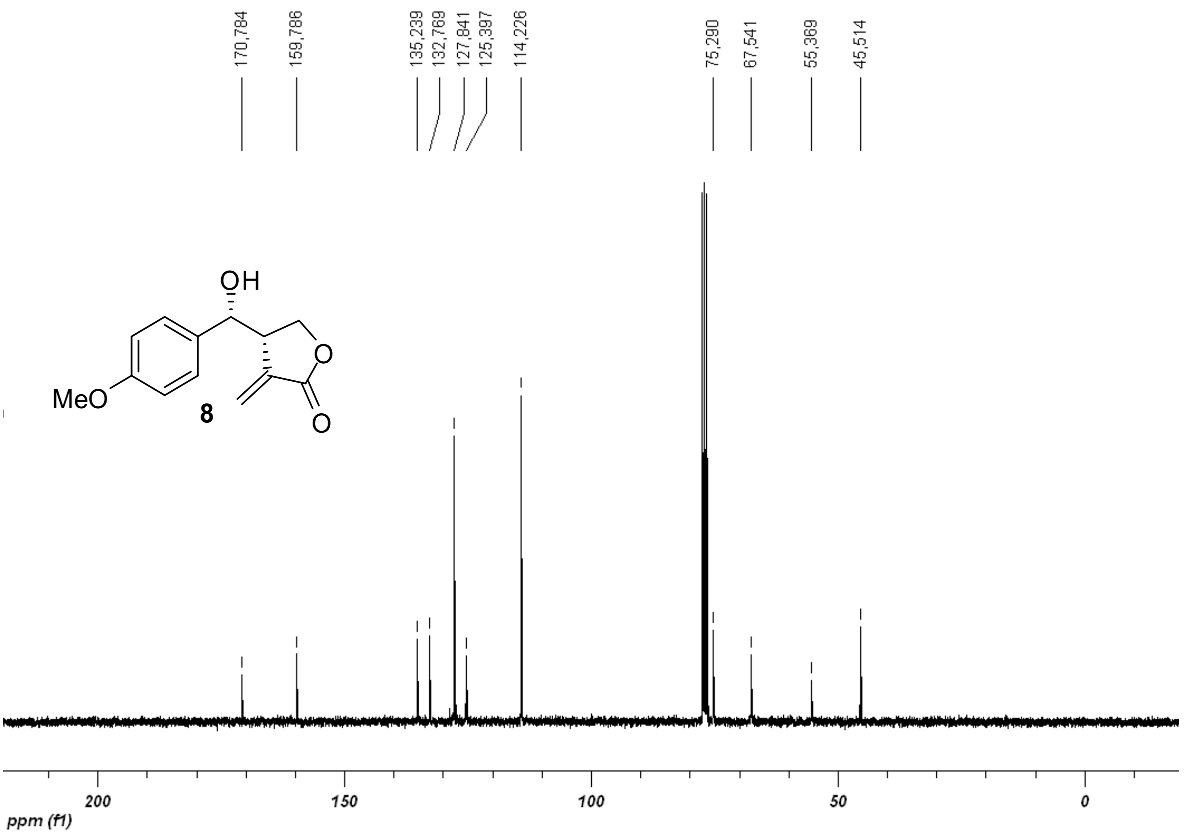

(*S*)-4-[(*R*)-Furan-2-yl(hydroxy)methyl]-3-methylenedihydrofuran-2(3H)-one (9).

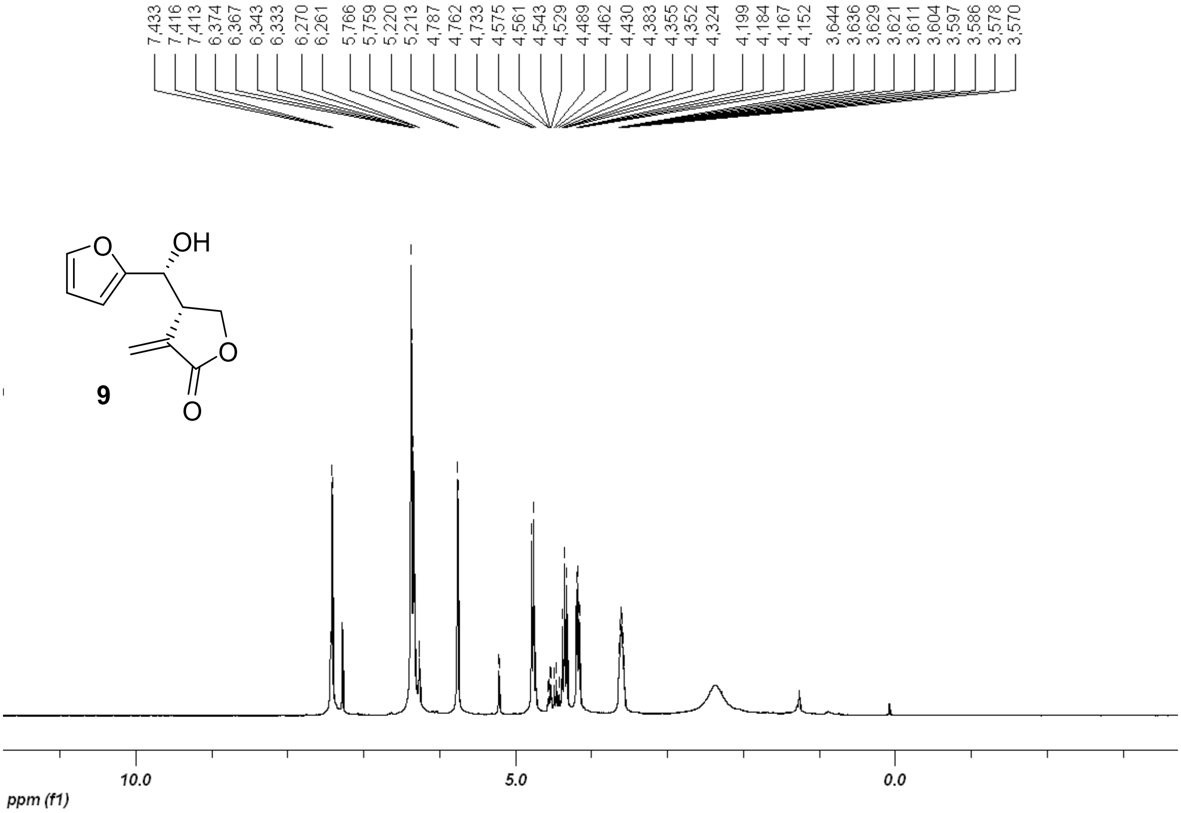

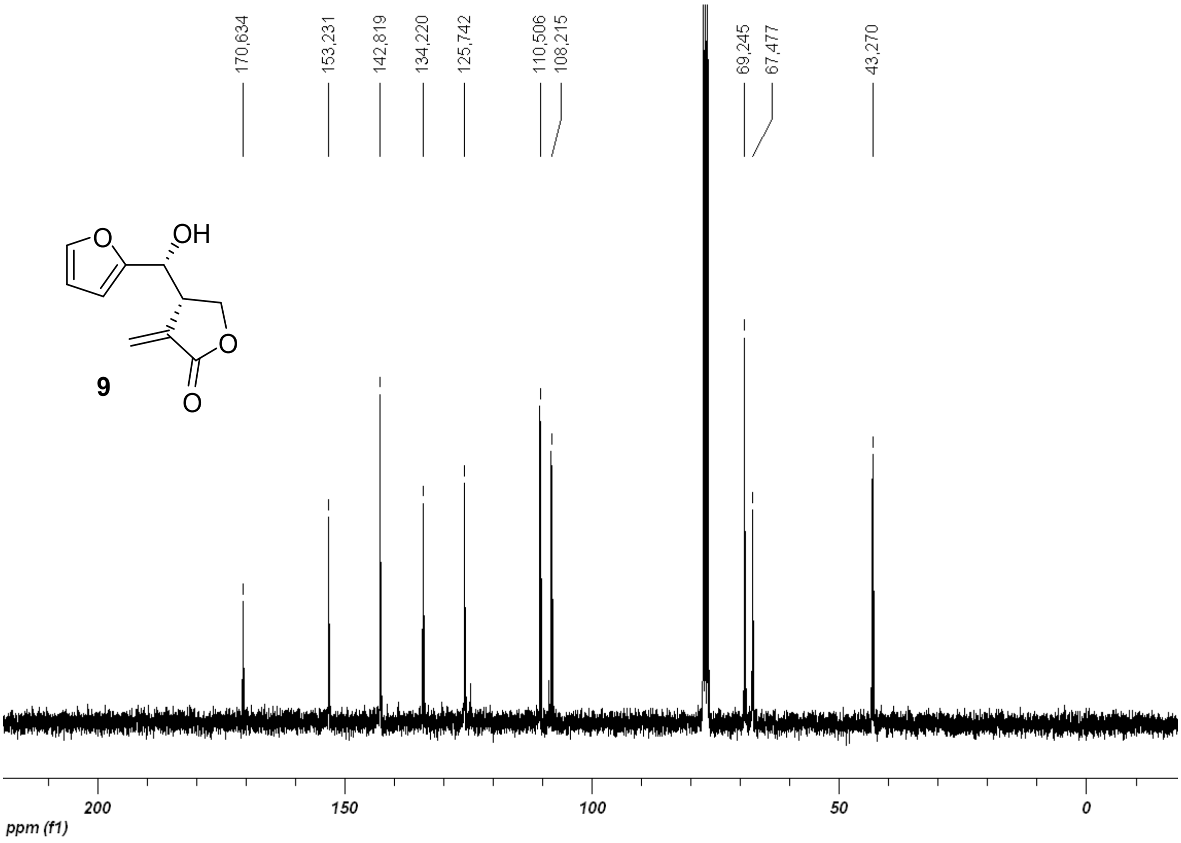

(*S*)-4-[(*R*)-3-(Chlorophenyl)(hydroxy)methyl]-3-methylenedihydrofuran-2(3H)-one (10).

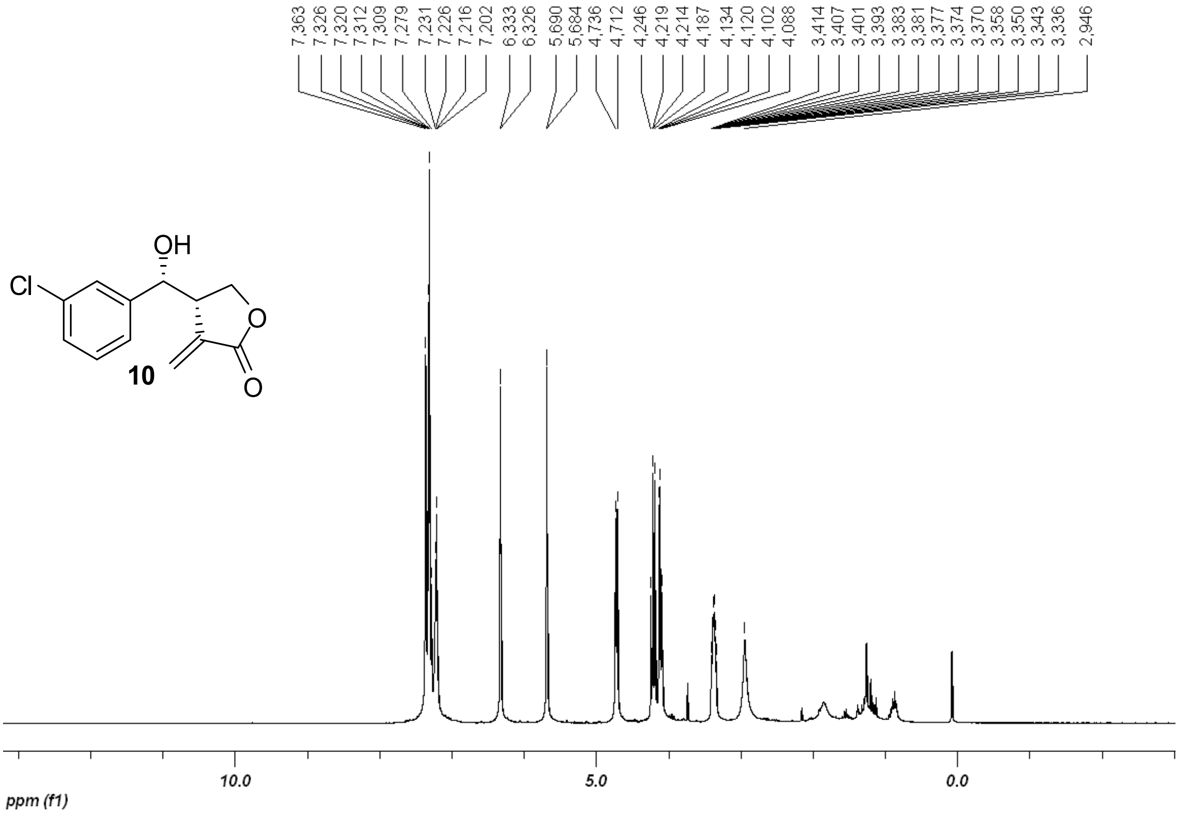

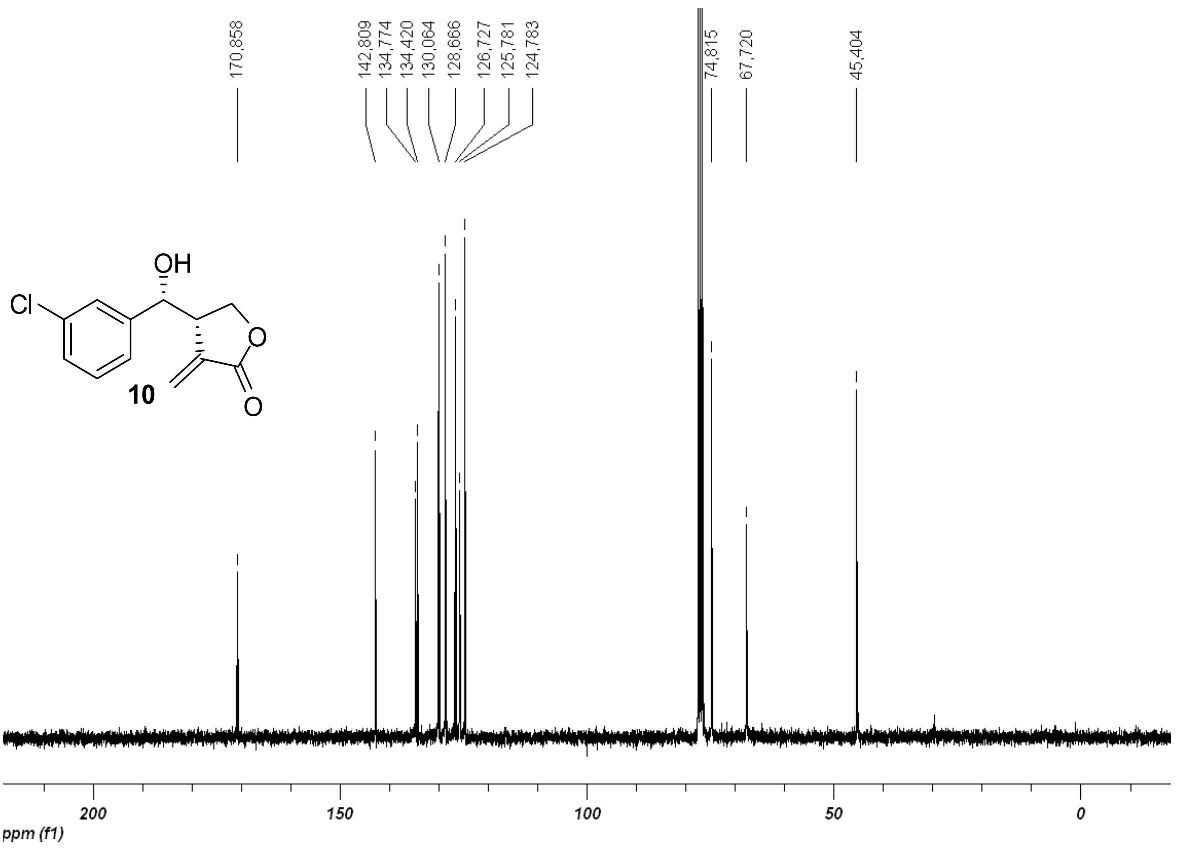

(*S*)-4-[(*R*)-(4-Fluorophenyl)(hydroxymethyl]-3-methylenedihydrofuran-2(3H)-one (11).

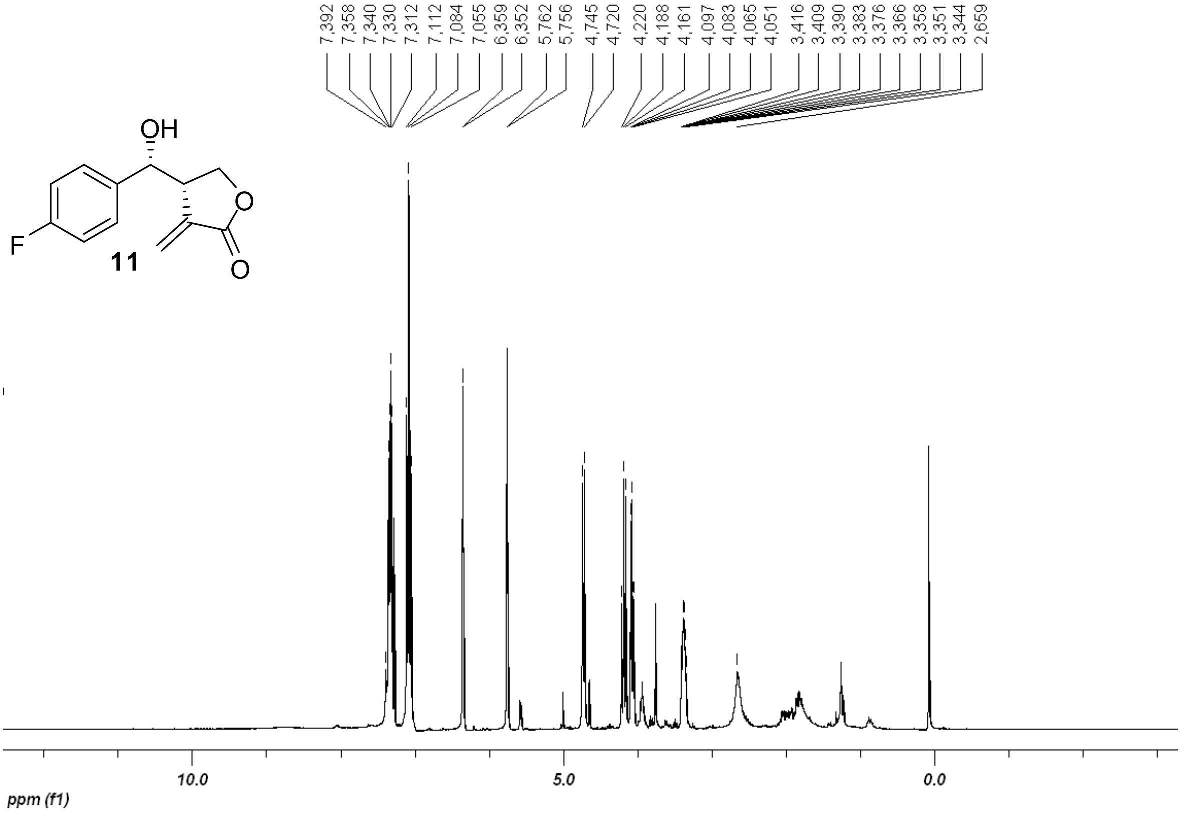

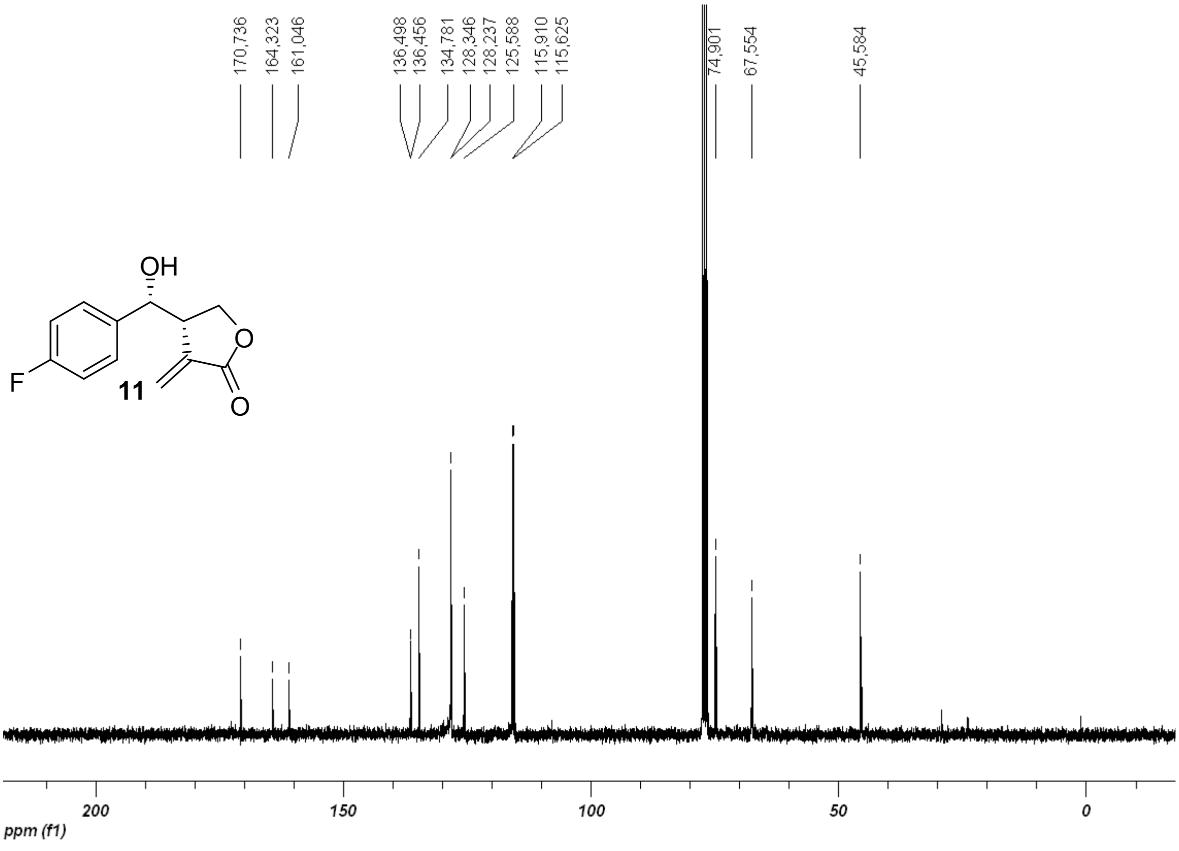

(*S*)-4-[(*R*)-(4-*tert*-Butylphenyl)(hydroxy)methyl]-3-methylenedihydrofuran-2(3H)-one (12).

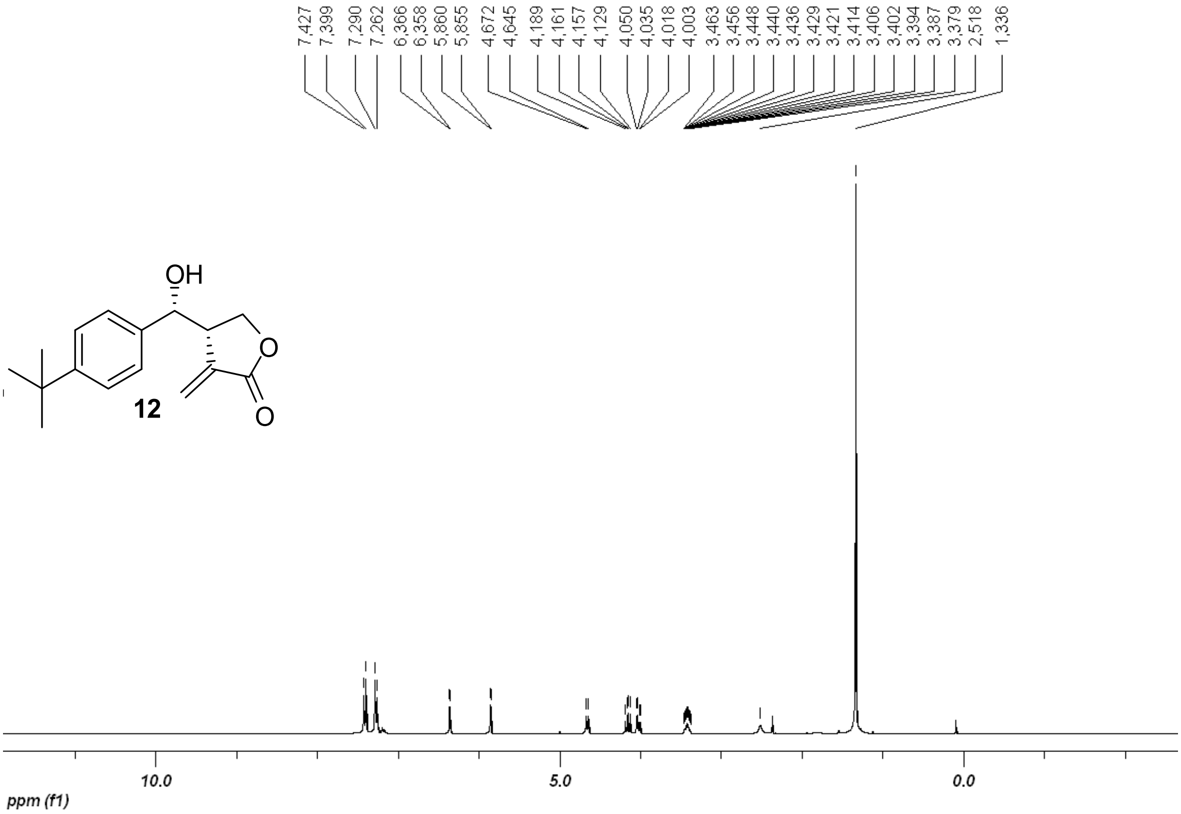

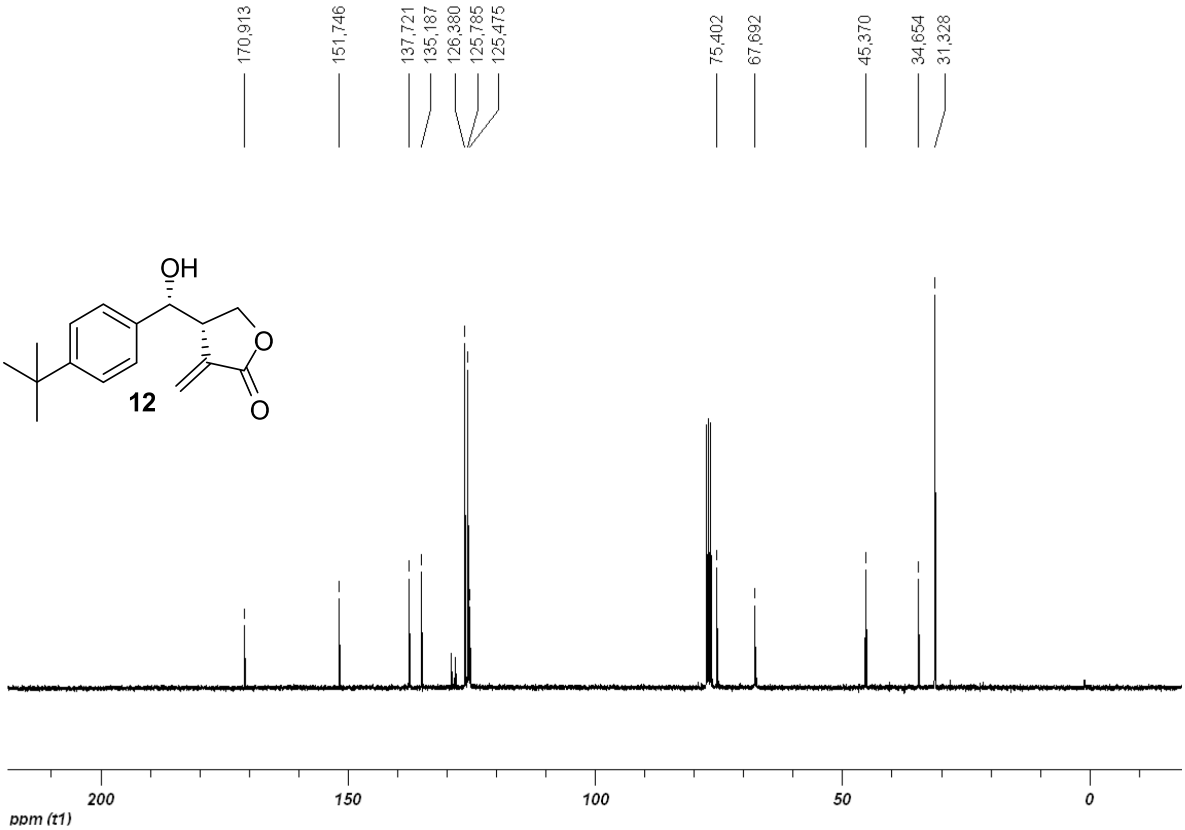

(*S*)-4-[(*R*)-Hydroxy(naphthalen-2-yl)methyl]-3-methylenedihydrofuran-2(3H)-one (13).

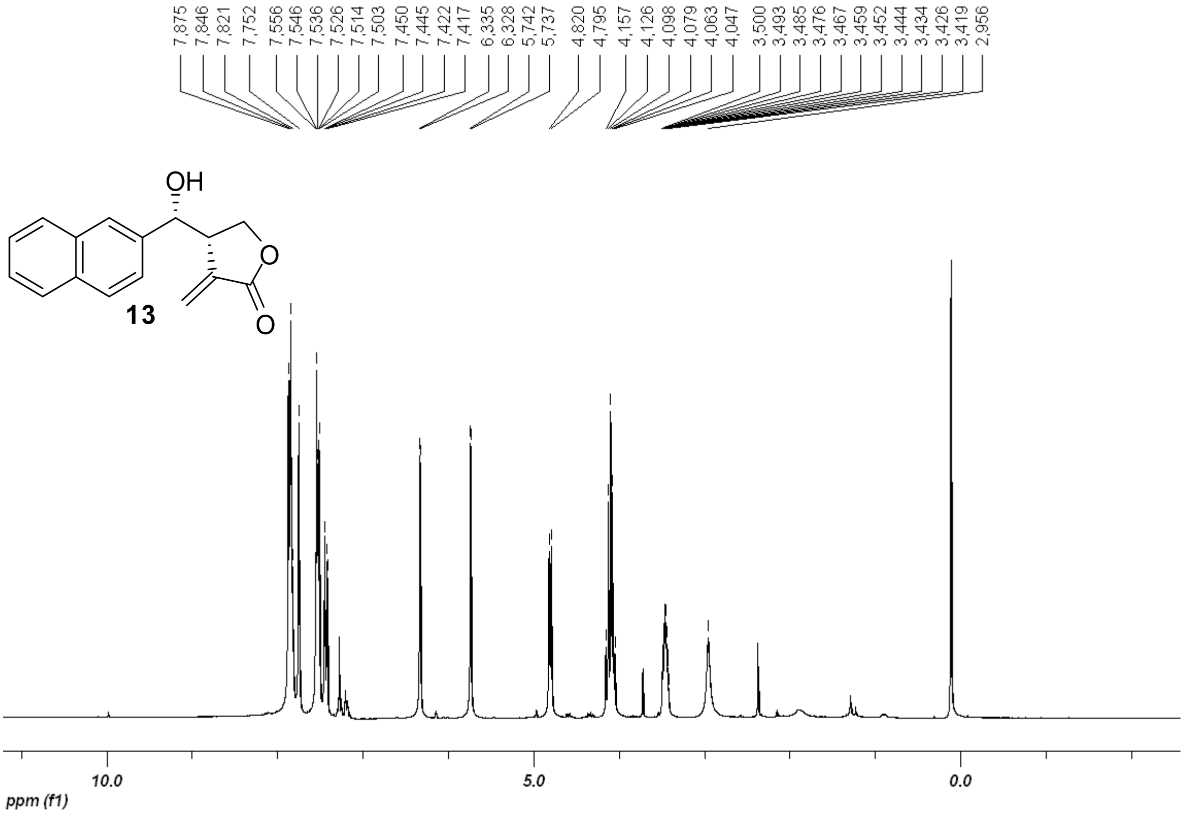

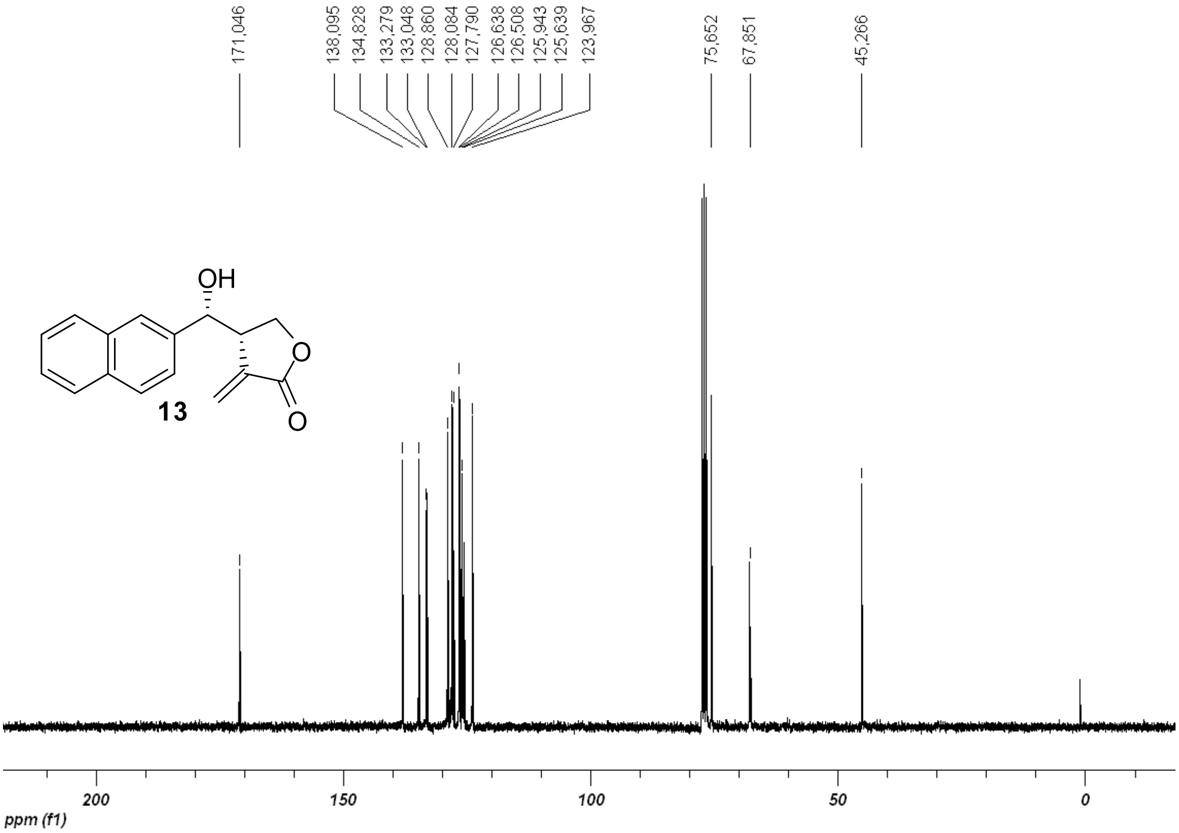

(*R*)-4-{(*S*)-[4-(Benzyloxy)-3-methoxyphenyl](hydroxy)methyl}-3-methylenedihydrofuran-2(3H)-one (6).

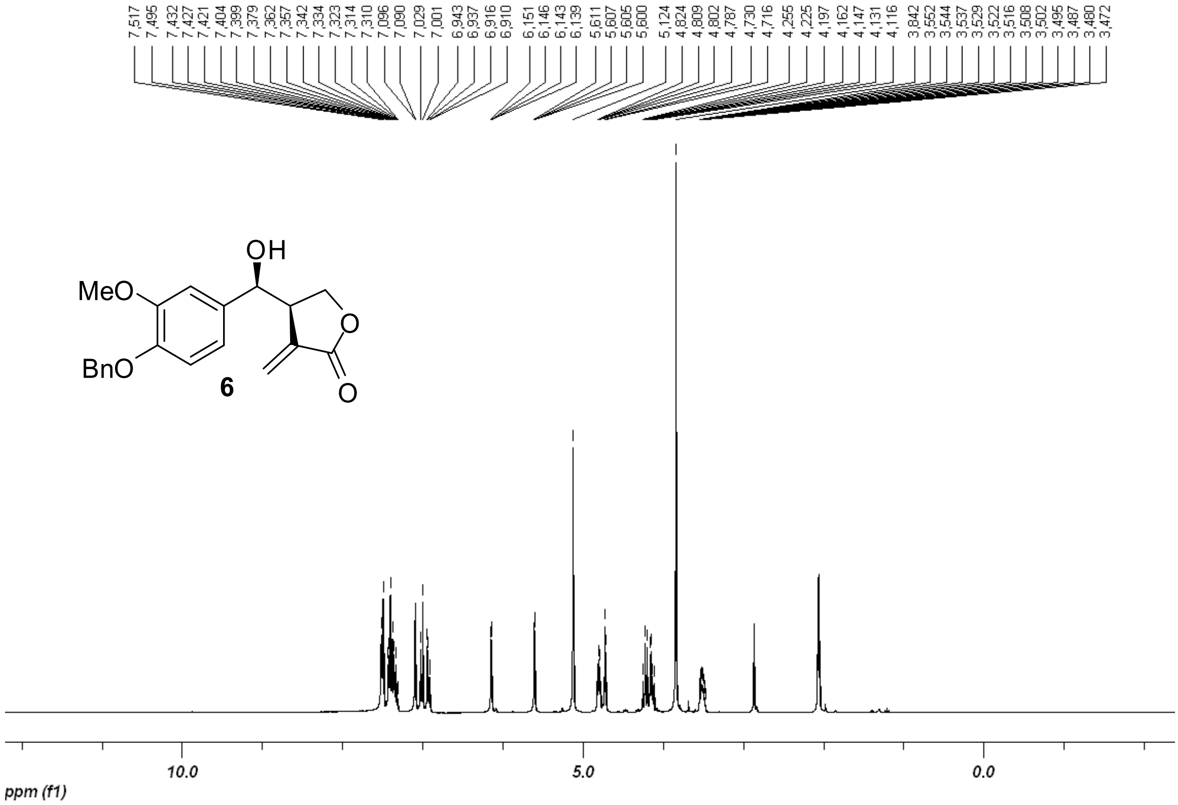

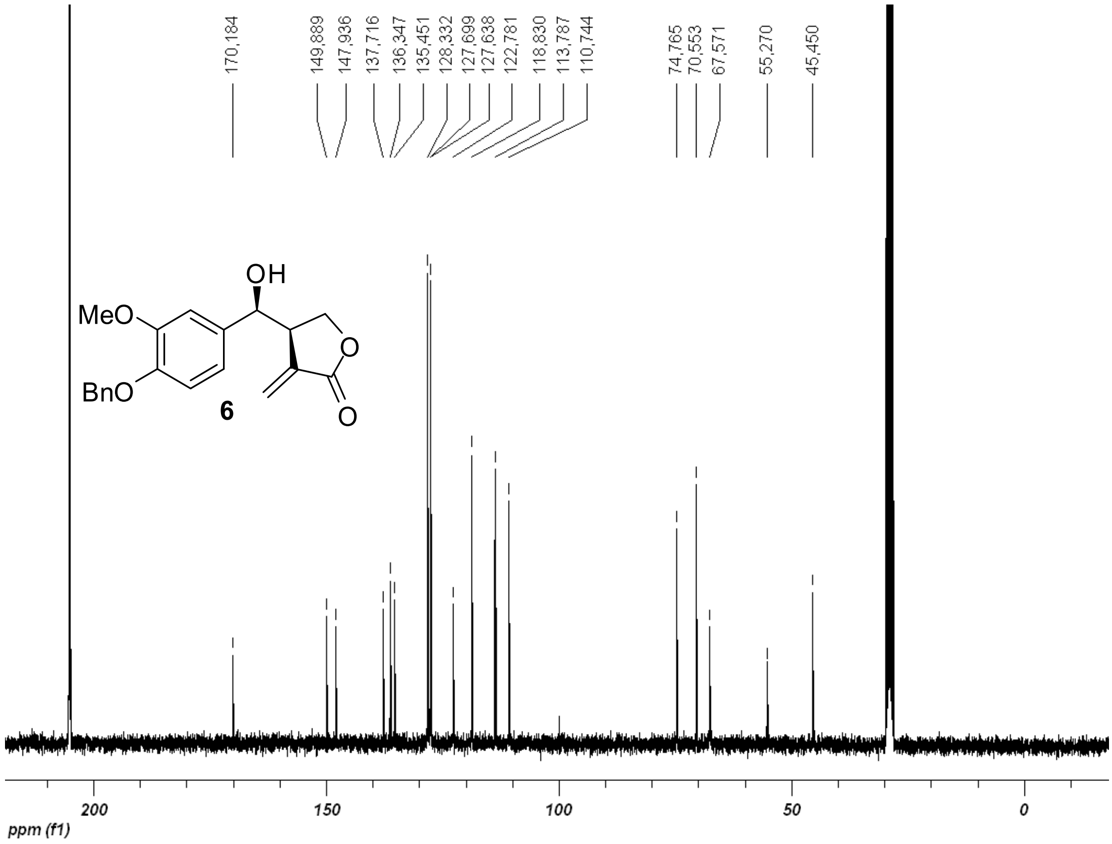

(3*R*,4*R*)-4-{(*S*)-[4-(Benzyloxy)-3-methoxyphenyl](hydroxy)methyl}-3-(4-hydroxy-3-methoxybenzyl)dihydrofuran-2(3H)-one (14).

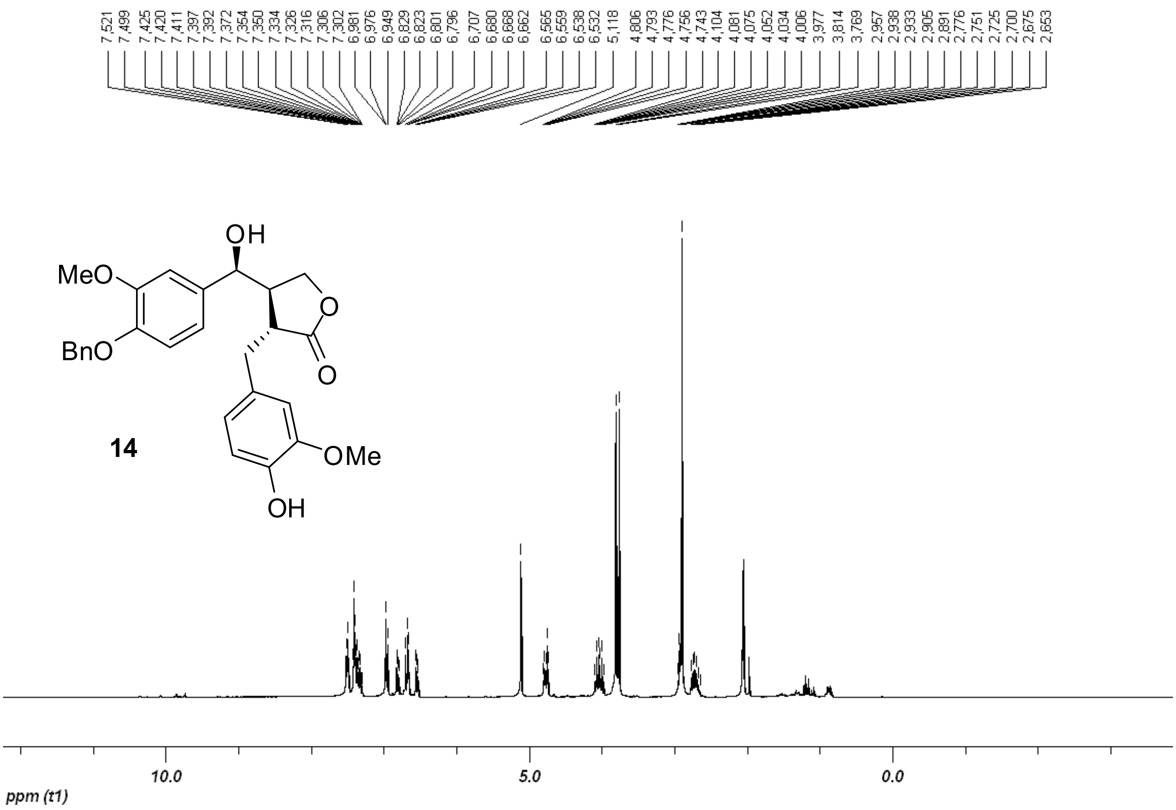

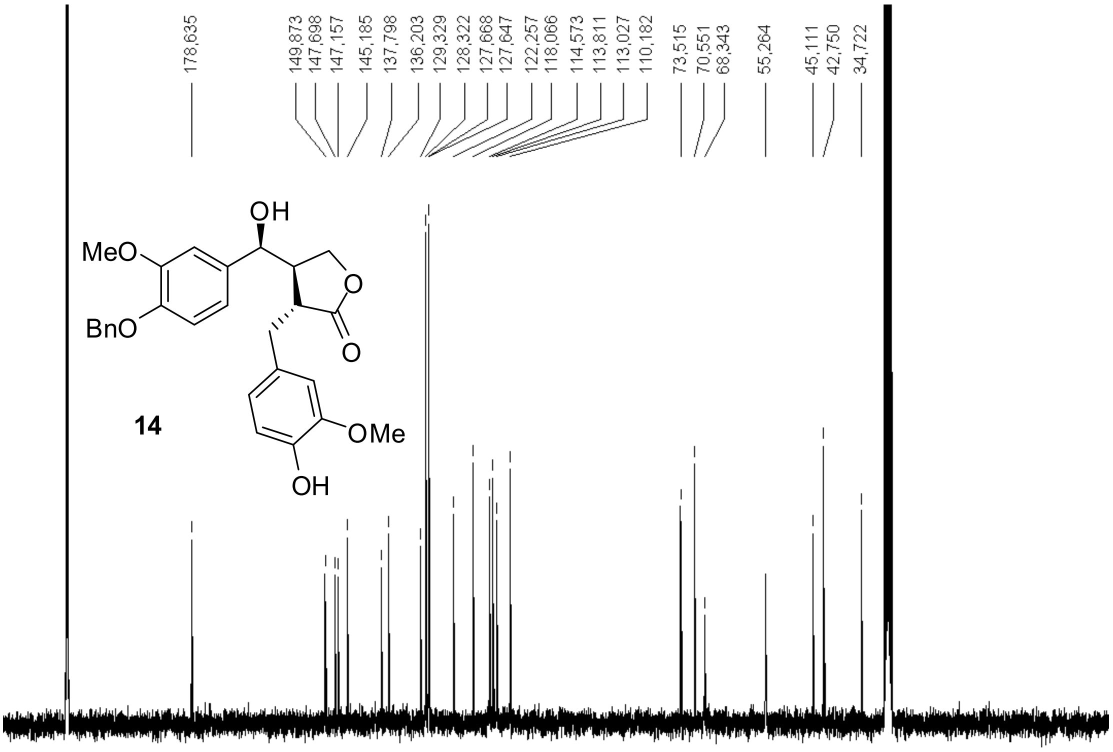

(7’*S*)-Hydroxymatairesinol [(7’*S*,8*R*,8’*R*)-4,4’,7’-trihydroxy-3,3’-dimethoxylignano-9,9’-lactone, (1)].

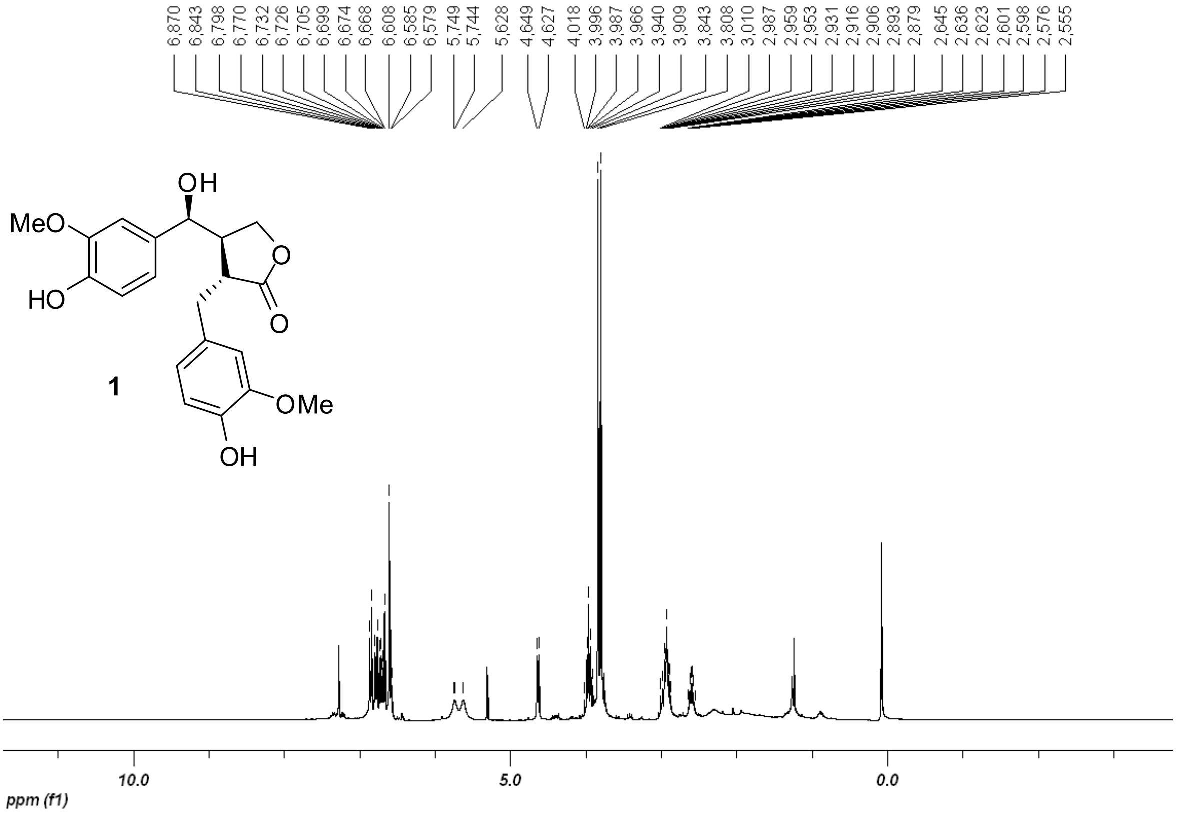

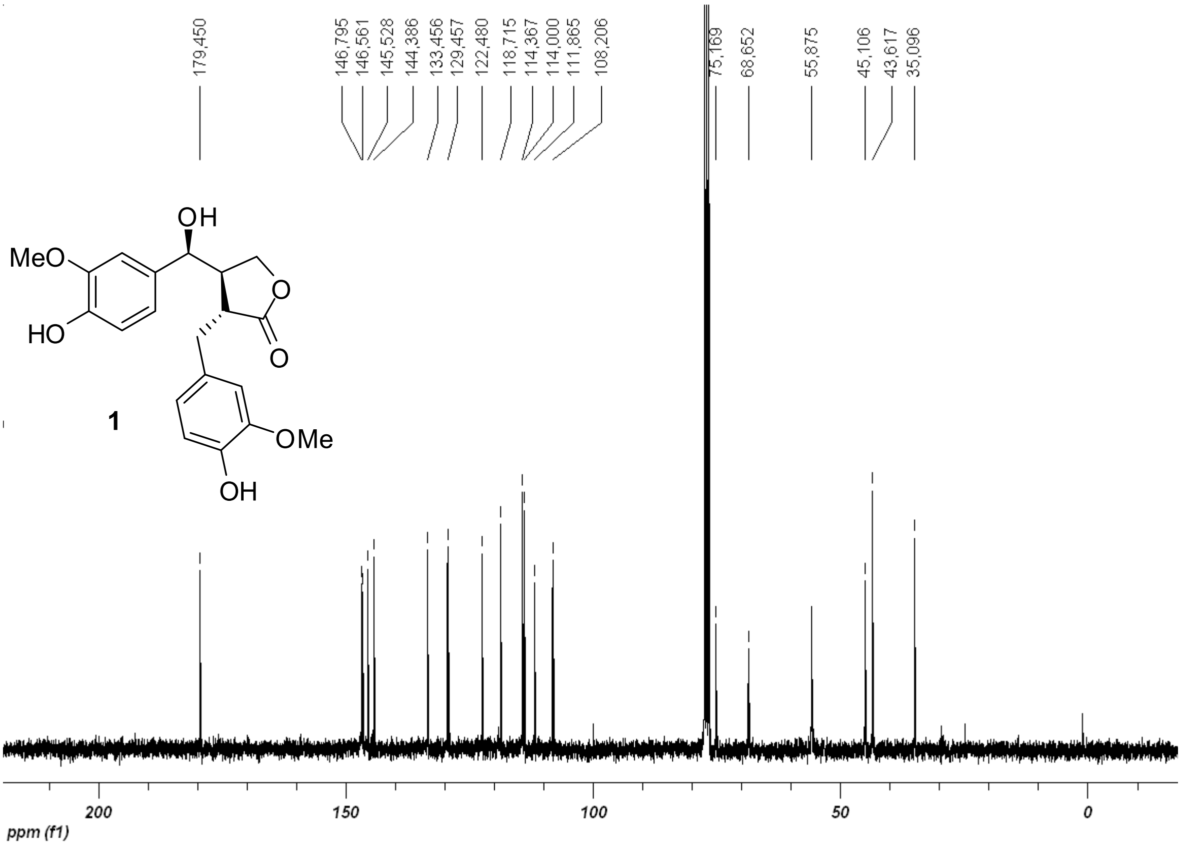

HPLC-UV Analysis on a chiral stationary phase

(*R*)-4-{(*S*)-[4-(Benzyloxy)-3-methoxyphenyl](hydroxy)methyl}-3-methylenedihydrofuran-2(3H)-one (6).

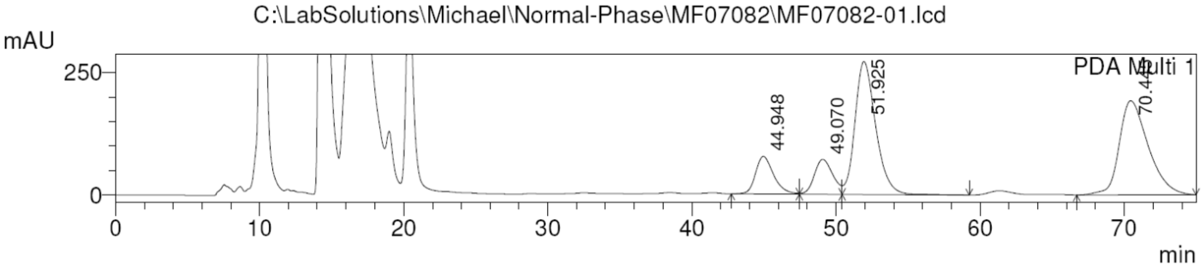

Peak#	Ret. Time	Area	Height	Area %	Height %
1	44.948	6718010	76810	10.000	12.566
2	49.070	6330566	71337	9.423	11.671
3	51.925	27203051	271199	40.492	44.368
4	70.445	26930085	191903	40.085	31.395
Total		67181712	611248	100.000	100.000

PeakTable

rac-**6**, *syn*:*anti* 20:80, acetylated sample

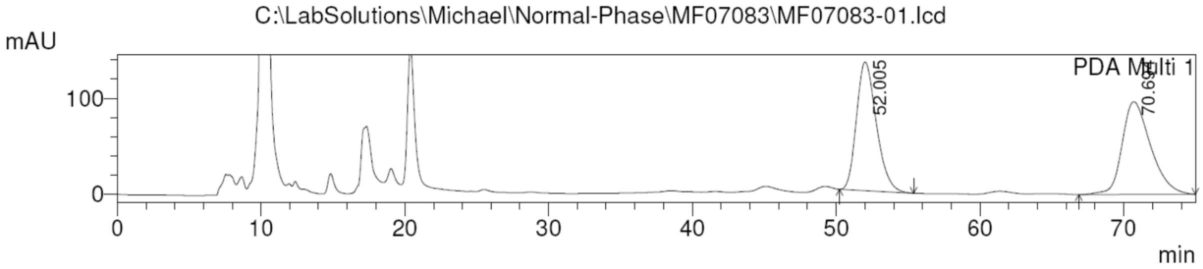

Peak#	Ret. Time	Area	Height	Area %	Height %
1	52.005	12914610	133999	49.171	58.150
2	70.694	13349927	96438	50.829	41.850
Total		26264537	230437	100.000	100.000

PeakTable

rac-**6**, *syn*:*anti* >95:5, acetylated sample

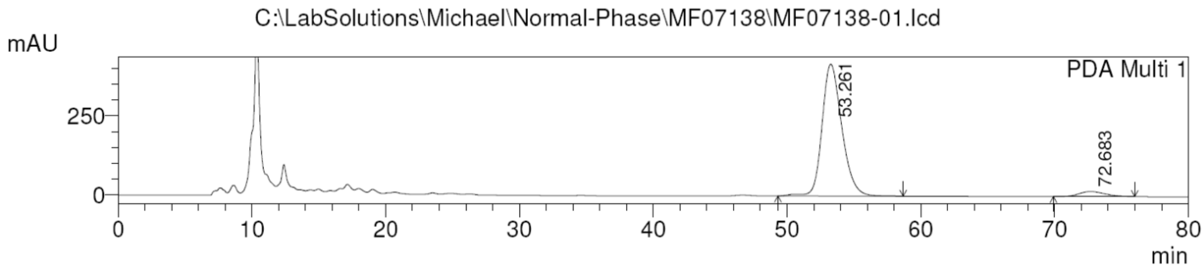

Peak#	Ret. Time	Area	Height	Area %	Height %
1	53.261	42969412	416866	95.270	96.332
2	72.683	2133542	15871	4.730	3.668
Total		45102954	432737	100.000	100.000

PeakTable

ent-**6** from asymmetric allylation, acetylated sample

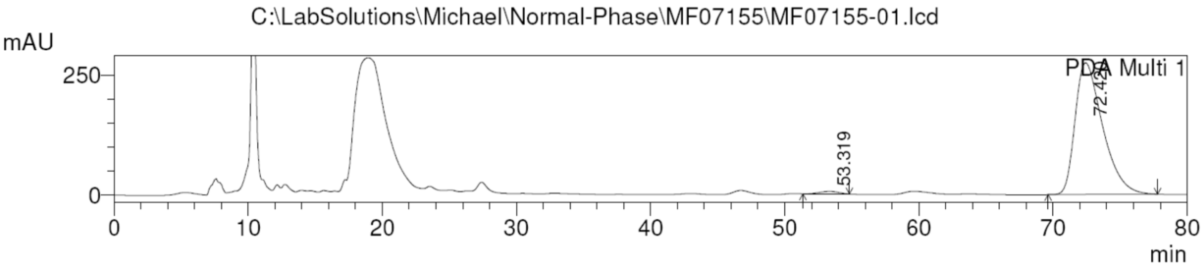

Peak#	Ret. Time	Area	Height	Area %	Height %
1	53.319	490433	5117	1.235	1.832
2	72.420	39223305	274252	98.765	98.168
Total		39713738	279369	100.000	100.000

PeakTable

6 from asymmetric allylation, acetylated sample

(*S*)-4-[(*R*)-Hydroxy(phenyl)methyl]-3-methylenedihydrofuran-2(3H)-one (7).

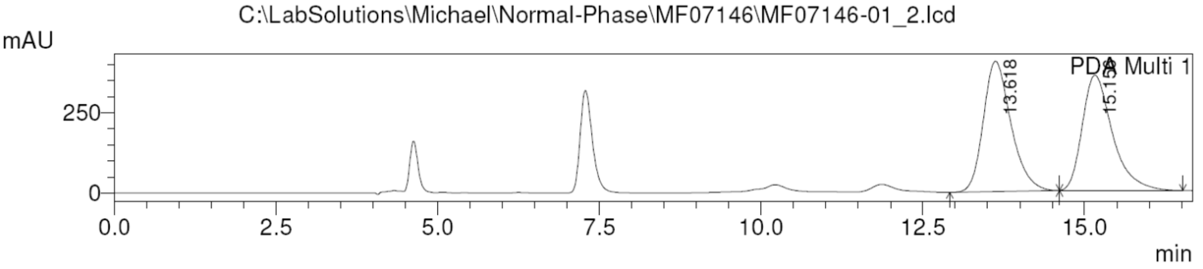

Peak#	Ret. Time	Area	Height	Area %	Height %
1	13.618	12089258	405945	50.881	53.074
2	15.156	11670689	358925	49.119	46.926
Total		23759947	764871	100.000	100.000

PeakTable

rac-**7**

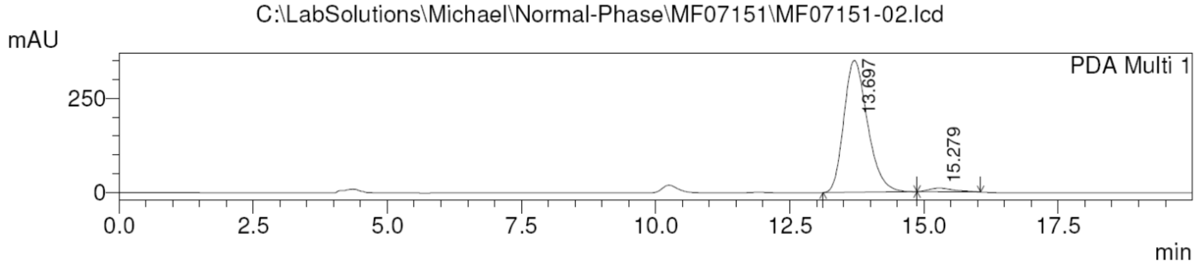

Peak#	Ret. Time	Area	Height	Area %	Height %
1	13.697	10760279	350133	97.171	97.346
2	15.279	313225	9546	2.829	2.654
Total		11073504	359679	100.000	100.000

PeakTable

7 from asymmetric allylation

(*S*)-4-[(*R*)-Hydroxy(4-methoxyphenyl)methyl]-3-methylenedihydrofuran-2(3H)-one (8).

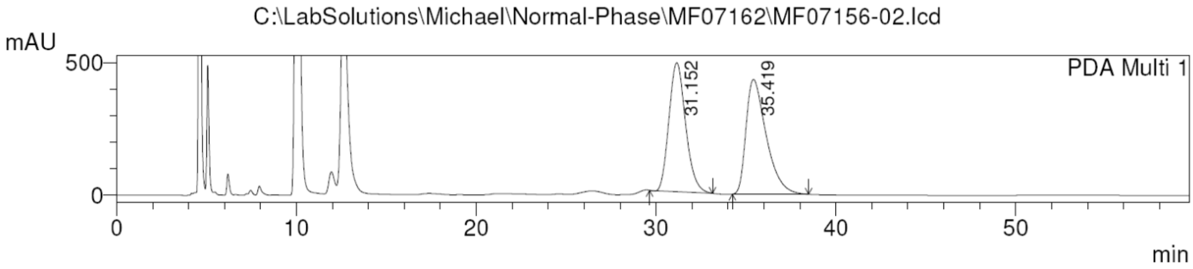

Peak#	Ret. Time	Area	Height	Area %	Height %
1	31.152	31240647	486135	47.971	52.904
2	35.419	33882774	432762	52.029	47.096
Total		65123421	918897	100.000	100.000

PeakTable

rac-**8**

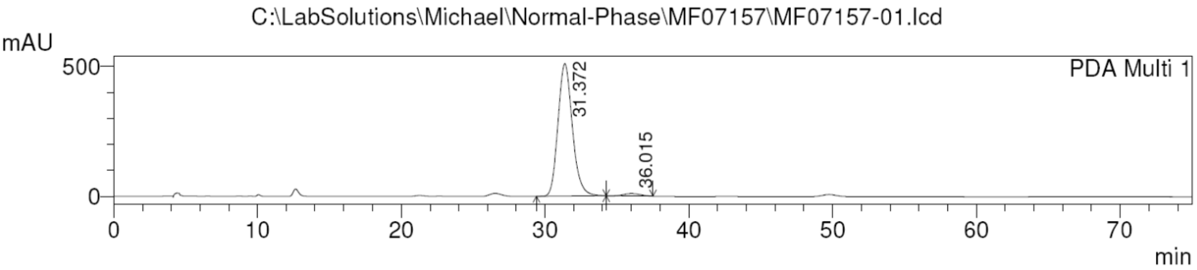

Peak#	Ret. Time	Area	Height	Area %	Height %
1	31.372	35551728	511752	97.990	98.205
2	36.015	729124	9354	2.010	1.795
Total		36280851	521106	100.000	100.000

PeakTable

8 from asymmetric allylation

(*S*)-4-[(*R*)-Furan-2-yl(hydroxy)methyl]-3-methylenedihydrofuran-2(3H)-one (9).

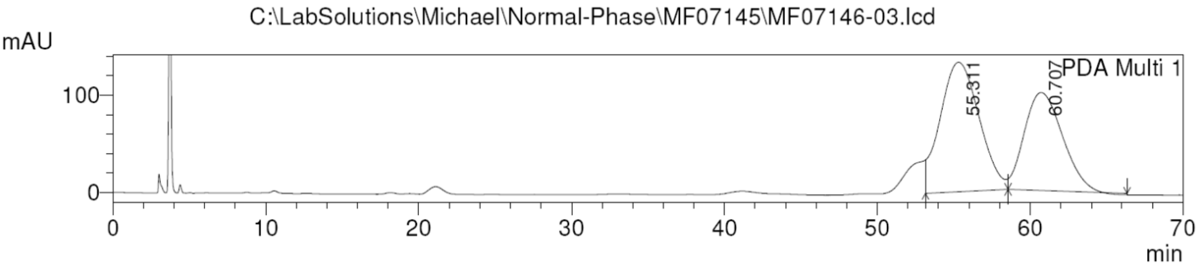

PDA Ch1 215nm 4nm

Peak#	Ret. Time	Area	Height	Area %	Height %
1	55.311	23735143	133470	57.551	56.952
2	60.707	17506521	100886	42.449	43.048
Total		41241665	234356	100.000	100.000

rac-**9**

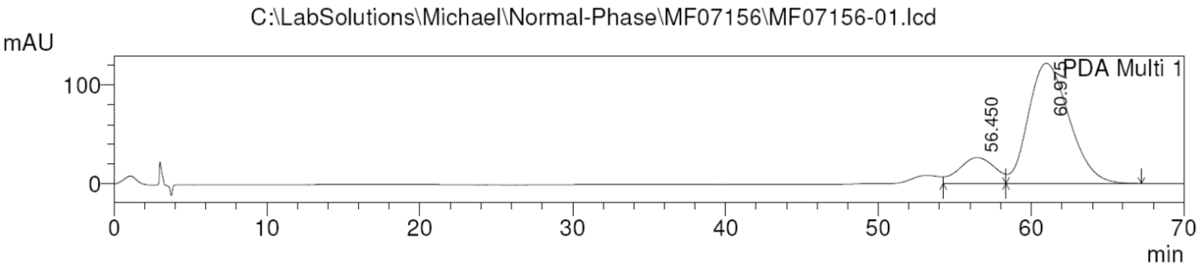

Peak#	Ret. Time	Area	Height	Area %	Height %
1	56.450	4252214	26307	15.810	17.755
2	60.975	22642873	121857	84.190	82.245
Total		26895087	148164	100.000	100.000

PeakTable

9 from asymmetric allylation

(*S*)-4-[(*R*)-(3-Chlorophenyl)(hydroxy)methyl]-3-methylenedihydrofuran-2(3H)-one (10).

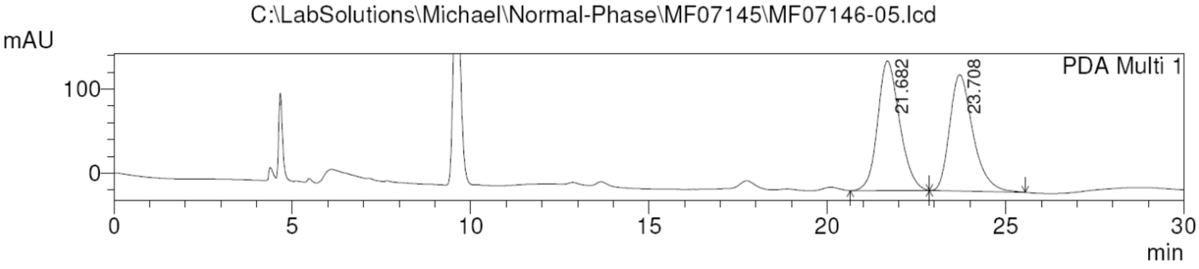

Peak#	Ret. Time	Area	Height	Area %	Height %
1	21.682	6619413	154386	51.228	52.706
2	23.708	6302045	138534	48.772	47.294
Total		12921458	292920	100.000	100.000

PeakTable

rac-**10**

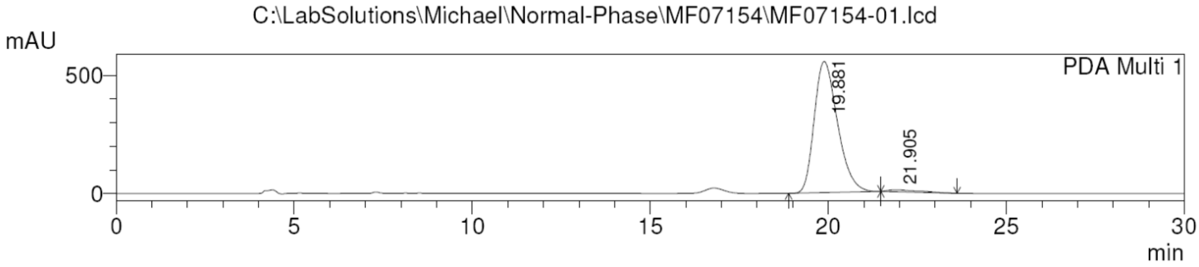

Peak#	Ret. Time	Area	Height	Area %	Height %
1	19.881	25194880	553377	98.076	98.543
2	21.905	494374	8179	1.924	1.457
Total		25689255	561556	100.000	100.000

PeakTable

10 from asymmetric allylation

(*S*)-4-[(*R*)-(4-Fluorophenyl)(hydroxy)methyl]-3-methylenedihydrofuran-2(3H)-one (11).

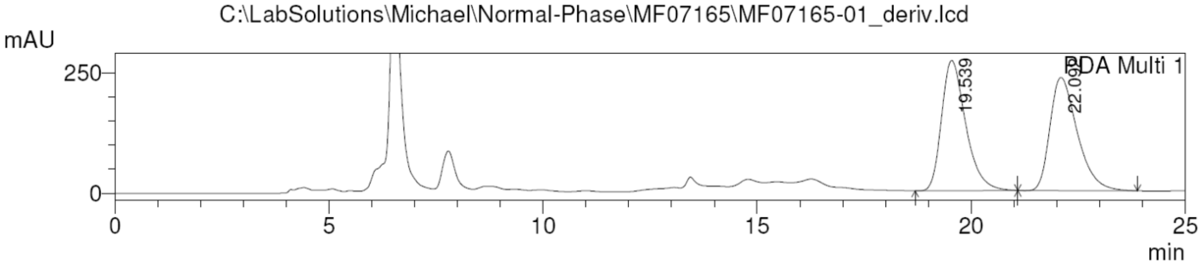

Peak#	Ret. Time	Area	Height	Area %	Height %
1	19.539	10634533	270436	50.454	53.496
2	22.092	10443254	235087	49.546	46.504
Total		21077787	505523	100.000	100.000

PeakTable

rac-**11,** acetylated sample

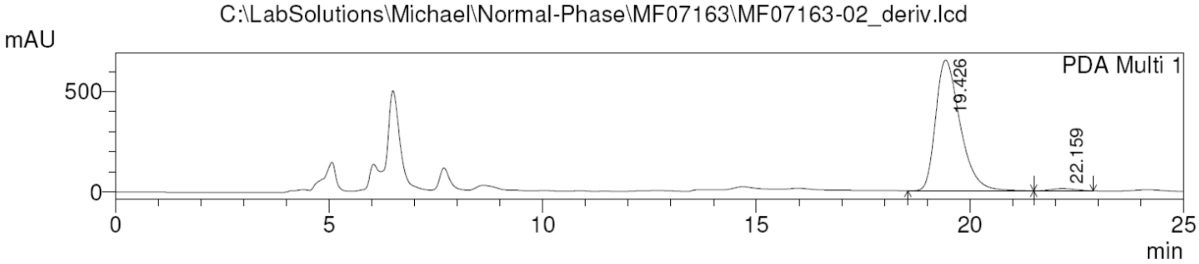

Peak#	Ret. Time	Area	Height	Area %	Height %
1	19.426	25824735	650046	98.501	98.419
2	22.159	393004	10442	1.499	1.581
Total		26217739	660488	100.000	100.000

PeakTable

11 from asymmetric allylation, acetylated sample

(*S*)-4-[(*R*)-(4-*tert*-Butylphenyl)(hydroxy)methyl]-3-methylenedihydrofuran-2(3H)-one (12).

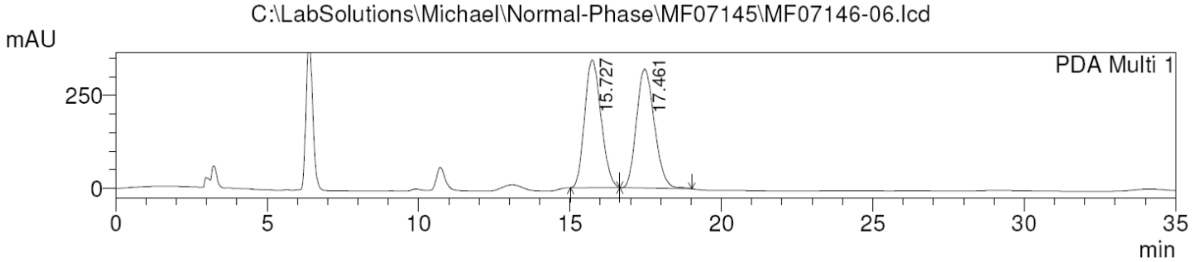

Peak#	Ret. Time	Area	Height	Area %	Height %
1	15.727	12969591	344019	49.576	51.797
2	17.461	13191482	320146	50.424	48.203
Total		26161073	664165	100.000	100.000

PeakTable

rac-**12**

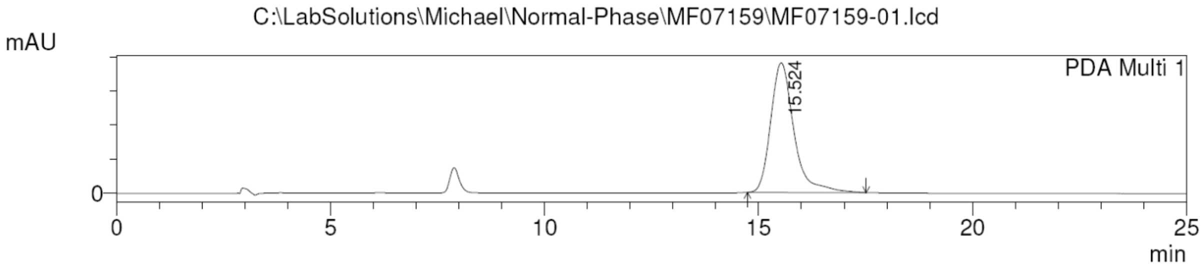

Peak#	Ret. Time	Area	Height	Area %	Height %
1	15.524	7178732	189997	100.000	100.000
Total		7178732	189997	100.000	100.000

PeakTable

12 from asymmetric allylation

(*S*)-4-[(*R*)-Hydroxy(naphthalen-2-yl)methyl]-3-methylenedihydrofuran-2(3H)-one (13).

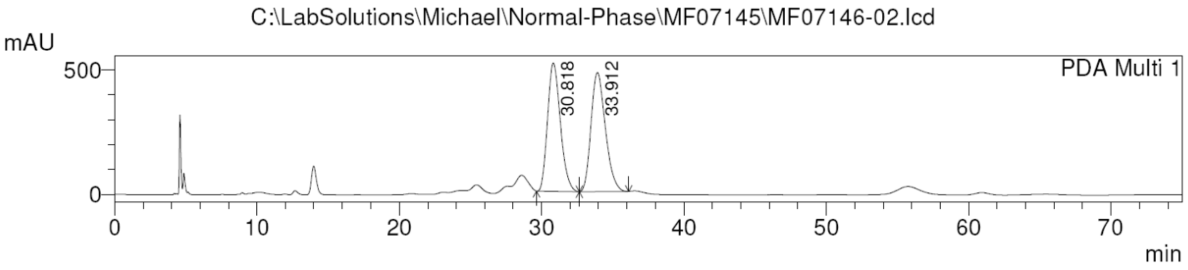

Peak#	Ret. Time	Area	Height	Area %	Height %
1	30.818	32356436	515700	49.068	51.859
2	33.912	33585383	478720	50.932	48.141
Total		65941819	994420	100.000	100.000

PeakTable

rac-**13**

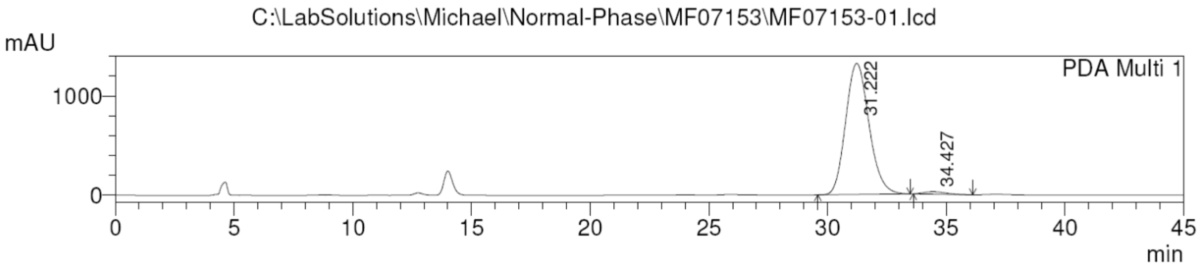

Peak#	Ret. Time	Area	Height	Area %	Height %
1	31.222	90653269	1320870	98.343	98.222
2	34.427	1527455	23909	1.657	1.778
Total		92180724	1344778	100.000	100.000

PeakTable

13 from asymmetric allylation

(3*R*,4*R*)-4-{(*S*)-[4-(Benzyloxy)-3-methoxyphenyl](hydroxy)methyl}-3-(4-hydroxy-3-methoxybenzyl)dihydrofuran-2(3H)-one (14).

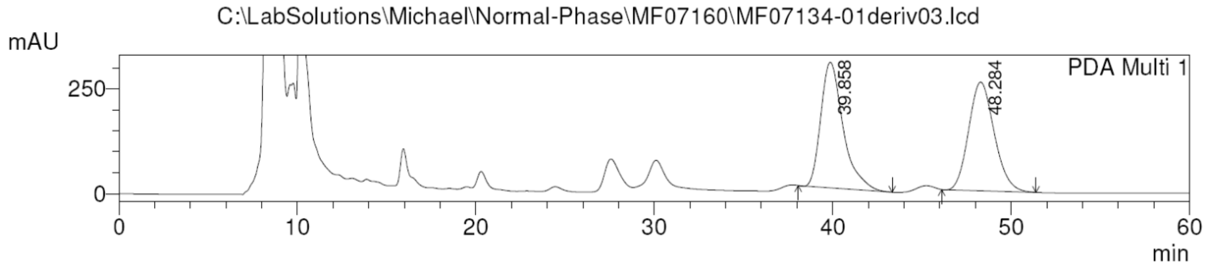

Peak#	Ret. Time	Area	Height	Area %	Height %
1	39.858	26134339	299628	50.240	53.610
2	48.284	25884987	259270	49.760	46.390
Total		52019326	558897	100.000	100.000

PeakTable

rac-**14**, acetylated sample

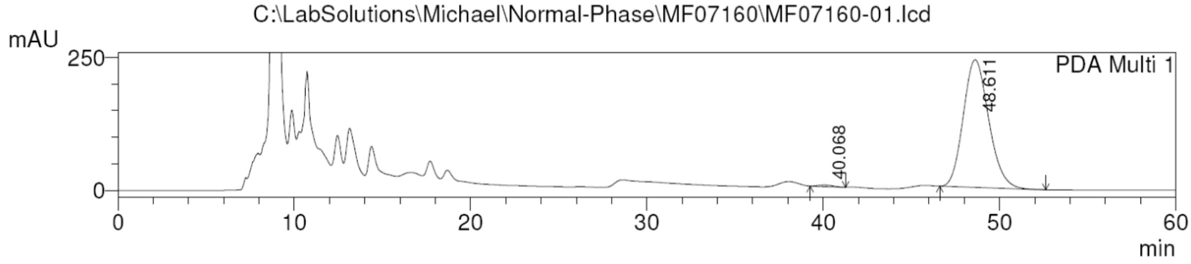

Peak#	Ret. Time	Area	Height	Area %	Height %
1	40.068	153752	2878	0.631	1.180
2	48.611	24208129	240982	99.369	98.820
Total		24361881	243860	100.000	100.000

PeakTable

(3*R*,4*R*)-**14**, acetylated sample

(7'*S*)-Hydroxymatairesinol [(7'*S*,8*R*,8'*R*)-4,4',7'-trihydroxy-3,3'-dimethoxylignano-9,9'-lactone (1)].

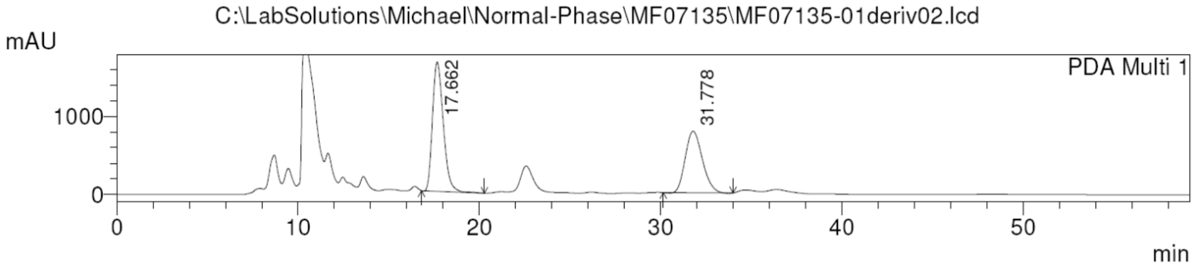

Peak#	Ret. Time	Area	Height	Area %	Height %
1	17.662	69148679	1663176	57.153	67.756
2	31.778	51839707	791487	42.847	32.244
Total		120988387	2454663	100.000	100.000

PeakTable

rac-**1**, acetylated sample (remark: to confirm 14 min separation, the racemate was checked at 4 different wavelengths and the UV-spectra of both peaks were identical)

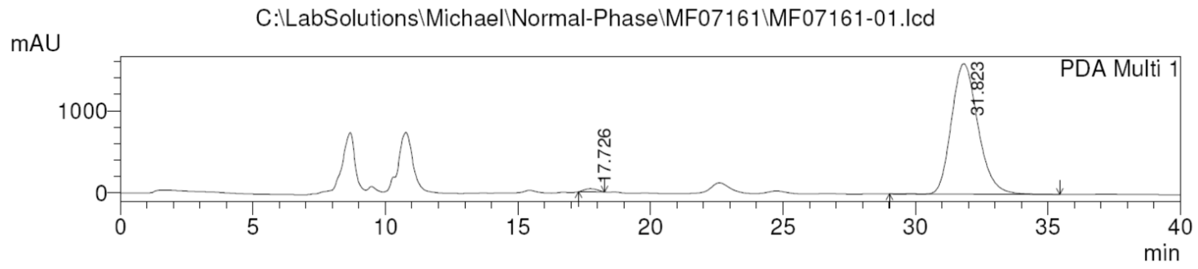

Peak#	Ret. Time	Area	Height	Area %	Height %
1	17.726	1316862	37912	1.174	2.331
2	31.823	110876450	1588621	98.826	97.669
Total		112193312	1626534	100.000	100.000

PeakTable

(7’*S*)-Hydroxymatairesinol (**1**), acetylated sample

CD-spectra

(*S*)-4-{(*R*)-[4-(Benzyloxy)-3-methoxyphenyl](hydroxy)methyl}-3-methylenedihydrofuran-2(3H)-one (ent-6).

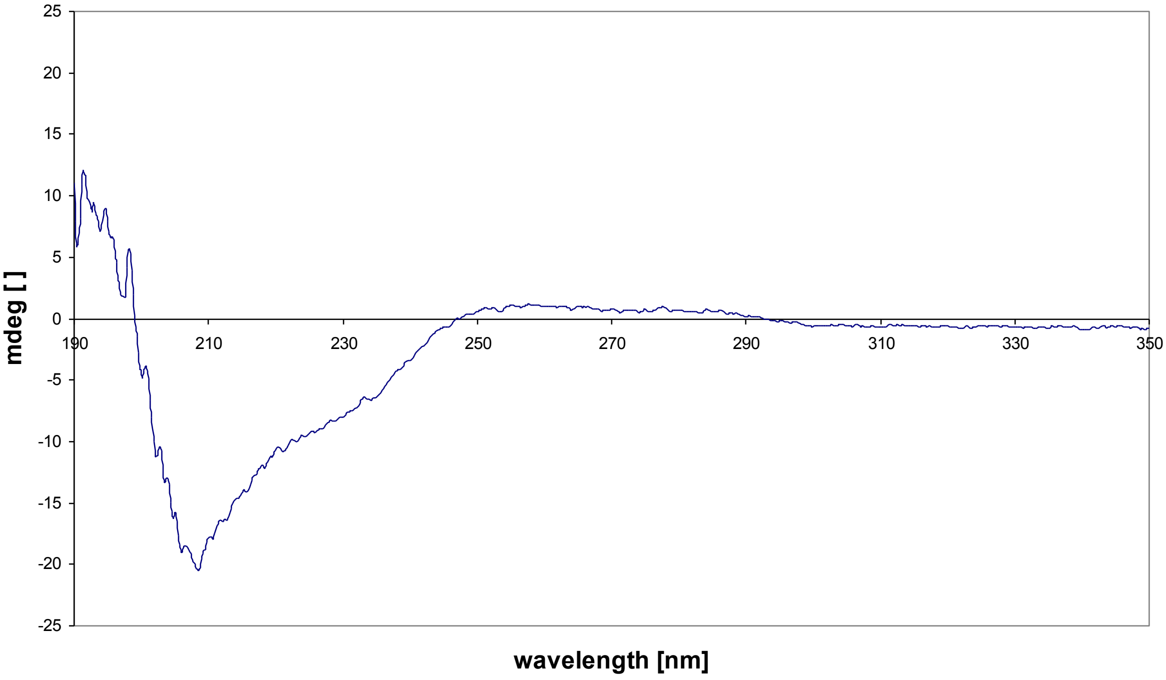

c = 1.0 mg/mL

(*R*)-4-{(*S*)-[4-(Benzyloxy)-3-methoxyphenyl](hydroxy)methyl}-3-methylenedihydrofuran-2(3H)-one (6).

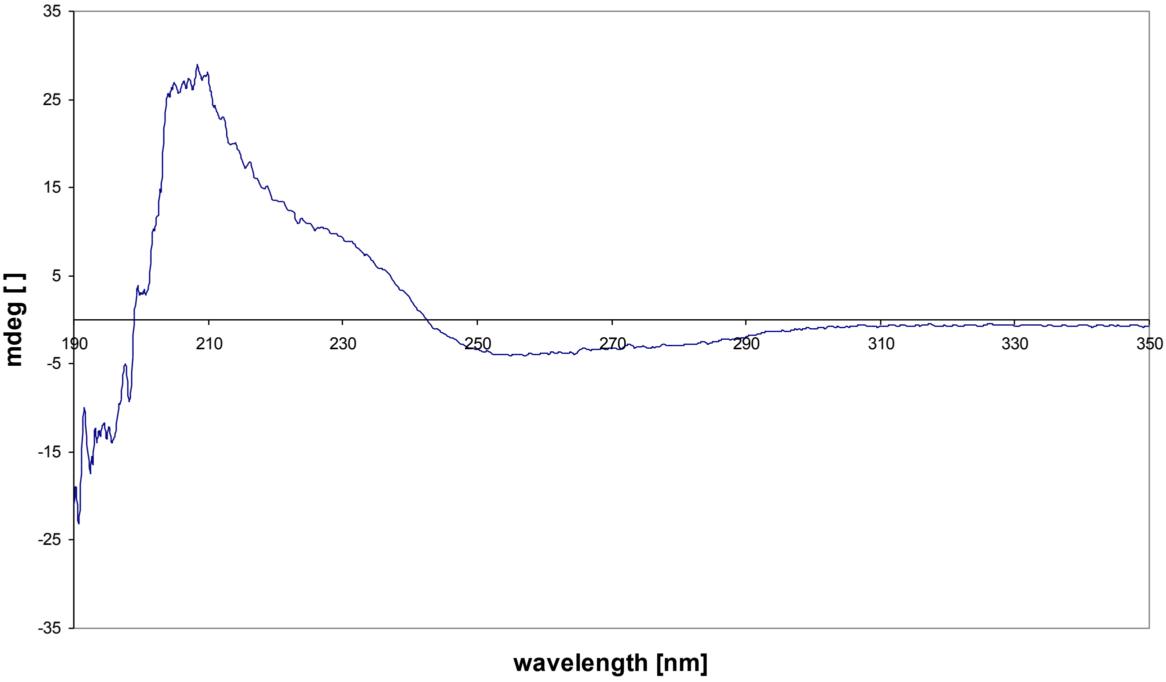

c = 1.0 mg/mL

(*S*)-4-[(*R*)-Hydroxy(phenyl)methyl]-3-methylenedihydrofuran-2(3H)-one (7).

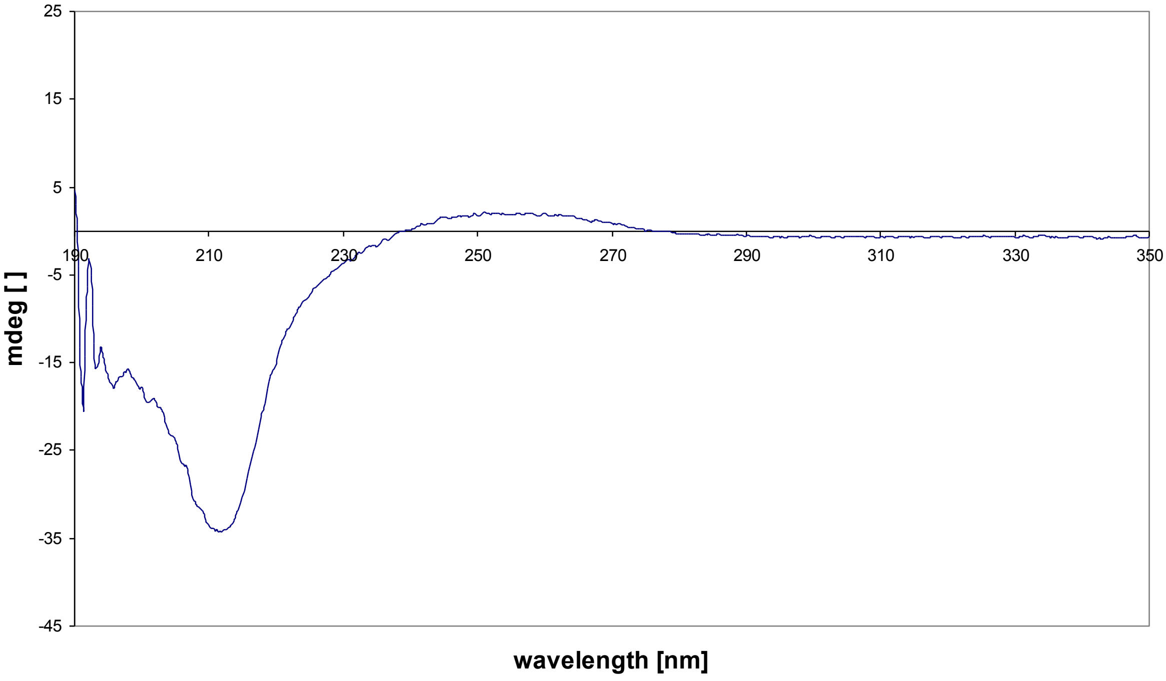

c = 1.05 mg/mL

(*S*)-4-[(*R*)-Hydroxy(4-methoxyphenyl)methyl]-3-methylenedihydrofuran-2(3H)-one (8).

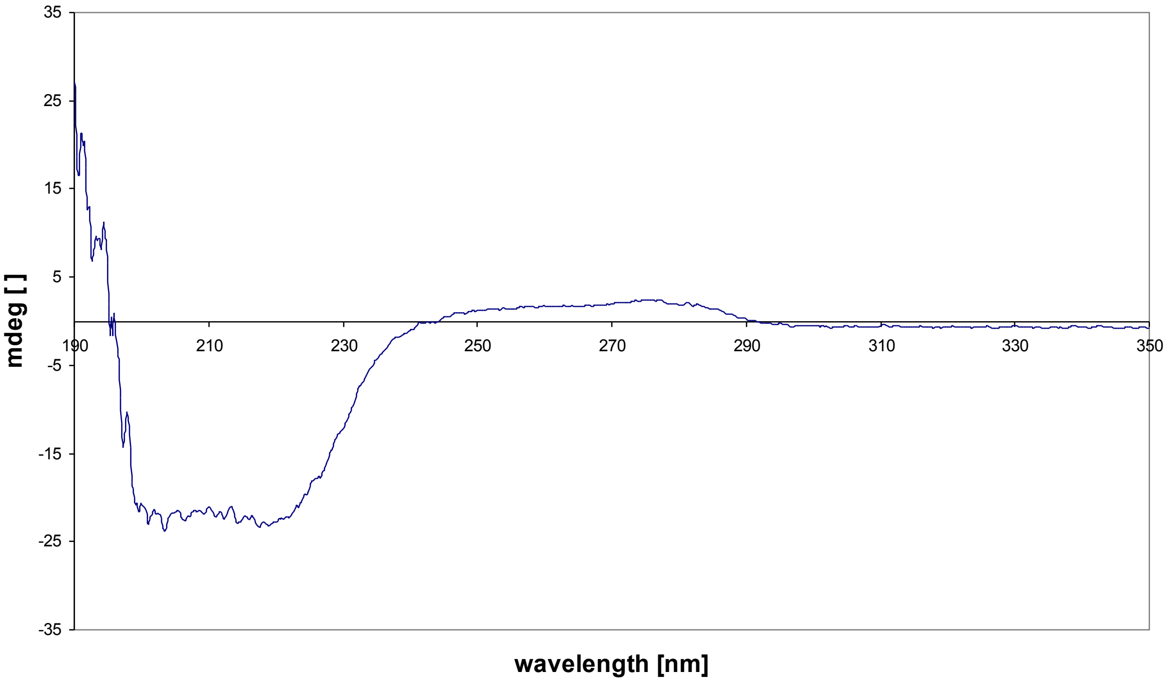

c = 1.0 mg/mL

(*S*)-4-[(*R*)-Furan-2-yl(hydroxy)methyl]-3-methylenedihydrofuran-2(3H)-one (9).

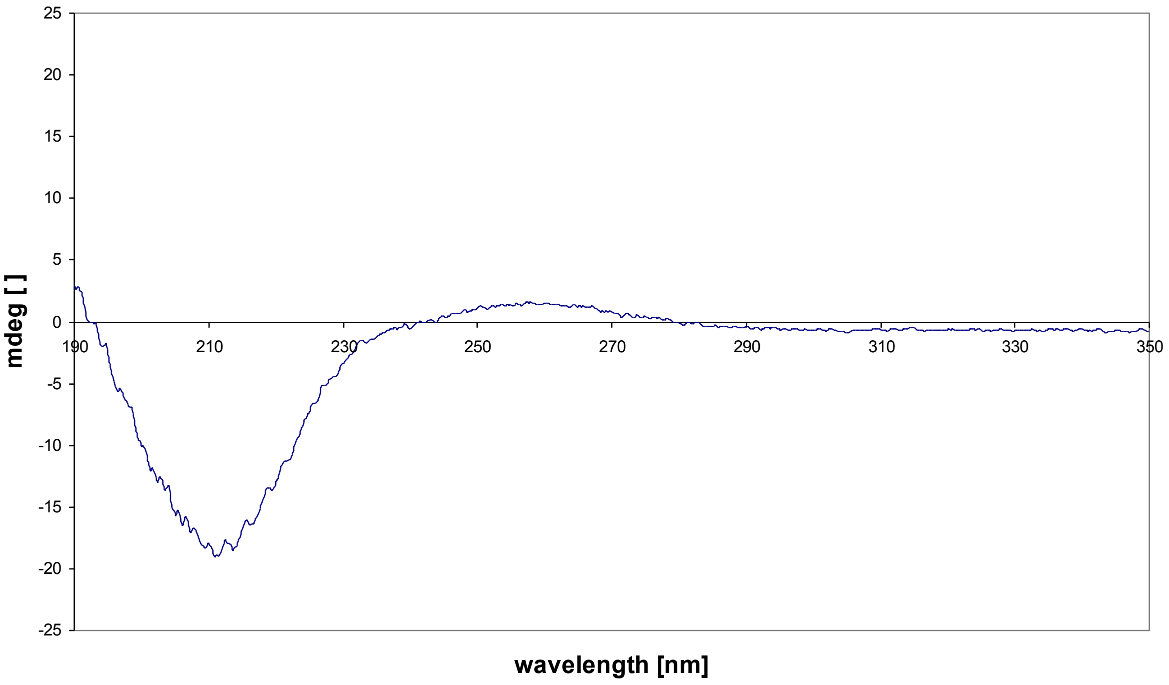

c = 1.05 mg/mL

(*S*)-4-[(*R*)-(3-Chlorophenyl)(hydroxy)methyl]-3-methylenedihydrofuran-2(3H)-one (10).

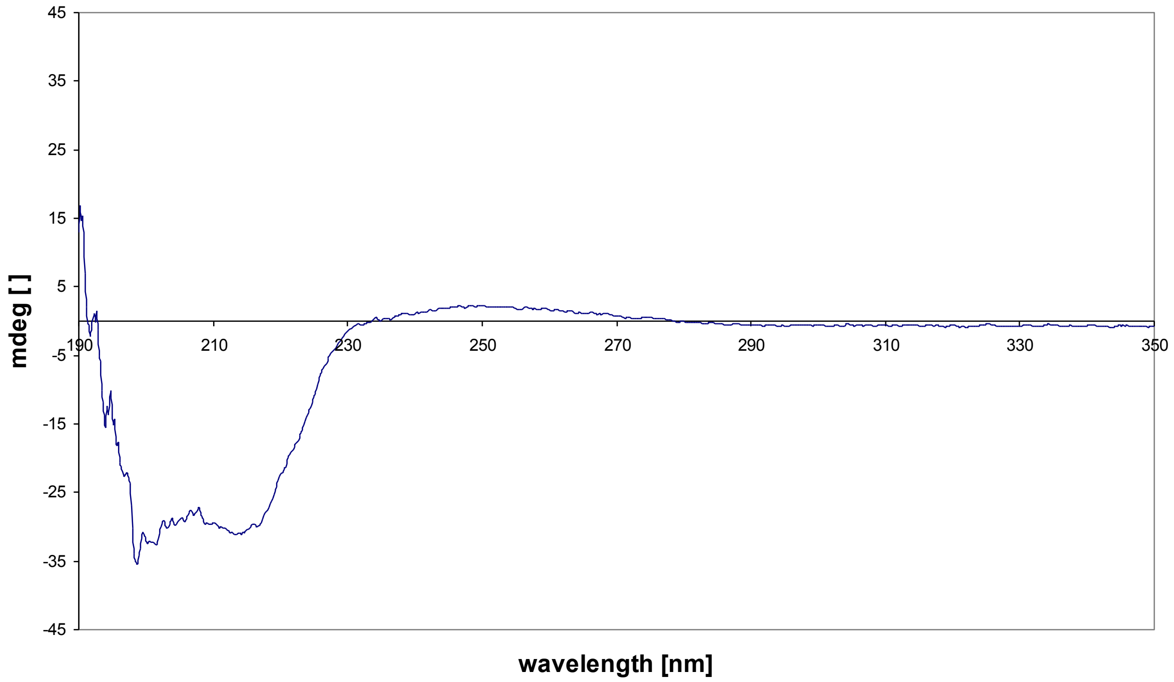

c = 1.15 mg/mL

(*S*)-4-[(*R*)-(4-Fluorophenyl)(hydroxy)methyl]-3-methylenedihydrofuran-2(3H)-one (11).

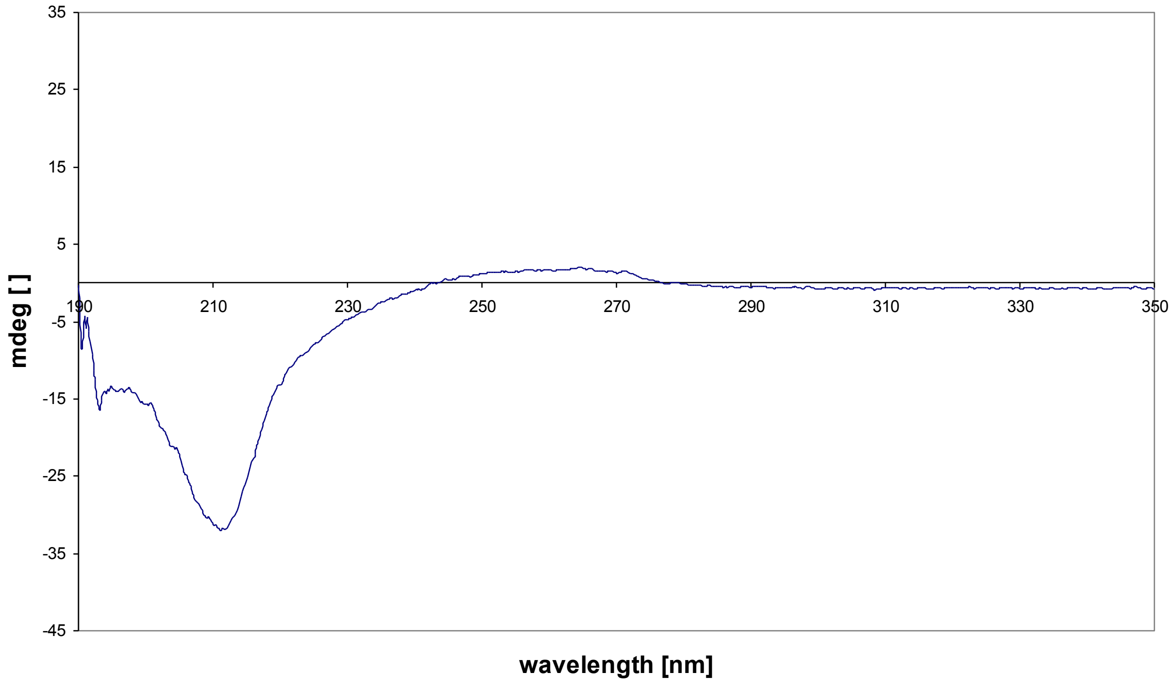

c = 1.1 mg/mL

(*S*)-4-[(*R*)-(4-*tert*-Butylphenyl)(hydroxy)methyl]-3-methylenedihydrofuran-2(3H)-one (12).

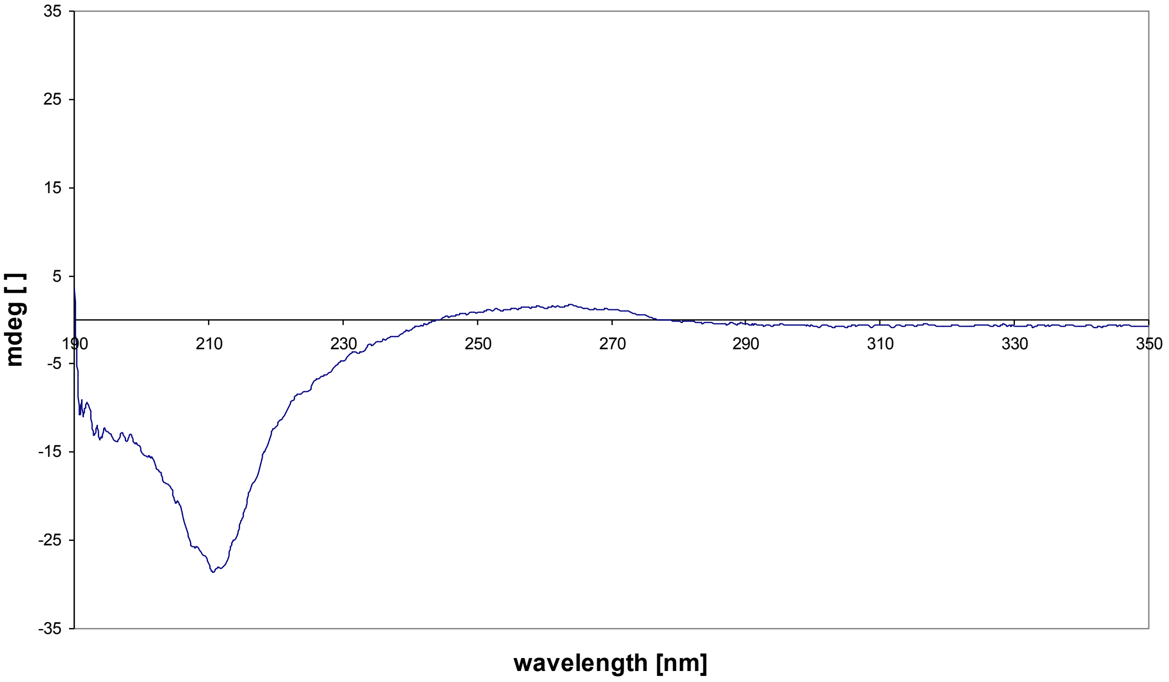

c = 0.8 mg/mL

(*S*)-4-[(*R*)-Hydroxy(naphthalen-2-yl)methyl]-3-methylenedihydrofuran-2(3H)-one (13).

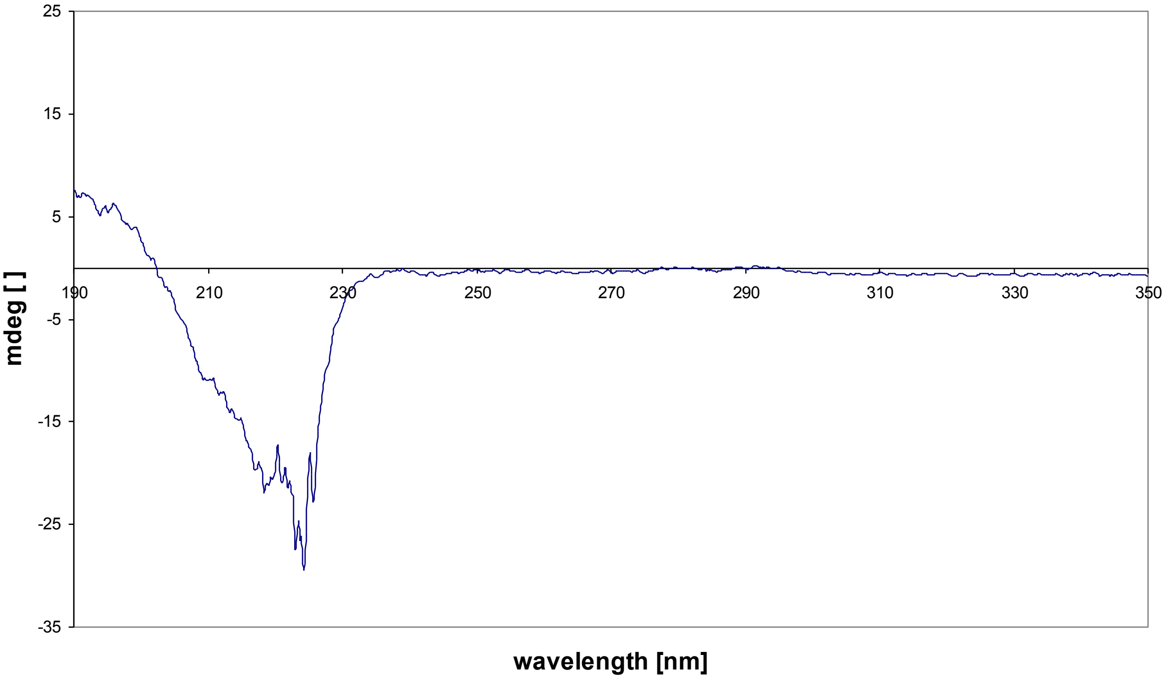

c = 0.8 mg/mL

Additional Computational Details

All computations were carried out with the Gaussian suite of programs (G09 Rev. C.01)[5] using a triple-ζ basis set (6-311G**) and the Minnesota M06 functional.[6] Optimized structures were determined to be true minima by inspection of their vibrational frequencies. Solvent effects were mimicked using the Polarizable Continuum Model (IEF-PCM).[7]

Table S05. Comparison of calculated structures and their energies

Compound	Gas phase/ solvent	stereo- chemistry	abs. Energy [hartree]	Rel. Energy [kcal/mol]	
	Gas phase comparison of isomers				
**TRIP$^-$**	g.p.	S_{ax}	-2579.410741	-	Fully optim.
**LacZnBrH**	g.p.	$R_{alc}S_{lac}$	-5042.743065	-	
**TRIP$^-$+ LacZnBrH**	g.p.	S_{ax} + $R_{alc}S_{lac}$	-7622.153806	239	
**Isomer A**	g.p.	$S_{ax}R_{alc}S_{lac}$	-7622.534021	0	
**Isomer B**	g.p.	$S_{ax}S_{alc}R_{lac}$	-7622.531363	1.67	
	Solvation effects with IEF-PCM on non-optimized gas phase structres				
**Isomer A**	Et_2O	$S_{ax}R_{alc}S_{lac}$	-7623.619787	0.0	Non optimized solvation energies
**Isomer B**	Et_2O	$S_{ax}S_{alc}R_{lac}$	-7623.617291	1.6	
**Isomer A**	$^{i}Pr_2O$	$S_{ax}R_{alc}S_{lac}$	-7623.617307	0.0	
**Isomer B**	$^{i}Pr_2O$	$S_{ax}S_{alc}R_{lac}$	-7623.615132	1.4	
**Isomer A**	THF	$S_{ax}R_{alc}S_{lac}$	-7623.62475	0.0	
**Isomer B**	THF	$S_{ax}S_{alc}R_{lac}$	-7623.621561	2.0	
**Isomer A**	Toluene	$S_{ax}R_{alc}S_{lac}$	-7623.612924	0.0	
**Isomer B**	Toluene	$S_{ax}S_{alc}R_{lac}$	-7623.611279	1.0	
	Explicit solvent effects through coordination of different ethers **LacZnBr**				
**LacZnBr + OMe_2**	g.p.	$S_{alc}R_{lac}$		13.1	Fully optimized
**LacZnBr←OMe_2**	g.p. coord.	$S_{alc}R_{lac}$		0.0	
**LacZnBr + OEt_2**	g.p.	$S_{alc}R_{lac}$		13.1	
**LacZnBr←OEt_2**	g.p. coord.	$S_{alc}R_{lac}$		0.0	
**LacZnBr + $O^{i}Pr_2$**	g.p.	$S_{alc}R_{lac}$		13.0	
**LacZnBr← $O^{i}Pr_2$**	g.p. coord.	$S_{alc}R_{lac}$		0.0	
**LacZnBr + THF**	g.p.	$S_{alc}R_{lac}$		14.6	
**LacZnBr←THF**	g.p. coord.	$S_{alc}R_{lac}$		0.0	

In addition to the coordination of protonated phosphoric acid as described in the manuscript (Fig. 1), we also considered a single coordination of Zn^{2+} salt of the deprotonated phosphoric acid of both isomers with the TRIP-catalyst. The relative energies of the intermediates would favour the $S_{ax}S_{alc}R$ isomer (**D**) over the desired $S_{ax}R_{alc}S$ isomer (**C**) by 10 kcal/mol (Figure S02). A linear arrangement of the TRIP-Zn-O(aldehyde)-allyl chain with elongated distances between the chirality-inducing iPr-groups and the lactone moiety was observed due to steric repulsion in case of isomer **D**, which therefore lacks catalytic significance.

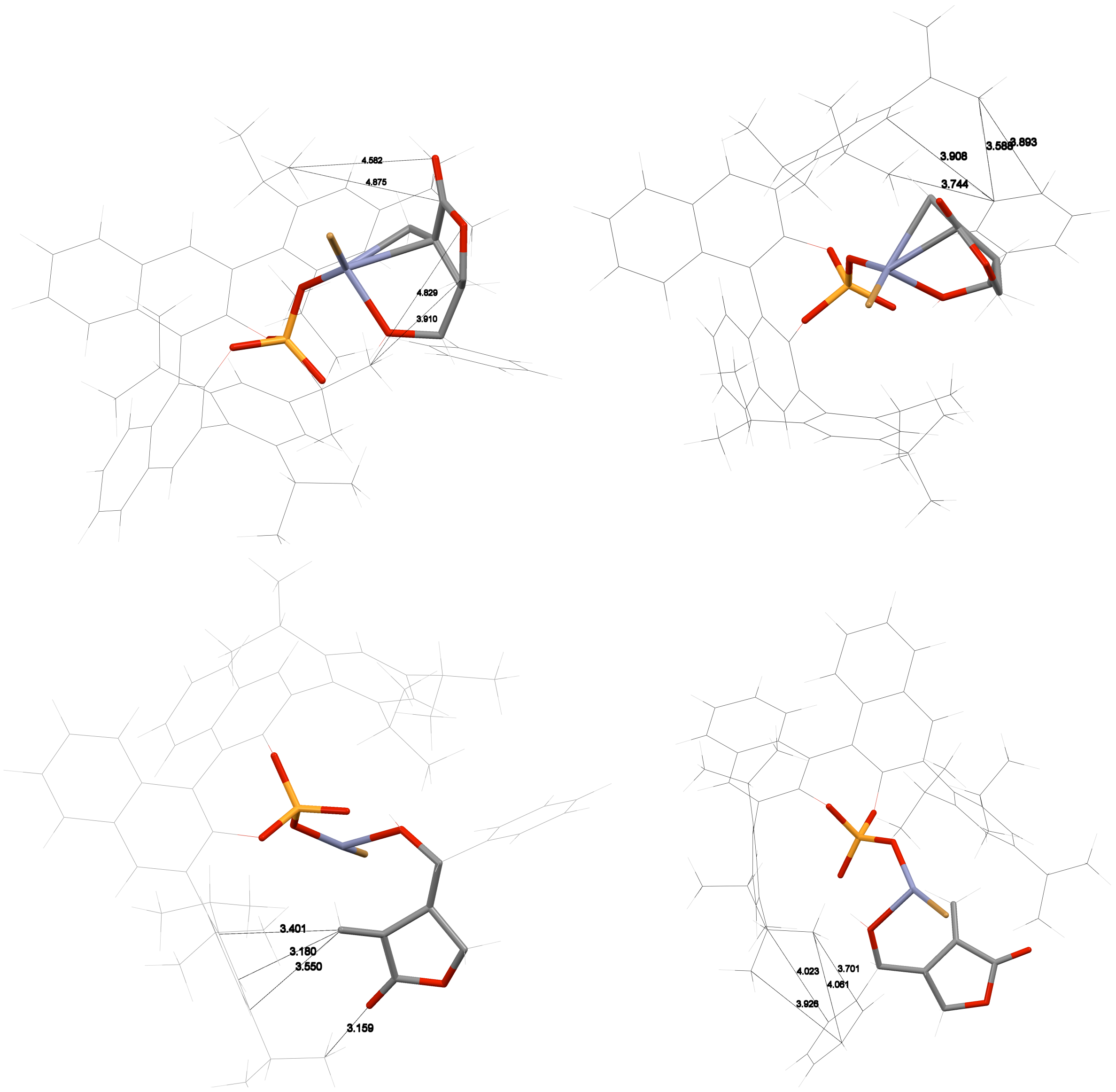

Figure S01. Intramolecular distances of isomers **A** (top, $S_{ax}R_{alc}S_{lac}$) and **B** (bottom, $S_{ax}S_{alc}R_{lac}$) used for the calculation the mean distances between the phenyl substituent of the catalyst and the lactone moiety.

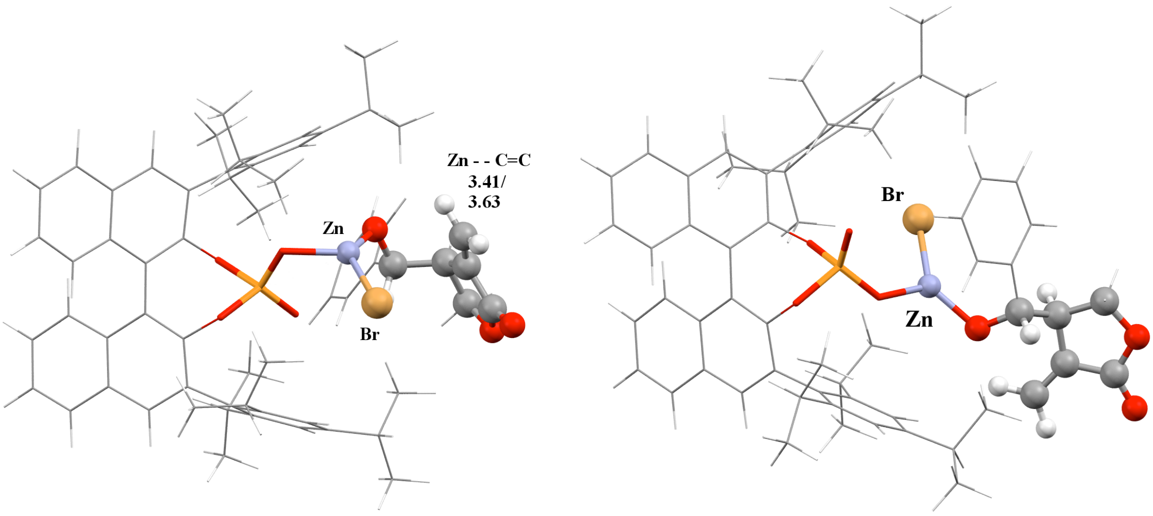

Figure S02. Single coordination involving the Zn^{2+} salt of the deprotonated phosphoric acid [Isomers **C** (left, $S_{ax}R_{alc}S_{lac}$) and **D** (right, $S_{ax}S_{alc}R_{lac}$)].

TRIP

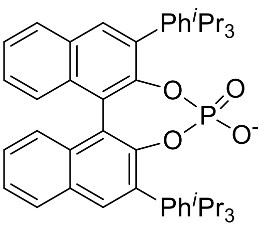

$G_{gas.}(277)$ = -2579.410741 Hartree

C 4.257990 -0.525494 -1.056702
C 3.723904 -0.329898 0.225428
C 4.046137 -1.213079 1.266880
C 4.935299 -2.252388 1.006560
C 5.493433 -2.452750 -0.247085
C 5.136894 -1.581636 -1.268950
C 2.833944 0.833806 0.484230
C 1.483400 0.821653 0.040340
C 0.643503 1.905102 0.222161
C 1.104180 3.015197 0.992984
C 2.460291 3.043963 1.422464
C 3.301770 1.950214 1.122626
C 0.262268 4.083807 1.387474
C 0.744424 5.135450 2.119363
C 2.098461 5.182094 2.501587
C 2.931524 4.151933 2.162722
C -0.716271 1.866325 -0.360098
C -1.545810 0.794635 -0.070131
C -2.909157 0.780163 -0.471030
C -3.373952 1.802002 -1.252963
C -2.538648 2.855097 -1.680312
C -1.186876 2.888013 -1.238571
C -3.016668 3.870707 -2.539580
C -2.191855 4.867650 -2.981047
C -0.841188 4.879991 -2.582795
C -0.353886 3.920807 -1.737007
C -3.826812 -0.304251 -0.021784
C -4.465100 -0.241147 1.227955
C -5.340389 -1.270937 1.574447
C -5.603976 -2.343962 0.739888
C -4.960935 -2.381433 -0.490390
C -4.073726 -1.387648 -0.886821
C -4.249951 0.819847 2.299690
C -3.044836 0.476900 3.174502
C -3.425854 -1.472796 -2.254397
C -3.026165 -2.888397 -2.647393
C -6.529735 -3.453183 1.179237
C -5.748291 -4.734566 1.453671
O -1.091645 -0.216285 0.696863
P 0.005202 -1.348137 0.087428
O -0.621910 -2.086004 -1.025257
O 1.047262 -0.249783 -0.652385
C 3.463643 -1.058179 2.656626
C 2.992300 -2.378976 3.251021
C 6.426904 -3.613942 -0.498037
C 5.788183 -4.645476 -1.422624
C 3.910898 0.384985 -2.216067
C 3.241829 -0.379720 -3.352642
O 0.660130 -1.920144 1.278692
C 4.467406 -0.381168 3.587208
C 5.138624 1.150086 -2.699022
C 7.776820 -3.162248 -1.043164
C -4.351163 -0.876324 -3.313330
C -7.650235 -3.708759 0.178169
C -4.189637 2.278170 1.855191
H -6.991681 -3.130997 2.125069
H -4.057802 3.830289 -2.852400
H -2.568305 5.638718 -3.647233
H -0.177009 5.654303 -2.956515
H 0.691803 3.935793 -1.447173
H 4.344772 1.990941 1.432600
H 3.974984 4.157002 2.470543
H 2.471648 6.024206 3.077410
H 0.073341 5.937188 2.414431
H -0.786291 4.057797 1.108413
H -5.142590 -3.218509 -1.160985
H -5.831634 -1.228610 2.547383
H -2.496418 -0.892136 -2.218977
H -5.139552 0.742691 2.944436
H 5.192101 -2.944679 1.806875
H 5.549651 -1.730169 -2.266330
H 2.575830 -0.420562 2.571392
H 3.188260 1.129162 -1.861849
H 6.603208 -4.099044 0.474015
H 2.474083 -2.190410 4.198499
H 2.285319 -2.861638 2.573240
H 3.830020 -3.054969 3.467509
H 4.042316 -0.258019 4.590542
H 5.377292 -0.989187 3.679847
H 4.763877 0.607922 3.221249
H 2.936462 0.308960 -4.149284
H 3.923691 -1.116560 -3.795341
H 2.351754 -0.903527 -2.992666
H 4.872365 1.843119 -3.506006
H 5.586026 1.729732 -1.883128
H 5.907414 0.468342 -3.084129
H -3.888565 -0.932955 -4.305936
H -4.983771 2.524821 1.142055
H -4.587554 0.173287 -3.110133
H -2.426916 -2.854657 -3.564031
H -2.404874 -3.337276 -1.870335
H -3.900101 -3.522539 -2.850237
H -8.350108 -4.462442 0.558863
H -8.211574 -2.792825 -0.033790
H -7.251742 -4.081739 -0.772625
H -6.408376 -5.529830 1.821324
H -5.263136 -5.094176 0.538457
H -4.961199 -4.565707 2.195077
H -2.968500 1.184276 4.009842
H -3.134699 -0.534044 3.585915
H -2.111095 0.508533 2.605577
H -4.310239 2.923583 2.733665
H -3.231517 2.533250 1.392941
H -4.422731 1.812807 -1.546301
H -5.298081 -1.432169 -3.349572
H 6.440737 -5.517814 -1.550344
H 4.825779 -4.986201 -1.028797
H 5.604175 -4.216617 -2.415005
H 8.458209 -4.013967 -1.157110
H 7.666083 -2.694373 -2.028706
H 8.248669 -2.430017 -0.379858

LacZnBrH

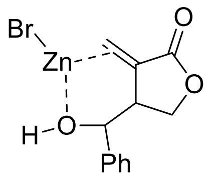

$G_{gas.}(277)$ = -5042.701398Hartree

C 0.186801 -0.397697 0.264509
C -0.252730 0.063001 1.503271
C 0.704486 0.415382 2.471043
C 2.069946 0.262186 2.206776
C 2.487420 -0.229493 0.986272
C 1.541975 -0.527963 0.007233
C -1.721047 0.079636 1.858253
O -1.760014 0.239665 3.287532
Zn -0.217985 -1.145381 3.829059
C -0.742741 -3.014079 2.484934
C -1.888187 -2.318409 2.443717
C -2.394061 -1.265578 1.506034
C -3.895127 -1.281189 1.828838
O -4.019112 -1.790651 3.160209
C -2.927924 -2.488651 3.522172
Br 1.008856 -1.793036 5.576008
H -2.632986 0.474308 3.625502
H -2.245365 0.917416 1.382440
H -0.525481 -0.653164 -0.514530
H 1.867660 -0.877840 -0.966852
H 3.544200 -0.353021 0.777898
H 2.791443 0.541814 2.967659
H 0.410589 1.024808 3.325918
H -2.212900 -1.502483 0.455679
H 0.014400 -2.944538 1.708344
H -0.620070 -3.762992 3.266424
O -2.795014 -3.067637 4.549459
H -4.444453 -1.937012 1.148675
H -4.360527 -0.291826 1.805280

Isomer A

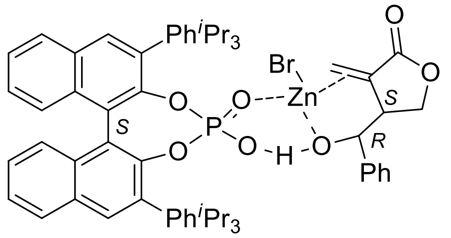

$G_{gas.}(277)$ = -7622.534021 Hartree

C -1.432604 3.648632 1.368706
C -2.349372 3.230698 0.381066
C -2.250693 3.727020 -0.928776
C -1.194539 4.586387 -1.231262
C -0.249709 4.968841 -0.294181
C -0.403466 4.508511 1.008702
C -3.294537 2.120594 0.683746
C -2.929641 0.829780 0.238834
C -3.720314 -0.290382 0.365686
C -4.928678 -0.167397 1.116367
C -5.312006 1.118798 1.592772
C -4.487636 2.238726 1.342290
C -6.515583 1.252672 2.323514
C -7.292854 0.164983 2.606412
C -6.892293 -1.113524 2.173141
C -5.744012 -1.275440 1.447373
O -1.720613 0.713514 -0.416519
P -0.509297 0.010670 0.376339
O -0.049938 0.753032 1.577776
C -3.278364 -1.552944 -0.274129
C -2.010303 -2.038515 -0.041110
C -1.488890 -3.211660 -0.636343
C -2.326940 -3.932355 -1.444624
C -3.623041 -3.472404 -1.769463
C -4.094058 -2.246889 -1.220749
C -5.337737 -1.746000 -1.673464
C -6.096021 -2.447935 -2.570269
C -5.654263 -3.688908 -3.066913
C -4.440587 -4.182710 -2.678612
O -1.186427 -1.373497 0.846164
C -0.067124 -3.584915 -0.397082
C 0.327494 -4.057395 0.865349
C 1.668600 -4.345989 1.093718
C 2.629042 -4.205352 0.097267
C 2.211233 -3.753878 -1.146910
C 0.887852 -3.410226 -1.413502
C -0.686008 -4.315098 1.960890
C -0.903709 -5.815835 2.131631
C 4.077775 -4.561798 0.340411
C 4.675419 -3.829384 1.537003
C 0.528390 -2.870468 -2.785369
C 0.371529 -4.011206 -3.789163
C -3.181976 3.400485 -2.091568
C -4.673434 3.326591 -1.779155
C 0.895984 5.875501 -0.672179
C 0.789312 7.231705 0.016692
C -1.578226 3.222064 2.816639
C -2.522638 4.183689 3.539198
O 0.531141 -0.274098 -0.693903
Zn 2.062335 0.739031 -1.156017
Br 2.418482 2.152840 -2.910290
O 2.336669 1.494032 0.768335
C 3.538433 1.464200 1.514372
C 4.683872 1.282835 0.517945
C 4.473051 0.301869 -0.588197
C 5.297714 0.795317 -1.749330
O 5.718691 2.041017 -1.456900
C 5.079702 2.530862 -0.276385
C 3.798387 -0.848846 -0.658797
O 5.581423 0.196192 -2.735239
C 3.577007 0.400385 2.591821
C 4.603653 0.470750 3.533307
C 4.734160 -0.494521 4.516803
C 3.831063 -1.548080 4.576342
C 2.801749 -1.618543 3.653171
C 2.668756 -0.648913 2.666627

C -0.317667 -3.654850 3.282172
C 1.532526 -1.863188 -3.335694
C 4.244213 -6.070616 0.497491
C -2.744504 2.173546 -2.894007
C 2.241114 5.217425 -0.388812
C -0.268730 3.141298 3.591544
H 0.831832 6.039207 -1.758020
H -6.801022 2.243354 2.668122
H -8.210325 0.280141 3.174854
H -7.498246 -1.979234 2.421911
H -5.443328 -2.267182 1.124705
H -1.977282 -4.863098 -1.886210
H -4.071244 -5.124429 -3.076819
H -6.269857 -4.239393 -3.771290
H -7.043773 -2.040516 -2.908114
H -5.685455 -0.785358 -1.306222
H 0.308816 4.823190 1.767713
H -1.102570 4.956865 -2.252103
H -2.019092 2.217432 2.830297
H -3.062702 4.259433 -2.768622
H 2.953719 -3.653176 -1.937704
H 1.968873 -4.712593 2.074830
H -0.431131 -2.346027 -2.697308
H -1.643689 -3.884090 1.647741
H 4.639241 -4.255392 -0.555652
H 1.140379 -1.393086 -4.242865
H 1.729402 -1.057020 -2.624925
H 2.490388 -2.325079 -3.602964
H 0.086724 -3.626675 -4.774630
H 1.319342 -4.552391 -3.900846
H -0.385527 -4.734958 -3.474688
H -1.126382 -3.781814 4.009871
H 0.582139 -4.102478 3.723427
H -0.149748 -2.579890 3.150707
H -1.672255 -6.014886 2.886809
H -1.221747 -6.276916 1.189943
H 0.019555 -6.314302 2.451228
H -2.680558 3.871086 4.577756

Isomer B

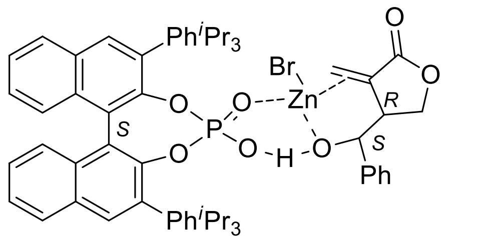

$G_{gas.}(277)$ = -7622.531363 Hartree
C -5.260176 -2.367375 0.298372
C -4.488615 -1.959012 1.384947
C -4.568404 -2.658699 2.582831
C -5.413789 -3.754983 2.699153
C -6.181918 -4.154138 1.616148
C -6.102483 -3.459878 0.414585
C -3.581911 -0.775110 1.249559
O -2.597611 -1.033349 0.227969
Zn -2.128944 0.286706 -1.309758
Br -3.802574 0.334544 -2.852994
C -4.290559 0.488575 0.824141
C -5.452500 1.012346 1.653489

H -4.995037 4.144719 -1.125392
H -3.498766 4.250402 3.050702
H -0.452059 2.697006 4.575547
H 0.462693 2.513754 3.076596
H 0.166440 4.132982 3.765625
H 1.591490 7.901503 -0.312983
H -0.169762 7.713286 -0.199201
H 0.870593 7.126376 1.105259
H 3.067980 5.856018 -0.720891
H 2.372091 5.029016 0.685068
H 2.308849 4.261326 -0.918232
H -3.258416 2.163087 -3.861982
H -1.664867 2.176769 -3.076823
H -2.989198 1.237532 -2.381170
H -5.241388 3.398531 -2.713464
H -4.954148 2.381702 -1.302367
H -4.825937 3.217086 1.676073
H -2.093166 5.193610 3.551642
H 1.505903 1.412267 1.307702
H 3.673871 2.448445 1.995261
H 5.306720 1.301886 3.500590
H 5.537838 -0.421201 5.242765
H 3.929326 -2.308099 5.345102
H 2.090120 -2.437780 3.685173
H 1.834276 -0.713123 1.974938
H 5.300448 -6.339611 0.608595
H 3.712264 -6.429292 1.386944
H 3.842398 -6.606391 -0.368245
H 5.738851 -4.069973 1.643039
H 4.582991 -2.740326 1.445383
H 4.179141 -4.117092 2.471505
H 5.569712 0.942653 1.073625
H 3.252396 -1.284976 0.172808
H 3.858969 -1.415602 -1.585072
H 5.790131 3.169516 0.250991
H 4.202472 3.123364 -0.565932

O -5.863730 2.198733 0.959797
C -4.811460 2.740432 0.294692
C -3.665965 1.796229 0.446148
C -2.403532 2.217610 0.469704
O -4.858296 3.796333 -0.254343
O -0.271054 0.364196 -1.057238
P 0.565495 -0.213902 0.077792
O -0.187526 -0.867226 1.182632
O 1.627316 -1.146346 -0.677572
C 2.725383 -1.598324 0.030934
C 3.742644 -0.710325 0.283271
C 4.858870 -1.183242 1.041442
C 4.929641 -2.565978 1.369355
C 3.878172 -3.435373 0.995364
C 2.757418 -2.977319 0.359547
C 6.042400 -3.047019 2.097394
C 7.027971 -2.198990 2.518447
C 6.935640 -0.823227 2.235771
C 5.882407 -0.328889 1.515651
C 3.651554 0.681139 -0.222930
C 2.546621 1.458716 0.053079
C 2.445393 2.831573 -0.272309
C 3.492291 3.396784 -0.949510

C	4.602240	2.633179	-1.375234
C	4.681804	1.255027	-1.031722
C	5.762285	0.499278	-1.546848
C	6.728050	1.086519	-2.317957
C	6.673678	2.461648	-2.613339
C	5.629218	3.214201	-2.154284
C	1.574052	-3.798314	-0.033746
C	1.436340	-4.246096	-1.360826
C	0.205130	-4.756140	-1.761836
C	-0.888257	-4.834188	-0.908161
C	-0.703272	-4.459758	0.415014
C	0.509256	-3.951538	0.874437
O	1.488391	0.916014	0.754558
C	1.287450	3.649920	0.190310
C	1.258884	4.107437	1.517344
C	0.220141	4.939611	1.924034
C	-0.795917	5.326984	1.059602
C	-0.758538	4.844025	-0.242539
C	0.251309	3.997345	-0.692817
C	2.312472	3.709814	2.530503
C	3.090112	4.920961	3.033744
C	-1.914897	6.239281	1.501487
C	-3.195784	5.485419	1.834574
C	0.184506	3.451351	-2.104456
C	0.760336	4.440638	-3.113882
C	2.563553	-4.303609	-2.381578
C	3.548549	-5.413256	-2.017044
C	-2.238275	-5.242193	-1.450395
C	-3.142576	-5.900751	-0.419748
C	0.673631	-3.623939	2.346699
C	1.301107	-4.815765	3.071032
C	1.693776	2.924621	3.683236
C	-2.251828	7.239507	0.399600
C	-1.233545	3.068724	-2.510823
C	3.312875	-3.009921	-2.698745
C	-2.924454	-4.015493	-2.052556
C	-0.618670	-3.251359	3.063358
H	-2.059882	-5.963921	-2.262132
H	6.086734	-4.107990	2.329420
H	7.873394	-2.579284	3.082995
H	7.704586	-0.147617	2.597315
H	5.818888	0.735304	1.312571
H	3.467875	4.460523	-1.177138
H	5.559140	4.272277	-2.393181
H	7.452511	2.915933	-3.217549
H	7.541672	0.484432	-2.710324
H	5.814291	-0.564127	-1.336438
H	-1.532422	-4.551127	1.110565
H	0.087713	-5.089373	-2.793265
H	1.346419	-2.759954	2.428667
H	2.076952	-4.607621	-3.319300
H	-1.573630	5.110700	-0.912379
H	0.203418	5.289867	2.954712
H	0.783298	2.533297	-2.139271
H	3.035325	3.049013	2.036358
H	-1.562568	6.765258	2.399205
H	-1.220038	2.380956	-3.363142
H	-1.769827	2.589939	-1.682080
H	-1.837770	3.935546	-2.799796
H	0.671549	4.051535	-4.134363
H	0.217487	5.392606	-3.065794
H	1.817725	4.646859	-2.922000
H	2.470131	2.577193	4.373945
H	0.994755	3.544956	4.256712
H	1.148522	2.050169	3.312896
H	3.882775	4.611162	3.723705
H	3.552932	5.464836	2.202834
H	2.439348	5.621595	3.569803
H	1.440326	-4.594930	4.135354
H	3.034431	-6.367774	-1.864795
H	2.270671	-5.096383	2.652969
H	-0.387451	-2.895363	4.072927
H	-1.154854	-2.453705	2.542470
H	-1.288257	-4.114083	3.170735
H	-4.058708	-6.268529	-0.894988
H	-2.650049	-6.745760	0.072339
H	-3.450952	-5.187265	0.353994
H	-3.913276	-4.266978	-2.455653
H	-3.061006	-3.247446	-1.280209
H	-2.328745	-3.577718	-2.860944
H	3.863191	-3.139228	-3.637343
H	2.637218	-2.157660	-2.819165
H	4.050447	-2.757586	-1.929770
H	4.293004	-5.545513	-2.810602
H	4.087013	-5.167895	-1.094346
H	3.967645	-4.491182	1.240453
H	0.641814	-5.689382	2.990136
H	-1.741367	-1.292764	0.662499
H	-5.181447	-1.837327	-0.649325
H	-6.694245	-3.778017	-0.438412
H	-6.842600	-5.010890	1.704703
H	-5.467785	-4.299341	3.636626
H	-3.951977	-2.352772	3.425499
H	-3.095097	7.875289	0.691339
H	-1.393110	7.891465	0.194847
H	-2.512968	6.730315	-0.533890
H	-4.012146	6.190145	2.023207
H	-3.502364	4.832698	1.010380
H	-3.080453	4.867680	2.733680
H	-3.051683	-0.598049	2.200111
H	-4.736285	0.055887	-0.086934
H	-2.172726	3.256970	0.241936
H	-1.577374	1.581585	0.773256
H	-5.128658	1.283761	2.668428
H	-6.304434	0.334112	1.714885

Isomer C

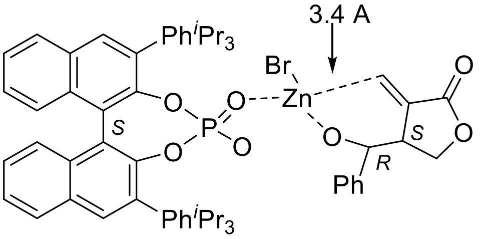

$G_{gas.}$(277) = -7617.751436 Hartree

C	-2.451200	-0.860900	2.955600
C	-3.273300	0.130700	2.407800
C	-3.782100	1.125900	3.252700
C	-3.502200	1.113300	4.618600
C	-2.699600	0.106700	5.160300
C	-2.168600	-0.874600	4.322800
C	-3.609500	0.111800	0.931200
C	-5.149000	0.071700	0.735800
C	-5.556100	0.016300	-0.706400
C	-6.181700	-1.291100	-0.990100
O	-6.294500	-1.991100	0.200400
C	-5.768100	-1.212000	1.326100
C	-5.395600	0.941200	-1.656800
O	-6.587200	-1.752500	-2.049500
O	-3.069200	1.244300	0.261200
Zn	-2.029100	0.718800	-1.176600
O	-0.071400	0.936800	-1.313600
P	0.521000	-0.126600	-0.256600
O	1.534700	0.694900	0.897600
C	2.581400	1.458700	0.380800
C	3.788200	0.841300	0.073700
C	4.855000	1.664400	-0.428100
C	4.678800	3.083400	-0.458400
C	3.417300	3.640600	-0.132300
C	2.339500	2.853200	0.204200
C	6.068600	1.135200	-0.944000
C	7.075400	1.961400	-1.390600
C	6.922600	3.365900	-1.354500
C	5.741900	3.909800	-0.906600
C	3.904100	-0.634000	0.180900
C	2.904000	-1.435300	-0.353500
C	2.888100	-2.853700	-0.263800
C	3.999500	-3.469600	0.260700
C	5.063100	-2.720900	0.827300
C	4.987100	-1.291400	0.860500
C	6.155300	-3.373600	1.455900
C	7.118400	-2.659400	2.129300
C	7.011600	-1.253100	2.218900
C	5.973200	-0.589700	1.604300
C	1.590500	-3.548000	-0.495600
C	0.974700	-3.669400	-1.760700
C	-0.354200	-4.107000	-1.815600
C	-1.087500	-4.433100	-0.679900
C	-0.440800	-4.374600	0.553300
C	0.870400	-3.916500	0.675400
C	1.596900	-3.366500	-3.123900
C	0.969600	-2.114500	-3.764200
C	1.429200	-3.777500	2.088800
C	0.602100	-2.808100	2.943900
C	-2.569200	-4.721900	-0.772500
C	-3.341500	-3.575400	-0.104500

O	1.862600	-0.860300	-1.067900
C	0.947900	3.387500	0.215700
C	0.367700	3.789400	-1.014200
C	-0.960400	4.219500	-1.013000
C	-1.745600	4.233000	0.141500
C	-1.166600	3.803000	1.335000
C	0.167500	3.383900	1.389600
C	1.111900	3.760100	-2.342200
C	0.294600	3.139300	-3.482700
C	-3.159100	4.773700	0.049500
C	-3.134200	6.303500	0.194400
C	0.751200	2.958700	2.724900
C	-0.089700	1.874300	3.402400
O	-0.559500	-1.046900	0.438600
C	1.554000	5.177700	-2.740900
C	-4.128600	4.149800	1.050000
C	0.932300	4.176300	3.642400
C	1.542800	-5.149600	2.767200
C	-2.956900	-6.074700	-0.169000
C	3.122200	-3.294900	-3.195500
Br	-2.528900	-0.713800	-2.937400
H	-3.205400	-0.831600	0.517300
H	-2.839500	-4.725300	-1.840200
H	6.199500	-4.460100	1.407900
H	7.946500	-3.171900	2.611600
H	7.749600	-0.691400	2.785200
H	5.893500	0.489400	1.691800
H	3.281900	4.716200	-0.231900
H	5.587700	4.987300	-0.908900
H	7.727300	4.007900	-1.702700
H	7.991400	1.529100	-1.784300
H	6.194400	0.058400	-0.993600
H	-0.995600	-4.625300	1.457500
H	-0.842600	-4.159700	-2.789300
H	2.440600	-3.358700	2.038700
H	1.300000	-4.224300	-3.754400
H	-1.415700	4.539800	-1.951400
H	-1.772400	3.751300	2.238500
H	2.009900	3.140100	-2.221700
H	1.745800	2.532700	2.542500
H	-3.524600	4.533200	-0.963900
H	0.915500	3.073500	-4.386400
H	-0.038700	2.130100	-3.219300
H	-0.582200	3.751900	-3.735100
H	2.128800	5.154500	-3.676500
H	0.674400	5.818400	-2.898300
H	2.172400	5.648300	-1.966100
H	0.435100	1.477100	4.282100
H	-1.059700	2.263100	3.741500
H	-0.286700	1.042700	2.713500
H	1.380700	3.875700	4.599200
H	1.581400	4.928900	3.174700
H	-0.036500	4.649200	3.854900
H	1.988400	-5.050000	3.766300
H	3.600000	-4.151000	-2.701900
H	2.166300	-5.832200	2.174300
H	1.053700	-2.705500	3.940900
H	0.543400	-1.818100	2.474300
H	-0.428300	-3.171400	3.072300
H	-4.035900	-6.245700	-0.275100
H	-2.422500	-6.899700	-0.656900
H	-2.719000	-6.105100	0.903400

H	-4.422000	-3.653800	-0.282100
H	-3.165100	-3.581200	0.982400
H	-2.979000	-2.615600	-0.497100
H	1.337200	-1.995200	-4.793000
H	-0.125300	-2.170300	-3.789600
H	1.230000	-1.217900	-3.190500
H	3.430300	-3.291100	-4.249900
H	3.507800	-2.376600	-2.739100
H	4.023600	-4.556900	0.333100
H	0.554300	-5.614900	2.883000
H	-4.142500	6.725600	0.084700
H	-2.754400	6.573400	1.190300
H	-2.477000	6.765800	-0.553400
H	-5.152800	4.495600	0.854400
H	-4.074100	3.057000	0.959400
H	-3.873500	4.437500	2.081000
H	-5.559400	0.967000	1.221700
H	-4.941200	1.903100	-1.437400
H	-5.698400	0.726500	-2.679300
H	-6.600400	-1.015400	2.008200
H	-5.028900	-1.839600	1.833900
H	-2.001900	-1.597600	2.289300
H	-1.521100	-1.648700	4.729000
H	-2.480000	0.095900	6.225200
H	-3.908300	1.891000	5.261600
H	-4.398300	1.921100	2.837200

Isomer D

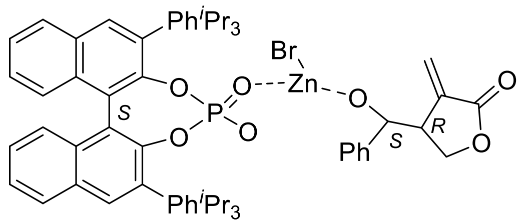

$G_{gas.}(277)$ = -7617.77002 Hartree

C	3.009400	1.179000	1.874100
C	4.030800	1.596300	0.997400
C	4.181900	2.962100	0.751100
C	3.322500	3.887700	1.346900
C	2.285700	3.459900	2.172600
C	2.129200	2.098500	2.440700
C	4.761900	0.492000	0.253500
C	5.877800	0.972300	-0.681900
C	6.607400	-0.173100	-1.339800
C	8.064500	0.018600	-1.180500
O	8.281800	1.137800	-0.401000
C	7.012600	1.708500	0.061700
O	8.990800	-0.651500	-1.624200
O	3.804900	-0.263100	-0.485100
Zn	2.043800	0.386100	-0.349000
Br	1.120200	2.313900	-1.290600
O	0.723600	-0.627300	0.609400
P	-0.705300	-0.290300	1.244100
O	-0.846400	0.525600	2.583700
O	-1.543100	-1.807000	1.423800
C	-2.086400	-2.469700	0.334100
C	-3.290000	-2.035000	-0.197300
C	-3.884800	-2.804200	-1.257700
C	-3.270100	-4.037800	-1.643700
C	-2.020300	-4.399500	-1.083600
C	-1.386800	-3.606200	-0.155100
C	-5.039200	-2.388200	-1.974800
C	-5.590300	-3.175100	-2.961400
C	-5.014300	-4.421400	-3.295200
C	-3.870400	-4.833500	-2.653400
C	-3.852800	-0.728300	0.217900
C	-3.056800	0.412200	0.145000
C	-3.596300	1.724800	0.209600
C	-4.930700	1.860900	0.520200
C	-5.755700	0.740800	0.779900
C	-5.218600	-0.574500	0.625500
C	-7.109600	0.897200	1.175700
C	-7.900600	-0.195800	1.440100
C	-7.360800	-1.498100	1.325500
C	-6.055900	-1.681900	0.927900
C	-2.753700	2.910100	-0.111900
C	-2.221500	3.681600	0.946000
C	-1.443600	4.802700	0.649100
C	-1.155400	5.165100	-0.666400
C	-1.690100	4.389500	-1.690700
C	-2.490000	3.265800	-1.453100
C	-2.504800	3.338200	2.400200
C	-3.660300	4.191000	2.943800
C	-2.971200	2.521300	-2.698400
C	-4.451700	2.120800	-2.726800
C	-0.250000	6.338500	-0.977500
C	-0.794300	7.652700	-0.406700
O	-1.687100	0.296300	-0.090200
C	0.047900	-3.803700	0.179000
C	1.010500	-3.462000	-0.802500
C	2.361100	-3.618900	-0.492200
C	2.789900	-4.103600	0.744600
C	1.825400	-4.412800	1.702300
C	0.459700	-4.258000	1.444200
C	0.649800	-2.917100	-2.180600
C	1.178800	-3.840100	-3.287100
C	4.276300	-4.180100	1.024500
C	4.756100	-2.795500	1.481500
C	-0.537800	-4.594100	2.538600
C	-0.464100	-6.077700	2.923500
C	1.142800	-1.477000	-2.390500
C	4.671600	-5.259800	2.030600
C	-0.339800	-3.697500	3.766900
C	-2.079500	1.319900	-3.055200
C	1.171900	6.070600	-0.468100
C	-1.269200	3.494700	3.289600
H	5.245800	-0.168000	1.004600
H	-0.203800	6.434600	-2.074400
H	-7.502700	1.907200	1.276700
H	-8.934000	-0.064200	1.749500
H	-7.979200	-2.359700	1.562300
H	-5.642900	-2.684100	0.855800
H	-1.509400	-5.283900	-1.463900
H	-3.388300	-5.772400	-2.920000
H	-5.463100	-5.036500	-4.070600
H	-6.471500	-2.828900	-3.494700
H	-5.491000	-1.429900	-1.742500
H	-1.032400	5.392900	1.467900
H	-1.455500	4.650100	-2.723800
H	-2.792000	2.279200	2.452500
H	-2.842900	3.250100	-3.516700

H	3.117400	-3.323400	-1.220800
H	2.137200	-4.774400	2.681300
H	-0.443200	-2.888000	-2.276000
H	-1.547200	-4.402800	2.150300
H	4.775400	-4.403500	0.066500
H	1.033700	-1.177100	-3.441200
H	0.530200	-0.787600	-1.798300
H	2.206000	-1.370600	-2.120700
H	0.839200	-3.490600	-4.270900
H	2.277000	-3.857700	-3.297700
H	0.827300	-4.870400	-3.143200
H	-1.093300	-3.925700	4.532800
H	0.652500	-3.854200	4.212400
H	-0.437700	-2.641000	3.491800
H	-1.221400	-6.315800	3.682500
H	-0.632600	-6.721200	2.050500
H	0.520900	-6.327200	3.341400
H	-3.855900	3.948900	3.997200
H	-5.101700	2.940800	-2.394200
H	-4.585700	4.029600	2.376600
H	-1.457900	3.047000	4.273300
H	-0.425300	2.965100	2.836700
H	-1.003800	4.551600	3.442200
H	-0.140300	8.493800	-0.673800
H	-1.803600	7.862000	-0.782300
H	-0.846700	7.604000	0.689800
H	1.856700	6.874400	-0.773200
H	1.176000	6.015600	0.630500
H	1.544200	5.109200	-0.843900
H	-2.350300	0.941800	-4.051200
H	-1.021100	1.607300	-3.049600
H	-2.192800	0.504100	-2.331800
H	-4.732400	1.852000	-3.754400
H	-4.652100	1.250300	-2.091900
H	-5.369100	2.857600	0.551800
H	-3.406500	5.259200	2.877900
H	5.764600	-5.323900	2.105800
H	4.290100	-5.022400	3.033400
H	4.284200	-6.245700	1.743600
H	5.851100	-2.759200	1.569500
H	4.419300	-2.036500	0.762000
H	4.317900	-2.555800	2.462100
H	2.904100	0.118600	2.104600
H	1.311900	1.742800	3.064400
H	1.587900	4.179800	2.594100
H	3.440900	4.946300	1.127900
H	4.934400	3.318500	0.052200
C	6.108200	-1.240300	-1.970300
H	5.034100	-1.373000	-2.051000
H	6.784800	-1.987800	-2.380300
H	5.414800	1.617800	-1.444700
H	6.956000	1.550800	1.145900
H	7.057100	2.780800	-0.140900

LacZnBr

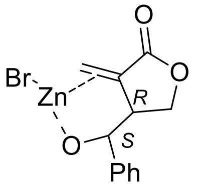

$G_{gas.}(298)$ = -5042.397646Hartree

C	-2.387570	-1.721640	0.793890
C	-2.428900	-0.206200	0.966220
C	-3.751370	-0.029150	1.594950
C	-4.103140	-1.321630	2.261760
O	-3.241120	-2.257250	1.817630
C	-1.305090	0.310230	1.951340
O	-1.535900	1.610490	2.316530
Zn	-3.025100	1.985840	3.260170
Br	-4.384150	2.914720	4.760860
C	-4.526220	1.057980	1.681500
O	-4.968950	-1.526390	3.053960
C	0.034280	0.183520	1.268940
C	0.419590	1.138770	0.332460
C	1.635930	1.029000	-0.320350
C	2.484700	-0.035950	-0.042780
C	2.113320	-0.984620	0.896720
C	0.892780	-0.872120	1.551080
H	-1.311210	-0.393050	2.813670
H	-5.472440	1.001780	2.212710
H	-4.320240	1.938200	1.076770
H	-2.314120	0.344050	0.026850
H	-1.392580	-2.150090	0.930370
H	-2.777280	-2.035530	-0.179580
H	0.613630	-1.603940	2.306700
H	2.778120	-1.810650	1.130100
H	3.439710	-0.118740	-0.552260
H	1.930220	1.781560	-1.045780
H	-0.237650	1.982970	0.145770

LacZnBr←Me$_2$O

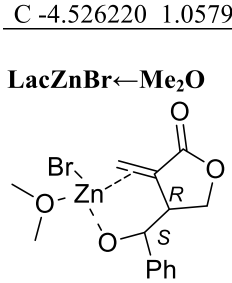

$G_{gas.}(298)$ = -5197.324052Hartree

C	0.559300	1.141560	0.210420
C	0.327480	0.211390	1.220240
C	1.184610	-0.875610	1.343270
C	2.244130	-1.046440	0.460280
C	2.458410	-0.123580	-0.551140
C	1.614440	0.974350	-0.670520
C	-0.845560	0.405020	2.151880
C	-2.154140	-0.117210	1.420430
C	-3.339010	0.149280	2.256710
C	-3.595960	-1.080930	3.064050
O	-2.861820	-2.076380	2.539500
C	-2.189760	-1.639760	1.344620
C	-4.082580	1.256680	2.371750
Zn	-2.352950	2.187010	3.631980
Br	-3.460750	3.859380	4.703610

O -4.317310 -1.209620 4.007920
H -0.705970 -0.279370 3.019420
O -0.972070 1.710850 2.515670
H -4.935630 1.263720 3.044840
H -3.976300 2.077720 1.667560
H -2.196880 0.372030 0.442000
H -1.199660 -2.099570 1.334140
H -2.758430 -2.000160 0.481720
H 1.035040 -1.587610 2.153080
H 2.908760 -1.897970 0.571290
H 3.287480 -0.252050 -1.240220
H 1.787580 1.708260 -1.452130
H -0.088020 2.011350 0.145710
O -1.963210 0.819720 5.208210
C -2.952810 0.611730 6.210120
H -2.621130 -0.177790 6.893540
H -3.135020 1.540860 6.763580
H -3.862620 0.288410 5.702600
C -0.704660 1.191840 5.749740
H -0.023660 1.336940 4.907690
H -0.795170 2.131980 6.311630
H -0.325340 0.405440 6.411460

LacZnBr←Et_2O

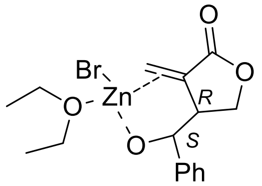

$G_{gas.}(298)$ = -5275.861348 Hartree

C 0.002245 -0.152551 -0.053236
C -0.018607 -0.114874 1.424545
C 1.471487 -0.021297 1.735913
O 2.150238 -0.612596 0.615267
C 1.353898 -0.632703 -0.469889
C -0.669055 -1.440417 1.973424
O -1.926999 -1.600979 1.470633
Zn -2.096446 -1.912134 -0.337618
C -0.957603 0.150339 -0.932528
O 1.715895 -0.977059 -1.554264
C -0.693955 -1.404997 3.482812
C 0.273129 -2.055978 4.240254
C 0.242252 -1.999912 5.628502
C -0.765140 -1.296240 6.269670
C -1.744748 -0.656935 5.518910
C -1.711295 -0.713649 4.135432
Br -3.491346 -1.971373 -2.141421
O -1.026575 -3.730729 -0.362885
C -1.485521 -4.588762 0.693189
C -2.952360 -4.918436 0.571516
C -0.835615 -4.388675 -1.625142
C 0.004856 -3.507182 -2.501796
H 0.029426 -2.256925 1.672692
H -0.760543 0.063402 -1.997705
H -1.873367 0.642980 -0.618102
H -0.599744 0.721446 1.827104
H 1.766586 -0.571525 2.631586
H 1.808833 1.015791 1.831215
H 1.051368 -2.628186 3.737772
H 1.001818 -2.515396 6.208482
H -0.795141 -1.253773 7.354155
H -2.543852 -0.117415 6.018739
H -2.487296 -0.245333 3.537147
H -0.341646 -5.349423 -1.428395
H -1.813221 -4.585038 -2.085124
H -1.297001 -4.038260 1.618368
H -0.862394 -5.491981 0.687731
H 0.150340 -3.978432 -3.477259
H -0.490065 -2.546397 -2.674874
H 0.981816 -3.309689 -2.055493
H -3.257920 -5.572645 1.392606
H -3.558602 -4.009008 0.635419
H -3.188655 -5.421834 -0.370310

LacZnBr←$^{i}Pr_2O$

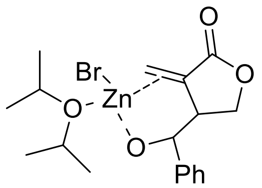

$G_{gas.}(298)$ = -5354.402981 Hartree

C 0.019168 -0.032768 0.012496
C 0.009703 -0.009250 1.402543
C 1.222694 0.015638 2.085855
C 2.419998 0.038954 1.390003
C 2.421521 0.029325 0.000247
C 1.219070 -0.011115 -0.688218
C -1.280934 -0.012758 2.188846
O -1.188576 -0.850427 3.261736
Zn -2.691067 -1.280932 4.239492
Br -3.360165 -1.925629 6.326295
C -1.608592 1.450987 2.652581
C -1.960121 2.368542 1.485780
O -3.385549 2.267317 1.320815
C -3.963765 1.819374 2.449946
C -2.881064 1.456775 3.409585
C -3.107993 1.196171 4.698550
O -5.148179 1.735171 2.594195
O -3.762646 -2.318026 2.773285
C -5.179273 -2.184674 2.510066
C -5.768228 -1.335684 3.604863
C -3.111091 -3.488894 2.218224
C -2.300707 -4.133324 3.319786
C -2.254042 -3.131109 1.025019
C -5.399748 -1.600172 1.133954
H -2.099722 -0.294179 1.484381
H -4.117602 1.238423 5.098308
H -2.289764 1.064375 5.400238
H -0.755980 1.806828 3.240458
H -1.496033 2.076654 0.541672
H -1.713903 3.414683 1.693881
H -0.925054 -0.085938 -0.527829

H 1.212910 -0.033729 -1.773825
H 3.360927 0.043458 -0.543955
H 3.360547 0.057881 1.932731
H 1.207327 -0.008592 3.171567
H -5.605720 -3.197815 2.565566
H -3.918920 -4.169564 1.912842
H -6.835815 -1.183444 3.423365
H -5.641018 -1.800285 4.584800
H -5.312544 -0.340126 3.607188
H -1.862253 -5.071595 2.967191
H -1.467385 -3.481654 3.610654
H -2.915231 -4.343112 4.199276
H -1.853275 -4.045492 0.575311
H -2.819450 -2.594077 0.257869
H -1.410392 -2.512111 1.342784
H -6.468350 -1.448297 0.954479
H -4.906452 -0.624072 1.054797
H -5.016173 -2.255374 0.346981

LacZnBr←THF

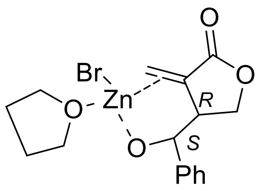

$G_{gas.}(298)$ = -5274.674423 Hartree

C 0.279980 0.127730 -0.024950
C 0.218350 0.059540 1.362220
C 1.393730 0.187110 2.096860
C 2.603120 0.398720 1.455610
C 2.654960 0.481420 0.069080
C 1.491530 0.341500 -0.670910
C -1.091290 -0.144880 2.086910
O -0.942260 -0.978570 3.151440
Zn -2.447820 -1.382040 4.129270
Br -3.251290 -2.518810 5.935100
C -1.628250 1.277220 2.554300
C -2.130890 2.115130 1.383890
O -3.528150 1.801360 1.247470
C -4.011790 1.280590 2.387990
C -2.869230 1.112660 3.334800
C -3.047010 0.815880 4.628410
O -5.163310 1.017490 2.567670
O -3.765110 -1.936010 2.588630
C -5.188470 -2.079990 2.841070
C -5.561100 -3.445630 2.290840
C -3.266850 -3.126220 1.947790
C -4.241680 -4.204980 2.353330
H -1.836860 -0.512500 1.345190
H -4.056790 0.732320 5.022170
H -2.219550 0.853650 5.332120
H -0.820110 1.752680 3.119290
H -1.643970 1.880090 0.435680
H -2.039240 3.189360 1.573210
H -0.628010 -0.010590 -0.609290
H 1.526140 0.387950 -1.755280
H 3.603990 0.643650 -0.432760
H 3.514780 0.492990 2.038250
H 1.342260 0.090660 3.177320
H -5.701910 -1.241070 2.367950
H -5.335160 -2.024820 3.926230
H -2.236030 -3.278630 2.282250
H -3.260710 -2.969570 0.860760
H -6.356750 -3.915790 2.872510
H -5.902280 -3.365250 1.252310
H -4.209410 -5.076210 1.695470
H -4.036070 -4.527990 3.380570

Me_2O

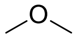

$G_{gas.}(298)$ = -154.907604 Hartree

H -0.002800 0.153545 0.057577
C 0.039408 -0.030964 1.132818
O 1.380126 -0.156174 1.499061
C 1.518972 -0.393978 2.867138
H 1.098040 0.429164 3.468621
H -0.531452 -0.947525 1.357665
H -0.448820 0.808482 1.655731
H 2.584213 -0.481520 3.089643
H 1.015636 -1.327003 3.171465

Et_2O

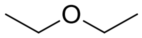

$G_{gas.}(298)$ = -154.907604 Hartree

C 0.017399 0.049350 0.016006
C 0.004988 -0.075222 1.516650
O 1.337289 -0.084423 1.963728
C 1.490359 -0.409601 3.322876
C 1.020543 0.679267 4.265114
H -0.501364 -1.006962 1.828798
H -0.558924 0.760477 1.964152
H 2.560923 -0.591196 3.464382
H 0.966933 -1.357227 3.543174
H -0.999755 0.071841 -0.385222
H 0.528123 0.969429 -0.281861
H 0.551987 -0.793435 -0.430989
H 1.291359 0.436249 5.297192
H 1.489718 1.632339 4.001531
H -0.065106 0.812238 4.232958

$^{i}Pr_2O$

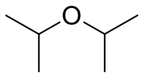

$G_{gas.}(298)$ = -311.986169 Hartree

C -0.046058 0.465925 0.148441
C -0.043975 -0.057400 1.572035
C 1.355570 -0.310019 2.081155
O -0.735880 -1.292366 1.638737
C -2.137956 -1.195906 1.821137
C -2.466807 -1.099663 3.298664
C -2.749201 -2.420911 1.182802
H -0.540005 0.677648 2.231313
H -2.514511 -0.294663 1.304495
H 1.966383 0.596623 2.025925
H 1.331611 -0.659191 3.117086

H 1.831509 -1.086277 1.471840
H -3.545774 -1.002723 3.459902
H -2.118306 -2.003804 3.810058
H -1.976992 -0.237176 3.762281
H -3.835664 -2.435680 1.315181
H -2.521488 -2.452557 0.113777
H -2.331278 -3.321506 1.646079
H 0.466352 1.431463 0.079541
H 0.468244 -0.248377 -0.504025
H -1.065685 0.596825 -0.228324

THF

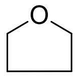

$G_{gas.}(298) = -232.25595$ Hartree

C 0.059124 0.014131 0.012867
C 0.018444 -0.019292 1.530995
C 1.503831 0.010995 1.860637
C 2.060032 -0.886773 0.768783
O 1.232483 -0.686851 -0.365452
H 1.892711 1.030858 1.752087
H 1.741417 -0.340841 2.867690
H 2.018770 -1.943949 1.076628
H 3.096530 -0.655027 0.500670
H -0.808798 -0.461706 -0.456398
H 0.113406 1.052604 -0.351455
H -0.425657 -0.959197 1.880968
H -0.548260 0.809206 1.963234

References

[1] D. M. Hodgson, E. P. A. Talbot, B. P. Clark, *Org. Lett.* **2011**, *13*, 2594-2597.

[2] S.-C. Tsai, J. P. Klinmann, *Bioorg. Chem.* **2003**, *31*, 172-190.

[3] Y. Gao, X. Wang, L. Sun, L. Xie, X. Xu, *Org. Biomol. Chem.* **2012**, *10*, 3991-3998.

[4] J. Fischer, A. J. Reynolds, L. A. Sharp, M. S. Sherburn, *Org. Lett.* **2004**, *6*, 1345-1348.

[5] M. J. Frisch, G. W. Trucks, H. B. Schlegel, G. E. Scuseria, M. A. Robb, J. R. Cheeseman, G. Scalmani, V. Barone, B. Mennucci, G. A. Petersson, H. Nakatsuji, M. Caricato, X. Li, H. P. Hratchian, A. F. Izmaylov, J. Bloino, G. Zheng, J. L. Sonnenberg, M. Hada, M. Ehara, K. Toyota, R. Fukuda, J. Hasegawa, M. Ishida, T. Nakajima, Y. Honda, O. Kitao, H. Nakai, T. Vreven, J. A. Montgomery, J. E. Peralta, F. Ogliaro, M. Bearpark, J. J. Heyd, E. Brothers, K. N. Kudin, V. N. Staroverov, R. Kobayashi, J. Normand, K. Raghavachari, A. Rendell, J. C. Burant, S. S. Iyengar, J. Tomasi, M. Cossi, N. Rega, J. M. Millam, M. Klene, J. E. Knox, J. B. Cross, V. Bakken, C. Adamo, J. Jaramillo, R. Gomperts, R. E. Stratmann, O. Yazyev, A. J. Austin, R. Cammi, C. Pomelli, J. W. Ochterski, R. L. Martin, K. Morokuma, V. G. Zakrzewski, G. A. Voth, P. Salvador, J. J. Dannenberg, S. Dapprich, A. D. Daniels, Ö. Farkas, J. B. Foresman, J. V. Ortiz, J. Cioslowski, D. J. Fox, Gaussian Inc., Wallingford CT, 2009.

[6] Y. Zhao, D. G. Truhlar, *Theor. Chem. Acc.* **2008**, *120*, 215-241.

[7] G. Scalmani, M. J. Frisch, *J. Chem. Phys.* **2010**, *132*, 114110-114124.
